# Immune Repertoire Profiling Reveals that Clonally Expanded B and T Cells Infiltrating Diseased Human Kidneys Can Also Be Tracked in Blood

**DOI:** 10.1371/journal.pone.0143125

**Published:** 2015-11-23

**Authors:** Johannes Weinberger, Raul Jimenez-Heredia, Susanne Schaller, Susanne Suessner, Judith Sunzenauer, Roman Reindl-Schwaighofer, Richard Weiss, Stephan Winkler, Christian Gabriel, Martin Danzer, Rainer Oberbauer

**Affiliations:** 1 Ludwig Boltzmann Institute for Experimental and Clinical Traumatology, Linz, Austria; 2 Department of Immunogenetics, Red Cross Transfusion Service of Upper Austria, Linz, Austria; 3 Bioinformatics Research Group, University of Applied Sciences Upper Austria, Hagenberg, Austria; 4 Department of Internal Medicine III, KH Elisabethinen, Linz, Austria; 5 Department of Internal Medicine III, Medical University of Vienna, Vienna, Austria; 6 Department of Molecular Biology, University of Salzburg, Salzburg, Austria; University of Leeds, UNITED KINGDOM

## Abstract

Recent advances in high-throughput sequencing allow for the competitive analysis of the human B and T cell immune repertoire. In this study we compared Immunoglobulin and T cell receptor repertoires of lymphocytes found in kidney and blood samples of 10 patients with various renal diseases based on next-generation sequencing data. We used Biomed-2 primer panels and ImmunExplorer software to sequence, analyze and compare complementarity determining regions and V-(D)-J elements. While generally an individual’s renal receptor repertoire is different from the repertoire present in blood, 94% (30/32) of the lymphocytes with clonal expansion in kidney can also be traced in blood however, not all of these clonotypes are equally abundant. Summarizing the data of all analyzed patients, 68% of highly expanded T cell clonotypes and 30% of the highly expanded B cell clonotypes that have infiltrated the kidney can be found amongst the five most abundant clonotypes in blood. In addition, complementarity determining region 3 sequences of the immunoglobulin heavy chains are on average more diverse than T cell receptor beta chains. Immune repertoire analysis of tissue infiltrating B and T cells adds new approaches to the assessment of adaptive immune response in kidney diseases. Our data suggest that expanded clonotypes in the tissues might be traceable in blood samples in the course of treatment or the natural history of the disease.

## Introduction

The adaptive immune system shields the human body from a large variety of potential pathogens. This protection is mediated by B and T lymphocytes and their receptors that bind pathogen derived antigens as well as major histocompatibility complex (MHC) bound peptides. During the development of B and T cells, the variable antigen receptor gene segments are rearranged through targeted DNA recombination events. Additional sequence complexity is introduced by the addition or removal of nucleotides at the junctions of these segments. Especially the gene sequences in complementarity determining regions (CDR), notably CDR3, contribute most to Immunoglobulin (IG) and T cell receptor (TR) diversity [1, 2].

Apart from receptor specificity, B and T cells can differentiate into several cell subtypes covering a wide range of different tasks. Besides their potential to differentiate into antibody secreting plasma cells, B cells can function as antigen-presenting or immune-regulatory cells [3]. They are also involved in the formation of local lymphoid tissue [4]. T helper cells (CD4^+^) conduct the immunological response via cytokine release and cytotoxic T cells (CD8^+^) directly attack cells presenting foreign antigens via MHC-I.[5, 6] According to the clonal theory of adaptive immunity, antigen recognition through specific B or T cell receptors results in the clonal expansion of all antigen specific lymphocyte subtypes thereby explaining the highly dynamic nature of B and T cell diversity.

In recent years, next-generation sequencing has become part of the study of the immune repertoire. The technology enables in-depth analysis of rearranged IG and TR loci that is incomparable with regards to sensitivity. The rearranged V-(D)-J regions are short enough (around 500bp, including CDR 1, 2 and 3) to be a perfect target for high-throughput sequencing methods.[7–9] The technology was recently applied to several studies that contributed greatly to extend the community’s knowledge of the nature of IG and TR clonality and diversity [10]. Heather Morris and her group, for example, recently published a study which revealed that donor-reactive T cells are reduced in tolerant kidney transplant patients, while this is not the case in non-tolerant patients [11].

As there is high prevalence of B and T cell expansion and due to the fact that diversity plays an important role after organ transplantation, a number of studies have been conducted to investigate lymphocyte repertoires related to kidney diseases. Referring to most important findings of recent studies it can be assumed that lymphocytes that are infiltrating the site of inflammation, undergo local clonal expansion and have a major impact on disease progression [4, 12–14].

The clearly shown connection of organ health and B and T cell diversity and clonality is a tremendous encouragement for the use of this technology as a potential biomarker. Combining comprehensive FACS sorting with IG and TR repertoire sequencing could even allow us to determine the cell subtype of specific highly expanded B or T cells and open doors for personalized treatment.

However, one has to deal with a practical issue for this analysis as it would require a tissue sample for every assay. By comparing the lymphocyte repertoire in blood and kidney samples, we evaluated if an analysis of blood derived immune repertoire reflects tissue specific immunological activity in renal disease including kidney transplantation. For an initial proof of concept, we analyzed the lymphocyte repertoire in large pieces of kidney tissue following nephrectomy to reduce sampling bias in small volume needle biopsies. Contrasting most studies in the field we decided not to focus on B or T cells but to analyze both cell types simultaneously instead. We believe that scientific output can be significantly increased by combining B and T cell data due to the mutual dependency of B and T cells in immunological responses.

## Materials and Methods

### Patient cohort

The study population consisted of 10 patients who underwent nephrectomy at the Elisabethinen Hospital (Linz, Austria) (age range 24 to 81 years). Basic characteristics and individual diseases are shown in S1 Table. Informed written consent was obtained from all participating patients according to the Declaration of Helsinki. Ethical approval for the sample collection with the number E-9-12 was obtained from the Ethical Committee of Upper Austria on 2013-01-21.

### Sample processing

Surgical wedge biopsies (size approx. 1x1x2cm), 9ml EDTA-blood and 40 ml of urine were obtained from every patient. We incised the tissues with a scalpel and rinsed them in PBS with a sterile syringe and needle. The resulting cell suspension was filtered with a 40μm nylon cell strainer (BD Biosciences, Franklin Lakes, NJ). Mononuclear cells (MNCs) were isolated from the tissue cell suspension and from the EDTA-blood by Ficoll density gradient (GE Healthcare, Little Chalfond, U.K.) according to manufacturer’s protocol. IG and TR amplification from urine samples with standard protocols were unsuccessful.

### Flow cytometry

For flow cytometry (FCM) analyses, 50μl of twice-washed cells from MNC fractions (starting material blood or kidney tissue samples) were stained for 20 minutes with 5μl of each antibody followed by 3μl of conjugated antibodies at room temperature using concentrations recommended by the suppliers (BD Biosciences, BD Pharmingen and eBioscience). For the gating strategy and a detailed list of antibodies used see S2 Table and S1–S3 Figs. Paired two-tailed t-tests were performed to compare different cell types and compartment (blood vs. kidney) values with a level of significance of 0.05.

### Immunomagnetic Isolation

MNCs derived from one patient with acute allograft rejection (patient 10) were sequentially sorted using EasySep^™^ (Stemcell Technologies, Vancouver, Canada) in different subpopulations: CD4^+^, CD8^+^ and a combination of CD19^+^, CD20^+^ and CD138^+^, according to manufacturer’s instructions.

### Sequencing: From DNA extraction to MiSEQ sequencing

Genomic DNA was extracted from MNCs using MagNAPure Compact (Roche Diagnostics, Basel, Switzerland) according to the supplier’s instructions. First amplification of rearranged V-(D)-J elements was performed in a final volume of 100μl as follows: 95°C for 10 min, 35 cycles of 95°C for 30 s, 58°C for 45 s and 72°C for 45 s, and 72°C for 10 min. The PCR mixture contained 5U of AmpliTaq Gold polymerase (Applied Biosystems, Foster City, CA), 0.2 mM of each dNTP, 1x Buffer II (Applied Biosystems, Foster City, CA), 3.25 mM and 1.25 mM of MgCl_2_ for T and B cells, respectively, and equimolar pooled primers based on Biomed-2 primer panels (see S1 File) (Metabion, Planegg, Germany).[15] Template input concentration is dependent on the B/T cell ratio of DNA extracted from MNCs and therefore varied in between samples (up to 0.8μg of DNA for IGH respectively up to 1μg of DNA for TRB).

A second amplification (adaptor ligation step) was performed to integrate the indices and the P5 and P7 tails for Illumina sequencing using the Nextera DNA library preparation kit (Illumina, San Diego, CA). Final volume of the 2^nd^ PCR was 50μl and amplification conditions were as follows: 95°C for 10 min, 10 cycles of 95°C for 15 s, 63°C for 30 s and 72°C for 30 s, and 72°C for 5 min. The PCR mixture contained 2.5U of AmpliTaq Gold polymerase, 0.2mM of each dNTP, 1x Buffer II, 2mM of MgCl_2_, 5μl of the first PCR product, and 5μl of each index primer as recommended by Illumina. Final PCR products were purified using Agencourt AMPureXP beads (Beckman Coulter, Brea, CA) in a 1 to 0.95 DNA per bead ratio. The purified amplicons were analyzed by Agilent DNA 1000 (Agilent Technologies, Santa Clara, CA) and normalized using Kapa Library Quantification (KapaBiosystems, Wilmington, MA) as described in the manufacturer’s instructions. Samples were pooled with TE buffer in a final pool concentration of 4nM and diluted to a final concentration of 15pM. The sequencing run was performed in a MiSeq sequencer (Illumina) using the MiSeq Reagent Kit v3 2x300 cycles (Illumina).[7, 9]

Due to the fact that next-generation sequencing is a very sensitive technique it is important to take provisional steps against contamination. Sample preparations for kidney and blood samples were done independently. We also used double indexing for every sample with an index combination that always differed for blood and kidney of the same patient. In addition, we regularly performed wipe tests to confirm DNA free conditions.

### Data analysis

Illumina Miseq output files (paired-end fastq files without Nextera adaptors) were imported into the CLC Genomics Workbench (CLC Bio, version 7, Aarhus, Denmark). Base calls with low quality were trimmed and overlapping paired-end sequences were merged using the NGS core tools of CLC Bio. The forward and reverse reads proofed to be highly overlapping in all 5 primer sets (~200bp for IGH Primer Set 1, ~250 Primer Set 2, a full overlap for Primer Set 3 and ~250bp for TRB Primer Set 1 and 2) minimizing the chance of inaccurate merging. Fasta files were exported from CLC software and uploaded to the HighV-QUEST program at the IMGT webpage (http://www.imgt.org/HighV-QUEST) for the analysis of rearranged immunoglobulin and T cell receptor sequences and so as to assign specific V-(D)-J elements to the sequences [16]. IMEX [17] was used to analyze V-(D)-J usage and sequence functionality, to visualize and summarize the number of productive, unproductive and unknown sequences as well as to study clonality through CDR3 sequences and/or V-(D)-J combination. B and T cells and clones of individual B and T cells (clonotypes) are defined due to their unique CDR3 sequence on nucleotide level. In order to increase readability the nucleotide sequence of the CDR3s was translated to amino acid sequences. It is important to note that clonotypes can also be defined according to other sequence based information, i.e. according to the entire receptor sequence or the V-(D)-J element combination.

### Definition of expansion

As characterization of all analyzed clonotypes would go beyond the scope of this essay we were forced to set limitations which define clonotypes as expanded or as normally abundant. We derived cut off values to define clonal expansion based on data derived from four blood samples of healthy individuals. We took five times the average of the clonotypes with the highest abundance (top clonotypes) based on data from the healthy control group. We also addressed the issue of increasing uncertainty at decreasing percent values. This uncertainty is strongly related to the amount of DNA/cell input into PCRs, so low DNA input results in loss of precision for measuring low percent values. Related to our cell input range we defined 1% as the lower limit. We applied these thresholds for both the kidney and blood compartment due to the fact that we had no access to healthy renal tissue. The minimum abundances for clonotypes to be marked as expanded were defined as follows: 5.46% for amplification with IGH Primer Set 1, 1.15% for IGH Primer Set 2, 1% for IGH Primer Set 3, 6.82% for TRB Primer Set 1and 10.73% for TRB Primer Set 2 (see S3 Table). The selected B cell clonotypes shown in the manuscript have been collapsed, choosing always those with the highest percentage among all three IGH primer sets (see S4 Table). Regarding T cells, all expanded clonotypes were selected, since the information given by the two TRB primer sets should be considered additive, not redundant [15].

### Diversity calculation

In order to calculate the diversity we used a specific workflow that comprises several consecutive steps. First we calculated the number of unique clonotypes per sample (*a*) as previously described by our group [17] and then we used these (*a*) values for the calculation of the inverse Simpson’s diversity index, defining *R* as total number of reads of all unique clonotypes and *r*_*i*_ as the number of reads of each unique clonotype. The inverse Simpson’s diversity index was calculated in the following:

$$D_{S}=1/\frac{\sum_{i=1}^{a}r_{i}\left(r_{i}-1\right)}{R\left(R-1\right)}$$

The algorithm to calculate the (*a*) value allows a mathematical estimation for each individual sample concerning the number of reads that include sequencing errors in the CDR3 region. If the raw data included 1000 different CDR3 sequences (clonotypes) and the mathematical model estimated 200 reads to be artificial clonotypes created by sequencing errors, we reduced the number of clonotypes found in a sample down to 800. The values “R” and “r" for the formula are, therefore, affected by this error correction, as the 200 clonotypes with the lowest read amounts are discarded. It is important to note that we do not define specific reads as erroneous; we rather define a number of erroneous reads per raw reads of an individual sample and discard a number of reads based on this information.

Paired two-tailed t-tests were used to compare the unique number of clonotypes of B vs. T cells with a level of significance of 0.05. Independent two-tailed t-tests were performed to compare Simpson’s index values with a level of significance of 0.05. In addition, we performed statistical tests to confirm that our diversity values were not biased by varying numbers of reads of the individual samples (S4 Fig).

### Similarity calculation

Similarities between blood and kidney compartments were calculated with Morisita-Horn (MH) similarity index, comparing all unique numbers of clonotypes (explanation given above) of one compartment with the repertoire of the other for each individual sample. MH was calculated in the following:

$$C_{MH}=\frac{2\sum_{i=1}^{n}x_{ij}x_{ik}}{\left[\left(\sum_{i=1}^{n}x_{ij}^{2}/N_{j}^{2}\right)+\left(\sum_{i=1}^{n}x_{ik}^{2}/N_{k}^{2}\right)\right]N_{j}N_{k}}$$

*x*_*ij*_ and *x*_*ik*_ are the numbers of reads of a specific clonotype *i* in samples *j* (blood) and *k* (kidney), *N*_*j*_ ($\Sigma_{i=1}^{n}x_{ij}$) is the total number of reads in sample *j*; and *N*_*k*_ ($\Sigma_{i=1}^{n}x_{ik}$) is the total number of reads in sample *k*. Independent two-tailed t-tests were performed to compare MH index values with a level of significance of 0.05.

## Results

After isolating B and T lymphocytes from renal tissue samples and peripheral blood, we applied high-throughput sequencing on the rearranged V-(D)-J regions to assess IG and TR clonality and diversity of the B and T cell repertoire in both compartments. The study comprised blood and tissue samples from 10 patients that were nephrectomized for various medical reasons (S1 Table). Within all patients, we identified a total of 613 036 different clonotypes in blood samples (252 443 B cell clonotypes and 360 593 T cell clonotypes) as well as 261 280 distinct clonotypes in the tissue samples (154 670 and 106 610 for B and T cell clonotypes, respectively). Detailed information on each individual measurement can be found in S5 Table.

**Fig 1:**
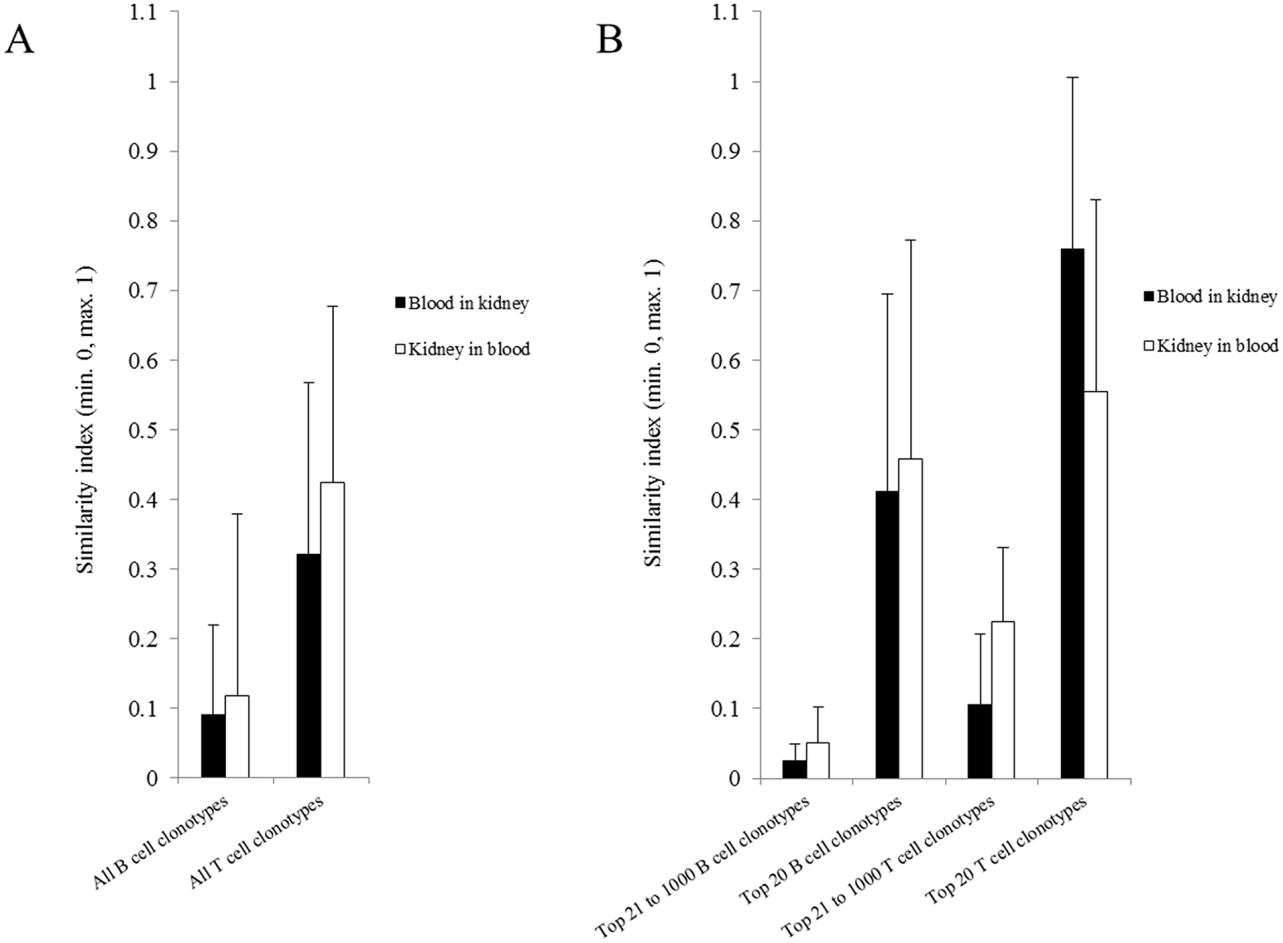
Comparison of Morisita-Horn (MH) similarity index. (A) Comparison of all B and T cell clonotypes after sequencing error correction between compartments in both directions, comparing the clonotypes from the kidney found also in the blood, and those from the blood found also in the kidney. The numbers of clonotypes per compartment that have been included in the analysis are defined by the a values calculated as described in the section diversity calculation (a values from every dataset are listed in S5 Table). In the M-H formula the “n” corresponds to the a value. (B) Comparison of high abundant clonotypes between compartments and comparison of low abundant clonotypes between compartments. High abundant clonotypes are defined as the top 20 and low abundant clonotypes are defined as those in positions from 21 to 1 000. The repertoire comparison was also performed in both directions, from blood to kidney and vice versa. MH can range from 0 (no similarity) to 1 (absolute similarity). Error bars represent the standard deviation between samples of the same group.

In order to assess similarity of the B and T cell repertoire between both compartments, we calculated the Morisita-Horn (MH) similarity index (Fig 1A). Contrasting the presence/absence similarity coefficients (e.g. Jaccard or Sorensen), MH also considers the relative frequencies of the compared clonotypes between samples. Overall, the results show a significantly lower similarity of B cells compared to T cells in both compartments (p<0.05). In depth analysis of the repertoires showed that high abundant clonotypes (top 20) correlate better within compartments compared to low abundant clonotypes (clonotypes from position 21 to 1 000) (Fig 1B). CDR3-based comparisons in both directions differed significantly between high and low abundant clonotypes for B cells (p<0.01) and T cells (p<0.01). The average of MH similarity for clonotypes in blood found in kidney was 0.41 (SD = 0.23) for B cells and 0.56 (SD = 0.15) for T cells in the top 20 clonotypes, while for low abundant clonotypes the average similarity was 0.03 (SD = 0.02) for B cells and 0.11 (SD = 0.09) for T cells. The average of MH similarity for clonotypes in kidney found in blood was 0.46 (SD = 0.31) for B cells and 0.76 (SD = 0.28) for T cells in the top 20 clonotypes, while for low abundant clonotypes the average similarity was 0.05 (SD = 0.05) for B cells and 0.23 (SD = 0.11) for T cells. Individual values for MH similarities are provided in S6 Table.

### Expanded B and T cell clonotypes in kidney and blood compartment

In kidney tissue samples expanded B and T cell clonotypes were found in 8 of 10 patients. Overall, we identified 32 expanded clonotypes in kidney samples (12 T cell clonotypes and 20 B cell clonotypes). In blood samples, expanded clonotypes were found in 6 of 10 patients. We identified a total of 12 expanded clonotypes in blood samples (6 B cell clonotypes and 6 T cell clonotypes). For detailed information on all expanded clonotypes see Table 1 and S4 Table.

**Table 1:** CDR3 sequences, V-(D)-J elements and functionality of highly expanded clonotypes in kidney and blood of all patients.

ID	Indication	Cell Type	Primer set	CDR3	VDJ	Functionality	% Kidney	% Blood	Top 5 in Blood
**Patient 1**	Tumor	B cells	VH-FR2+JH	ARGGGSGSSEVSPGEFDP	IGHV4-34/IGHJ5/IGHD3-10	P	**12,72%**	**7,28%**	✔
			VH-FR1+JH	ARDLGYCSSTSCYCSGGSC*F	IGHV3-30/IGHJ4/IGHD2-2	UP	**10,04%**	4,85%	✔
			VH-FR3+JH	TFVRLEFKLWQTGGRRRTGRNPSAKSSPRRGSGNAWPGQTVL*L	IGHV4-39/IGHJ4/IGHD1/OR15-1a	UP	**7,49%**	0,15%	✖
			VH-FR3+JH	ATSTVTTSAEYFQH	IGHV3-11/IGHJ1/IGHD4-17	P	**2,62%**	**7,01%**	✔
			VH-FR3+JH	ARGLLGGDYGY	IGHV4-34/IGHJ4/IGHD4-17	P	**2,42%**	0,47%	✔
			VH-FR3+JH	VRVQV**KCLPP	IGHV4-34/IGHJ4/IGHD6-25	UP	**1,35%**	0,05%	✖
		T cells	Vß+Jß Set2	ASSYPGMGPQF	TRBV6-2/TRBJ2-1/TRBD1	P	**14,38%**	**28,95%**	✔
			Vß+Jß Set2	ASKVGLADSYNEQF	TRBV6-5/TRBJ2-1/TRBD2	P	**11,36%**	0,06%	✖
			Vß+Jß Set1	ASEGQDNSPLH	TRBV6-1/TRBJ1-6/TRBD1	P	6,02%	**13,34%**	-
**Patient 2**	Tumor	B cells	VH-FR3+JH	ARGKSSYGMDV	IGHV3-11/IGHJ6/IGHD6-19	P	**1,05%**	n.d.	✖
			VH-FR3+JH	ARARQQLNA	IGHV4-30-2/IGHJ5/IGHD6-13	P	0,42%	**1,39%**	-
		T cells	Vß+Jß Set1	ASSYSIFGSQPQH	TRBV6-5/TRBJ1-5/TRBD2	P	**28,04%**	**22,06%**	✔
			Vß+Jß Set1	ASMGGWLH	TRBV6-5/TRBJ1-2/TRBD2	UP	**7,23%**	6,62%	✔
**Patient 3**	Tumor	B cells	VH-FR3+JH	ARLVDYGDYIDY	IGHV4-30-2/IGHJ4/IGHD4-17	P	**1,13%**	<0.01%	✖
		T cells	-	No expansion	-	-	-	-	-
**Patient 4**	Tumor	B cells	-	No expansion	-	-	-	-	-
		T cells	-	No expansion	-	-	-	-	-
**Patient 5**	Contracted	B cells	-	No expansion	-	-	-	-	-
		T cells	-	No expansion	-	-	-	-	-
**Patient 6**	Contracted	B cells	-	Discarded data	-	-	-	-	-
		T cells	Vß+Jß Set2	ASSSVAATSTDTQY	TRBV27/TRBJ2-3/TRBD2	P	**16,69%**	9,80%	✔
			Vß+Jß Set2	ASSLRGPGQAGNEQF	TRBV5-4/TRBJ2-1/TRBD1	P	**13,51%**	**11,21%**	✔
**Patient 7**	Hydronephrotic	B cells	VH-FR1+JH	ASVMGPLLWFGKSQHRYYFDY	IGHV4-34/IGHJ4/IGHD3-10	P	**35,43%**	**6,70%**	✔
		T cells	Vß+Jß Set1	ASSPYTTGRKLF	TRBV12-3/TRBJ1-4/TRBD1	P	**18,62%**	4,47%	✔
			Vß+Jß Set1	ASSKEYRGAGGYT	TRBV6-5/TRBJ1-2/TRBD1	P	**14,52%**	4,80%	✔
			Vß+Jß Set1	ASSPDRGGNQPQH	TRBV18/TRBJ1-5/TRBD1	P	**9,62%**	0,66%	✖
**Patient 8**	Hydronephrotic	B cells	VH-FR2+JH	ALAVSWSRGGDY	IGHV1-69/IGHJ4/IGHD6-6	P	**2,39%**	<0.01%	✖
			VH-FR2+JH	ARRVSSSAADWFDP	IGHV4-30-4/IGHJ5/IGHD6-13	P	**2,12%**	<0.01%	✖
		T cells	Vß+Jß Set1	ASGLSVNQPQH	TRBV12-5/TRBJ1-5/TRBD2	P	**11,11%**	1,46%	✔
**Patient 9**	Hydronephrotic	B cells	VH-FR3+JH	VRERPDGWGNGMDV	IGHV3-53/IGHJ6/IGHD3-10	P	0,76%	**13,71%**	-
			VH-FR2+JH	ARGDSTYNWFDP	IGHV1-2/IGHJ5/IGHD5-24	P	0,05%	**3,98%**	-
			VH-FR3+JH	ARDFRKRCFDI	IGHV3-48/IGHJ3/IGHD4-11	P	**1,11%**	0,15%	✔
		T cells	Vß+Jß Set1	AALARGF	TRBV19/TRBJ1-1/TRBD2	UP	**13,13%**	0,01%	✖
**Patient 10**	Acute rejection	B cells	VH-FR3+JH	ARSPDCGGDCYSGMDV	IGHV4-34/IGHJ6/IGHD2-21	P	**26,57%**	0,01%	✖
			VH-FR3+JH	ARDIAAAGSWGYYYYYGMDV	IGHV3-33/IGHJ6/IGHD6-13	P	**20,35%**	0,01%	✖
			VH-FR3+JH	ARGAIRDGYKPNGTSI	IGHV4-34/IGHJ2/IGHD5-24	UP	**13,40%**	0,01%	✖
			VH-FR1+JH	AETSDTVGVTDAMGWTTGST	IGHV1-24/IGHJ5/IGHD2-2	UP	**5,70%**	<0.01%	✖
			VH-FR3+JH	ATEKWGSFGV	IGHV1-24/IGHJ3/IGHD3-16	P	**3,61%**	<0.01%	✖
			VH-FR2+JH	ARVNGLVRGARGFDY	IGHV3-21/IGHJ4/IGHD3-10	P	**2,49%**	<0.01%	✖
			VH-FR2+JH	**YLLLLC	IGHV7-81/IGHJ4/IGHD3-22	UP	**1,78%**	n.d.	✖
			VH-FR2+JH	FGYYDSSGYYYGEAFDI	IGHV3-7/IGHJ3/IGHD3-22	P	**1,50%**	<0.01%	✖
								**CD8** ^**+**^	-
		T cells	Vß+Jß Set2	ASSWTSGSGNEQF	TRBV27/TRBJ2-1/TRBD2	P	**35,63%**	0.30%	✖
			Vß+Jß Set1	ASSTVGDKDNS	TRBV11-3/TRBJ2-7/TRBD1	UP	2,02%	**9.47%**	-
			Vß+Jß Set2	AISDPTLAGGPEQF	TRBV10-3/TRBJ2-1/TRBD2	P	1.95%	**18.03%**	-

Percent values for expanded clonotypes in kidney and blood samples are highlighted in bold. Expanded clonotypes that could not be detected in the other compartment were marked as not detected, n.d. Clonotypes are also categorized according to primer sets regarding Biomed-2 primer panel [15]. B cell results from patient 6 were discarded due to low amount of DNA input leading to inaccurate clonotype proportions. T cell results from blood sample of patient 10 were sorted in CD4^+^/CD8^-^ and CD4^-^/CD8^+^ subpopulations. The T helper cell subpopulation showed no expansions and is therefore not listed (we found a very low abundance of the expanded clonotype in the kidney in this sample and we assume that these are traces from the CD4^-^/CD8^+^ population and therefore this sample was discarded). Functionality of the rearranged chain is marked P for productive and UP for unproductive.

According to our data clonal expansion of B and T cells is linked. In 8 out of 9 patients (patient 6 was excluded from this analysis because B cell data was discarded) clonal expansion appeared either in both cell types or in none. All, except two clonotypes that were expanded in the kidney (93.8%) were also found in the blood samples (12 of 12 T cell clonotypes and 18 of 20 B cell clonotypes). However, not all of these clonotypes showed equally high abundance in systemic circulation. Only 6 T cell clonotypes and 6 B cell clonotypes fulfilled the defined criteria for clonal expansion in the blood samples. Similarly, all expanded clonotypes in the blood could also be traced in the kidneys. Overall, 3 B cell clonotypes and 3 T cell clonotypes that were expanded in the blood also showed clonal expansion in the kidney.

### V-(D)-J element distribution of expanded B and T cell clonotypes

**Fig 2:**
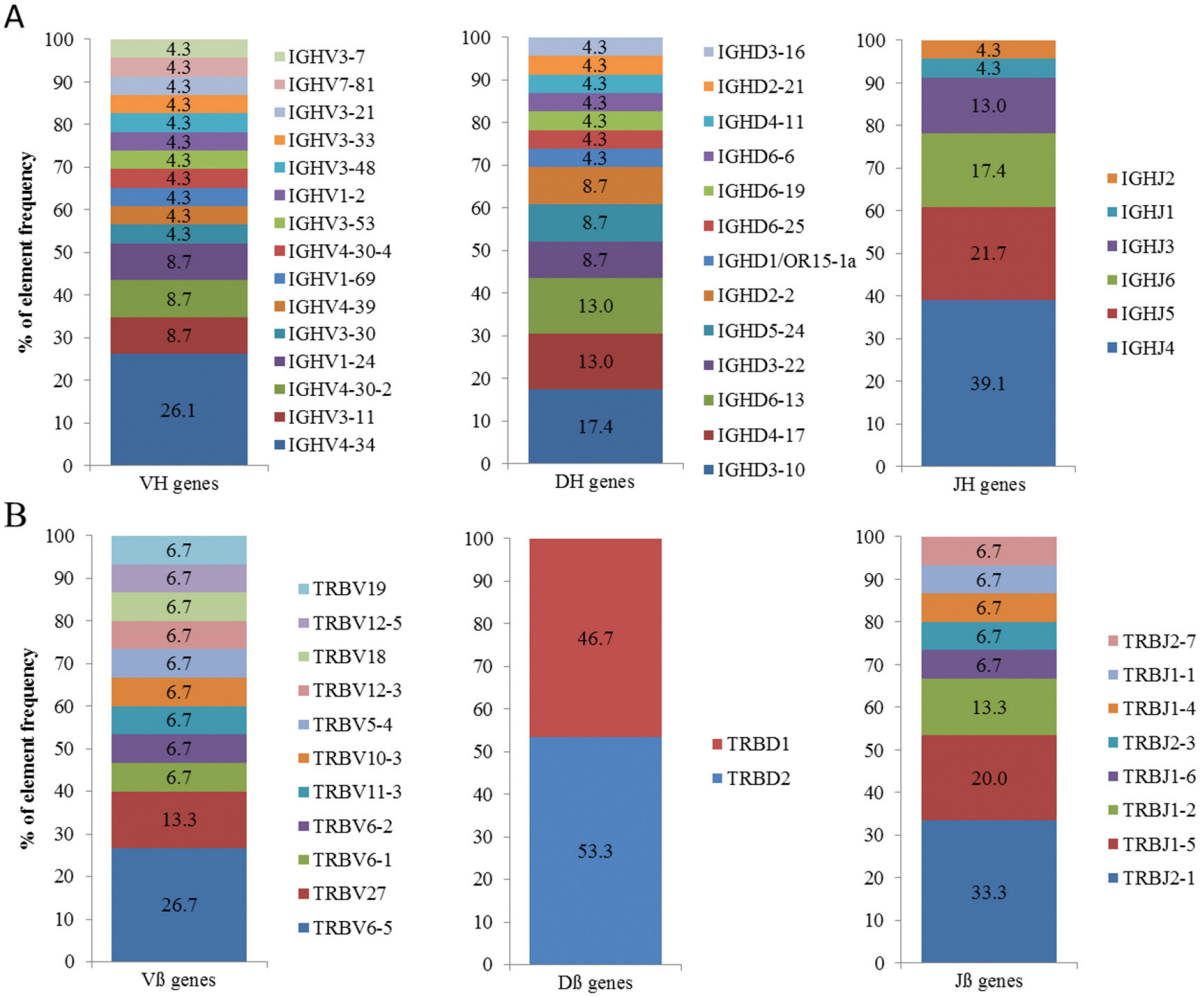
Quantification of element frequency (%) in highly expanded clonotypes. (A) VH, DH and JH element distribution of expanded B cell clonotypes and (B) Vß, Dß and Jß element distribution of expanded T cell clonotypes. We would appreciate the creation of a publicly available database with common V-(D)-J element frequency distribution in human populations to identify potential shifts in the course of diseases as to our knowledge this has not been provided to the community until this date.

An analysis of V-(D)-J element distribution of the immunoglobulin heavy chain (IGH) in highly expanded B cell clonotypes reveals a predominance of certain elements: IGHV4-34 (26.1%), IGHD3-10 (17.4%), IGHD4-17 (13.0%), IGHD6-13 (13.0%), IGHJ4 (39.1%) and IGHJ5 (21.7%) (Fig 2a). For T cells the distribution is similar: TRBV6-5 (26.7%), TRBD1 (46.7%), TRBD2 (53.3%), TRBJ2-1 (33.3%) and TRBJ1-5a (20.0%) (Fig 2b). Our data showed predominance of specific elements, irrespective of the underlying disease. Contrasted with our healthy population element distribution (See S5 and S6 Figs), the above mentioned predominant V, D and J elements in highly expanded B cell clonotypes always appear within the top-10 most common elements in healthy individuals. Moreover, all IGHJ elements were found in at least one expanded clonotype. For T cells, TRBV6-5 was found in healthy individuals representing an 8.9% of the complete repertoire, being the highest represented element on the healthy population. TRBJ element frequencies of expanded clonotypes cannot be compared directly with the data obtained from healthy individuals due to the limitations given by the BIOMED-2 primer settings, where different J elements are targeted in different PCR reactions and, therefore, final percentages do not represent the entire repertoire. In order to determine potential predominance of specific V, D and J elements in the course of certain diseases large cohorts of both, healthy and diseased individuals, should be analyzed and compared. In this study the identified expanded clonotypes showed the same element usage as healthy probands which allows us to assume that no disease specific preferential selection of certain elements occurred. Concluding, our data confirms the findings of previous studies that the frequencies of V, D and J element usage in human populations are not equally distributed but rather specific. [18, 19]

### Lymphocyte subpopulations in kidney and peripheral blood

**Fig 3:**
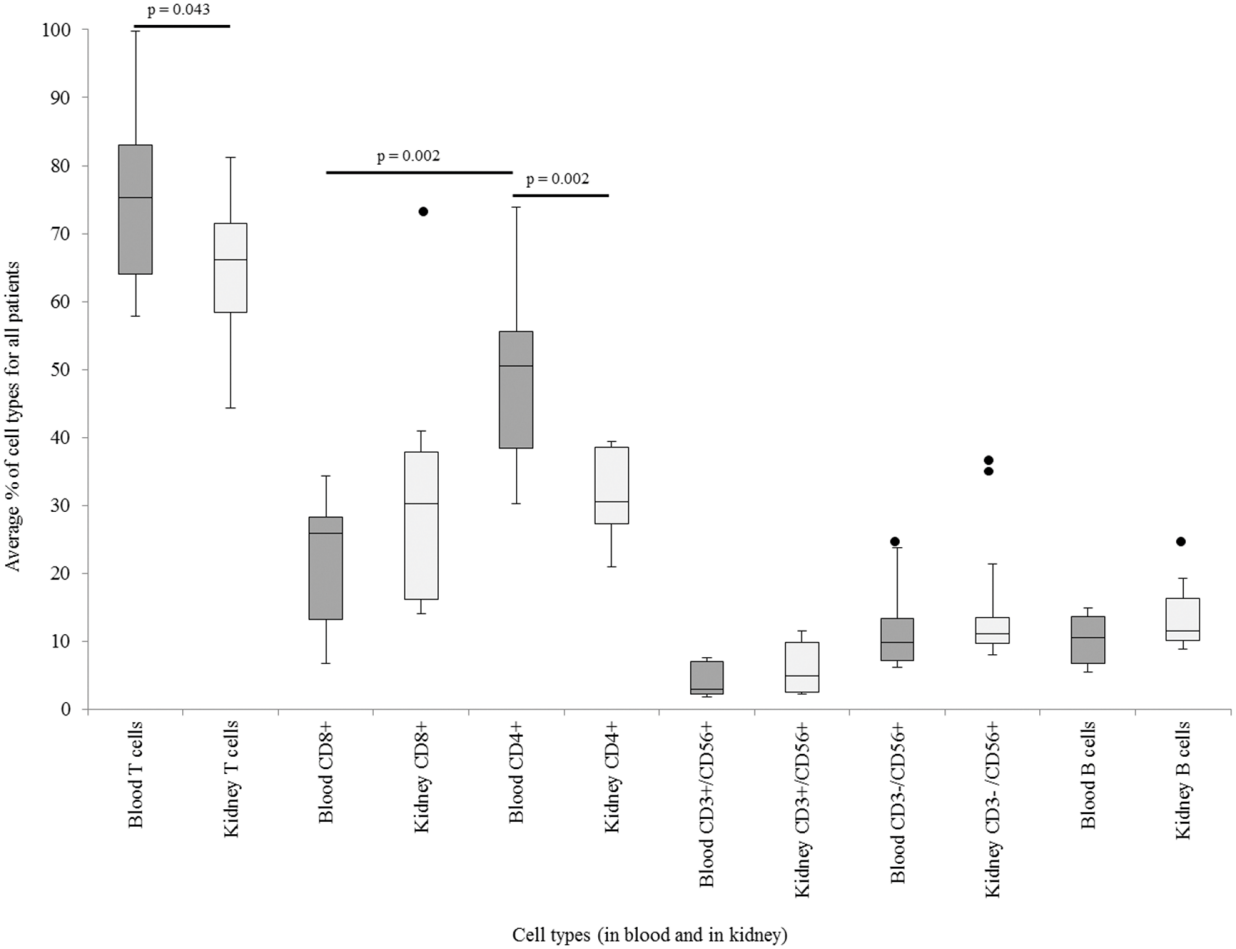
Average percentage of blood and kidney lymphocyte subpopulations in all patients. T cells were separately analyzed for CD4^+^ and CD8^+^ subpopulations. NKT cells were gated using CD3^+^/CD56^+^ and NK cells, by CD3^-^/CD56^+^. Cells presenting CD19^+^ and/or CD20^+^ surface markers were categorized as B cells. Significant differences were marked by a connecting line and the corresponding P-value (paired two-sided t-test).

In the blood compartment, T cells accounted for 74.7% (SD = 10.5) of all lymphocytes, with a significant predominance of CD4^+^ clones (48.8%, SD = 12.6)) compared to CD8^+^ (22.2%, SD = 9.4) (p<0.01). The overall B cell proportion of lymphocytes was 11.6% (SD = 5.9) and in Natural killer (NK) cells represented 11.5% (SD = 7.0) in blood, while natural killer T cells (NKT) comprised 5.1% (SD = 4.5). Therefore, the lymphocyte subpopulations are consistent with common values for peripheral blood lymphocyte subpopulations [20]. In kidney samples, the average for T cells was 65.5% (SD = 13.7) (31.7% for CD8^+^, SD = 18.0, and 31.1% for CD4^+^, SD = 10.1, with no significant differences; p = 0.94), which is significantly different from the T cells in the blood (p = 0.04). B cell proportions represented a 12.8% (SD = 6.8). NKT cells accounted for 6.6% (SD = 5.2) and NK cells for 16.3% (SD = 12.0). A comparison of CD4^+^ population in blood and in renal tissue showed significantly lower percentages in the kidney samples (p = 0.002, 48.8% in blood and 31.1% in kidney) (Fig 3). NK and NKT cells were added to the FACS analyses to attain a holistic picture of the lymphocytes subpopulations. In addition recent studies pointed out that the field of kidney transplantation could profit from including NK cells to immune monitoring analyses.[21] Details are shown in S7 Table.

### Diversity

**Fig 4:**
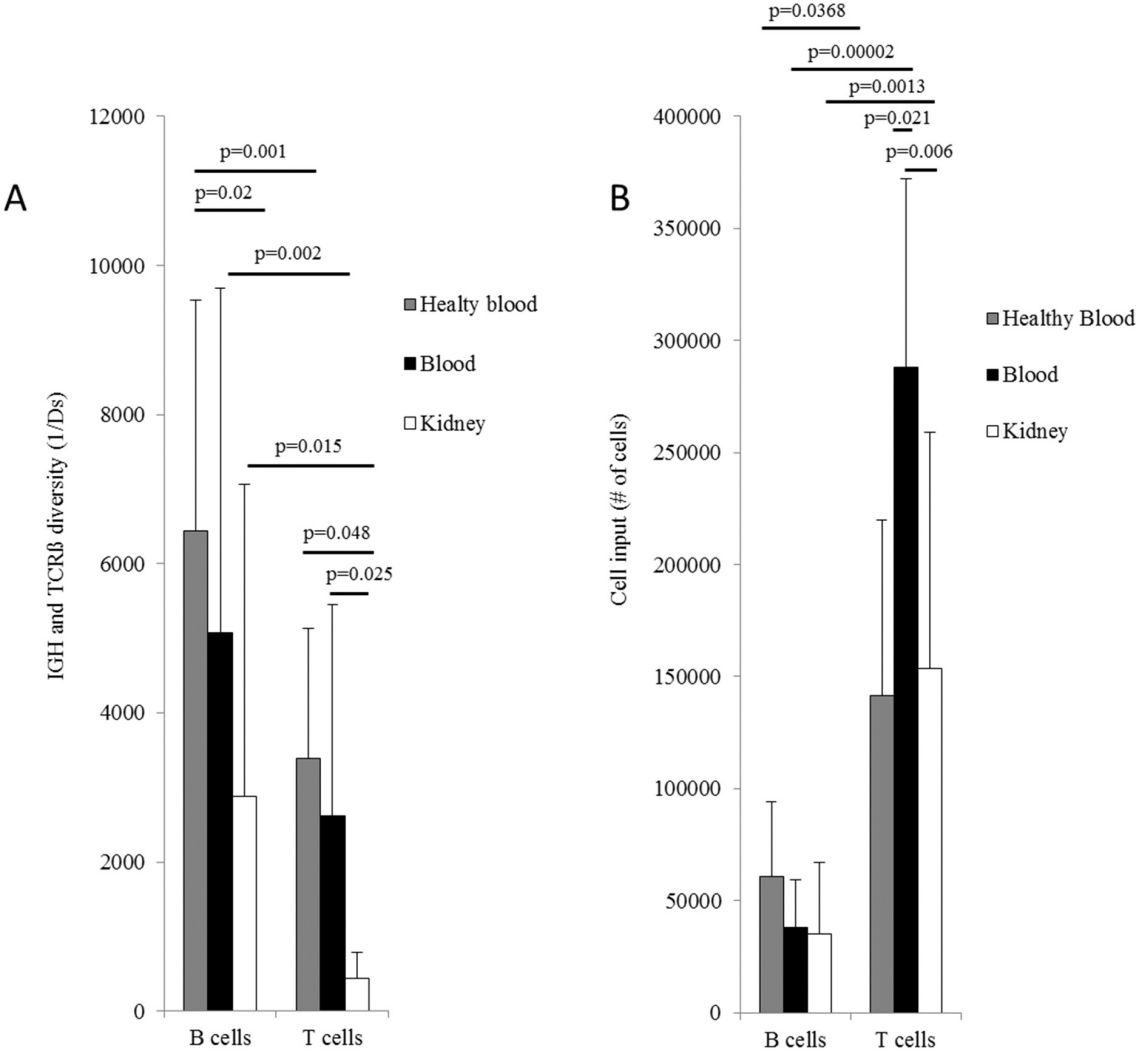
Inverse Simpson’s diversity index and cell input. (a) IGH and TRB diversity of the average of all patients and healthy volunteers based on the inverse Simpson’s diversity index. Independent two-sided t test were performed with a level of significance of 0.05. (b) Average cell input of all patients and healthy volunteers for B and T cells. Paired two-sided t test were performed with a level of significance of 0.05. Error bars represent the standard deviation between samples of the same group.

B and T cell repertoire diversities of the tested samples were significantly dissimilar. The mean diversity values of all B cells in blood (1/*D*_s_ = 5 069.8, SD = 4 633.1) was significantly higher (p = 0.002) than the mean diversity values of T cells in blood (1/*D*_s_ = 2 624.7, SD = 2 834.8). B and T cells derived from kidneys showed an even greater difference in diversity with a 6.5-fold higher diversity value for B cells compared to T cells (kidney derived T cells 1/*D*_s_ = 441.6, SD = 352.8, kidney derived B cells 1/*D*_s_ = 2 876.4, SD = 4 188.2; p = 0.016). Comparing the two compartments, T cell diversity is significantly lower in the kidney than in the blood (p = 0.025) (Fig 4a). Contrasting the latter, healthy volunteers displayed a diversity of 1/*D*_s_ = 6 445.4 for blood derived B cells (SD = 3 095.8) and of 1/*D*_s_ = 1 628.3 for blood derived T cells (SD = 1 741.0). There were no significant differences between the healthy volunteers’ blood diversity and the patient’s blood diversity in both, B cells (p = 0.32) and T cells (p = 0.65). However, significant differences between the B cells (p = 0.03) and the T cells (p = 0.048) of the diseased renal tissue compared to blood from healthy volunteers could be found. Our results show that, the comparison between B and T cells for all healthy volunteers also showed significant differences (p = 0.001). Further details on diversity calculation are given in S8 Table.

In order to confirm whether the differences in the observed diversity values were influenced by the cell input we calculated the average cell input of all patients and healthy volunteers for B and T cells in blood and kidney (S9 Table). Results proof, that, as expected, in blood and kidney T cells were significantly more abundant than B cells (T cells in blood = 287 816, SD = 84 402.3; B cells in blood = 38 081, SD = 21 475, p = 0.00002; T cells in kidney = 153 648, SD = 105 538, B cells in kidney = 35 226, SD = 32 031, p = 0.001). Similarly, T cells showed significant differences between compartments (p = 0.006) (Fig 4b). On average, the healthy volunteers’ cell input was significantly different (141 432 for T cells, SD = 78 651.3, and 60 613 for B cells, SD = 33 707.5; p = 0.037). A comparison of cells derived from healthy individuals’ blood with cells derived from patients’ blood and kidney showed significant differences only in the T cell input between healthy individuals’ blood and patients’ blood, although this could be explained as a result of the small healthy cohort (healthy individuals’ blood vs. patients’ blood B cells input p = 0.29; healthy individuals’ blood vs. patients’ kidney B cells input p = 0.20; healthy individuals’ blood vs. patients’ blood T cells input p = 0.02; and healthy individuals’ blood vs. patients’ kidney T cells input p = 0.82). We found higher diversity values for B cells even if our analysis contained significantly more T cells.

## Discussion

Our next-generation sequencing (NGS) data of IG and TR repertoires from patients with various kidney diseases confirmed that clonal expansion of renal infiltrating T and B cells is a common phenomenon [4]. Most of these expanded clonotypes can also be traced in peripheral blood samples of these patients, albeit not all of them showed an equally high abundance in both compartments. To a certain degree these findings contradict former studies were it was not possible to find the same clonotypes in blood and kidney [4] or other diseased tissue [22–24].

In addition, our data suggest that clonal expansions of T and B cells are correlating (in 8 out of 9 patients), a fact not described in previous studies. Once locally highly abundant B and T cell clonotypes have been identified, immune repertoire analysis of lymphocytes from peripheral blood samples may facilitate monitoring disease progression or response to therapy.[25]

In the absence of lymphoma, highly expanded B and T cells indicate the presence of a locally expressed antigen that drives proliferation and clonal expansion. The highly expanded clonotypes in our samples represented up to 30% of all B and T cells in the respective sample suggesting a strong antigenic stimulus for proliferation. Interestingly some of the high abundant clonotypes shown in Table 1 appear to have unproductive IGH or TRB chains which seems impossible at the first glance. There are several explanations for that phenomenon, however. One option is that certain highly expanded B or T cell clonotypes are biallelic clonotypes and are, therefore, carriers of an unsuccessful rearrangement on the first allele, followed by a successful one at the second [26]. This automatically leads to a parallel abundance of a productive and unproductive chain on gDNA level. Another source for highly abundant unproductive chains can be PCR crossover products [27] or alignment errors from the IMGT database.

We also sequenced blood samples from healthy individuals that did not show any signs of clonal expansions in order to have a baseline for definition of expansion. The definition of thresholds allows to condense the amount of clonotypes and to facilitate the tracking of the predominant clonotypes in both compartments. Since there are no previous definitions of expansion in literature [8, 28], we applied 5 times the average of the highest clonotype of different healthy donors for each independent primer set (S3 Table). This was a very conservative definition of expansion so we are confident that a clonotype, that we marked as expanded, is indeed exceeding frequencies which can appear in healthy individuals (S7 Fig). Nevertheless, the definition of thresholds might benefit from large cohorts and less heterogeneous samples. Even so, the definition of natural occurring fluctuations of clonotype frequencies, which frequencies can still be considered as healthy and where pathological abundances start is still challenging and must be addressed in future work.

A general limitation of immune repertoire profiling is the incapability to define the antigen specificity of an expanded clonotype by analyzing the receptor sequence. Even by the help of state of the art technology it is impossible to confirm that the expansion of tissue infiltrating lymphocytes is caused by the interaction of a kidney disease related antigen with specific IG and TR, as no antigen specificity can be attributed to an individual sequence. However, it seems plausible that clonotypes that are highly expanded in the kidney and have lower abundances in the blood derive from clonal expansions stimulated by local antigens. Indeed this was the case for the majority of expanded clonotypes. Nevertheless, we also found 8 expanded clonotypes with higher relative abundances in blood than in kidney. In this case the possibility that the driving force for clonal expansion might not be related to local antigen stimulus in the kidney cannot be dismissed. [29, 30]

By applying Morisita-Horn similarity index on both compartments for high and low abundant clonotypes the data indicates that there is significantly higher similarity in-between compartments for high abundant clonotypes than for low abundant clonotypes. On the one hand this could be explained due to an increase of observational errors in the course of decreasing abundances but on the other hand this could also be a specific phenomenon related to different homing and migration behavior between activated and non- activated cells [31, 32]. Additional investigation using NGS based immune repertoire analysis focusing on homing and migration of expanded B and T cells including B and T cell subgroups could be beneficial to achieve sufficient insights.

Patients included in our study were nephrectomized due to four different renal diseases (tumor, contracted kidney, hydronephrosis and acute allograft rejection). Despite the low sample size, we found some idiosyncrasies in the different patient groups that have to be confirmed in larger cohorts. For tumor and contracted kidneys B and T cell expansions varied and compiled a heterogeneous picture. The patient group suffering from hydronephrotic kidneys showed high T cell clonality in all kidney samples. We could also find B cell expansions in all three patients including patient 9, who had higher expansions in blood than in kidney samples. In addition to the small sample size, heterogeneity of biopsy areas is also likely to contribute to inconsistencies in the results.

Finally, we analyzed data of one patient with the diagnosis of acute renal allograft rejection. The blood derived T cells of this patient were split into cytotoxic T cells and T helper cells for gaining additional information about the specificities of the expanded clonotypes in the kidney. Overall 59.7% of MNCs derived from kidney counted for T cells in this patient, compared to an average of 37.1% in diseased kidneys in general. Also a much higher percentage of cytotoxic T cells (49.5% in the patient compared to 17.9% on average) was found. Based on our criteria we found 3 highly abundant T cell clonotypes in this patient, 2 in the blood and 1 in the kidney. All three clonotypes could be identified as CD8+ T cells. As we did not sort the kidney derived T cells we had to identify the specificity of the kidney derived expanded T cell clonotype indirectly by checking the specificity of this clonotype in blood. In this patient we found very low B cell diversity values that are most probably present due to low proportions of B cells in the kidney (0.85%). Nevertheless abundance frequencies of high abundant clonotypes are not affected by low cell input next to a potentially higher observational error [33]. However, the three independent IGH amplifications showed comparable values and therefore proved that the increase of observational error is negligible in this case. (Clonotype frequencies of the three independent IGH amplifications are shown in S4 Table)

It should be noted that even though immune repertoire profiling was revolutionized by NGS. Existing restrictions such as sequencing errors (related to the technology) and PCR bias and limitations by sensitivity can only be partly overcome and depend heavily on the used approach [10, 34, 35]. For this study we decided to utilize a genomic DNA-based amplification design, combined with high sample input to facilitate estimation of the clonality of a given TRB or IGH sequence by applying a well-established multiplex primer approach [9]. Using genomic DNA in contrast to mRNA as a starting template has the advantage of superior sensitivity [36] including the drawback that recently developed strategies to reduce the multiplex PCR bias are only available for cDNA approaches [34, 37]. As shown in this study, harvesting B and T cells from a large biopsy of the kidney in accordance with a highly sensitive genomic DNA amplification enables the detection of expanded clones in tissue. Surprisingly, these clonotypes can be confirmed in peripheral blood. The used set-up is possibly the reason why our data cannot be easily compared to the work already undertaken. Cheng and coworkers [4] demonstrated that expanded clones in the kidney are detectable but they were unable to track these CDR3 sequences in the blood most probably due to a reduced sensitivity based on small cylinder biopsies or cDNA amplification. Moreover, additional studies using other diseased tissues [22–24] have also failed to provide an orienting contiguity.

In recent years several reports focusing on genomic DNA based immune repertoire profiling [9, 36, 38, 39] have confirmed the usability of next generation sequencing; however method reproducibility, as shown in S8 Fig, is a fundamental requirement to produce reliable data and should always be examined individually. Moreover, concordance of the three independent IGH amplifications of all predominant B cell clonotypes was tested to provide additional validation of the data (S3 Table).

It is particularly challenging to determine diversity values, i.e. the broadness of the immune repertoire as it requires extrapolation from a small sample size to bigger ones, e.g., from a 10ml blood tube to the whole individual.[40, 41] In order to simplify the diversity analysis, we decided to define the diversity only for the sample and not for the patient. Although our analysis contained significantly more T cells, we still found higher diversity values for B cells. Taking into account these differences in cell numbers we can assume that the results for diversity are underrepresenting B cell diversity in relation to T cells. This reflects our expectations as the additional diversification process of somatic hypermutation and affinity maturation is restricted to B cells. Moreover, we could observe higher diversity in the blood than in the kidney for T cells as well as for B cells. This was not surprising either, as cells that infiltrate the kidney tissue should be only a small fraction of the whole repertoire that specifically interacts with renal tissue related antigens.

The human immune system permanently interacts with pathogens, and some pathogens might trigger detectable clonal proliferation even if the person can be considered healthy. Therefore, defining a repertoire as healthy or diseased requires the definition of reliable cut-offs that further require analysis of huge cohorts of healthy volunteers.

In summary, with this study we were able to demonstrate that B and T cells that show clonal expansion in kidneys can also be traced in blood. We encourage the community to expand investigations in this field by using less heterogeneous and larger sample sizes as the results are very promising with regard to the application of Immune repertoire profiling in combination with FACS as a potential blood based biomarker.

## Supporting Information

S1 Fig Gating strategy for T cell subpopulations. CD45+CD3+ T cells were gated using Horizon V500-A and PE-A antibodies after standard lymphocyte FSC vs. SSC (or volume vs. internal complexity) sorting. CD8+ and CD4+ were sorted using FITC-A and APC-A, respectively. This strategy was used for MNCs derived from blood and kidney.
(DOCX)

S2 Fig Gating strategy for analysis of NK and NKT cells. CD45+CD3+ T cells were gated using Horizon V500-A and APC-A antibodies after standard lymphocyte FSC vs. SSC (or volume vs. internal complexity) sorting. CD3-CD56+ (NK) and CD3+CD56+ (NKT) were sorted using PE-A for CD56+ vs. APC-A for CD3+. This strategy was used for MNCs derived from blood and kidney. B cells (CD19+CD3-CD56-) were additionally gated to corroborate the results from the CD3 vs. CD56 comparison.
(DOCX)

S3 Fig Gating strategy for the analysis of B cells (CD19+ and/or CD20+). CD45+ lymphocytes were gated using Horizon V500-A after standard lymphocyte FSC vs. SSC (or volume vs. internal complexity) sorting. CD19+ and CD20+ were sorted using APC-A and FITC-A, respectively. We considered the condition CD19+ and/or CD20+ to count all possible B cells subtypes (CD19+/CD20-, CD19-/CD20+ and CD19+/CD20+). This strategy was used for MNCs derived from blood and kidney.
(DOCX)

S4 Fig Effects of read counts on the diversity “a” value (number of unique clonotypes). In order to confirm that the number of unique clonotypes (a) of each patient was not biased by the number of reads, we calculated the Pearson’s sample correlation coefficient (r) between reads and unique clonotypes for each independent primer set. The results showed no correlation. Also, a paired two-tailed T test comparison between blood and kidney confirmed that significant differences in unique numbers of clonotypes are independent from read counts.
(DOCX)

S5 Fig Vh, Dh and Jh element distribution (%) in healthy individuals. V, D and J gene elements appear at similar frequencies in the four healthy volunteers (see correlation tables). Only Jh element distribution was dissimilar for healthy 1. Vh elements that were not present in any of the healthy volunteers: V7-NL1, V7-77, V7-56, V7-40D, V7-27, V4-80, V4-30-1, V3-79, V3-76, V3-75, V3-65, V3-60, V3-6, V3-57, V3-50, V3-42D, V3-42, V3-41, V3-37, V3-36, V3-30-5, V1-67, V1-17, V1-14 and V1-12. Dh elements not present: D5-5 and D4-4. Correlation between healthy individuals for V, D and J element distribution was calculated using Pearson’s sample correlation coefficient (r).
(DOCX)

S6 Fig Vβ, Dβ and Jβ element distribution (%) in healthy individuals. V, D and J segments appear at similar frequencies in the four healthy volunteers (see correlation tables). There are no correlation data from Dβ elements because the number of elements is too low (only two). Jβ elements are separated in Vβ-Jβ Set 1 and Vβ-Jβ Set 2 because the BIOMED-2 reverse primers for T cell receptors are additive and therefore it is not possible to sum up frequencies to a single value. Small percentages that correspond to primers not present in the primer set can occur due to element assignment error or cross-contamination (J2-1, J2-5, J2-3 and J2-4 for Vβ-Jβ Set 1, and J2-7. J2-2, J1-3, J2-6, J1-1, J1-2, J1-5, J1-4 and J1-6 for Vβ-Jβ Set 2). Vβ elements not present in any of the healthy volunteers: V8-2, V8-1, V7-5, V5-2, V22-1, V17, V1, V23, V21, V16 and V12-2. Correlation between healthy individuals for V, D and J element distribution was calculated using Pearson’s sample correlation coefficient (r).
(DOCX)

S7 Fig Top 20 highest expanded clonotypes for blood and kidney in all patients and for blood in healthy individuals. The histograms represent the abundance (in %, Y-axis) of each clonotype (CDR3-based, X-axis) and the red line represents the assigned thresholds for IGH primer sets 1, 2 and 3 and TRB primer sets 1 and 2.
(DOCX)

S8 Fig Technical replicates. In the experiment design for three replicates with B and T cell expansions, we compared not only if there is a difference between replicates due to PCR or hands-on errors, but also if the sequencing run can bias the reproducibility of the protocol. Pearson’s sample correlation coefficient for each primer set displays high correlation values between replicates in both, B and T cells, with no remarkable differences between sequencing runs. Only T cell primer set 1 correlation shows lower values for replicate 1 compared to replicates 2 or 3 due to a specific clone dropout in replicate 1 (ASSWSPGGNTIY). The top 10 highest abundant clonotype comparison for each primer set shows the variability of the same CDR3-based clonotype percentage among replicates. Each clonotype is highlighted in one different color for each primer set to compare the concordance in rank position between replicates.
(DOCX)

S1 File Biomed-2 primer panel. The list of nucleotide sequences is provided for each forward and reverse primer for all IGH and TRB primer sets. Also the concentration for each primer in the first amplification is given. The information includes the target sequence in 5’-3’ and the Nextera adaptor sequence in 5’-3’.
(XLSX)

S1 Table Clinical data of patients. The 10 patients included in the study are grouped by age range (using 10-year block groups) and by cause of nephrectomy. The sampled material used for each individual patient is listed for the peripheral blood (ml), kidney tissue (cm x cm) and urine (ml). A histological description of each piece of tissue is also provided.
(DOCX)

S2 Table Antibodies used for FACS. The reaction of lymphocyte subpopulations was evaluated with monoclonal antibodies (mAb) by 4 to 5 color FCM analysis on a FACSAria III (Becton Dickinson). Three FCM tubes were used for each patient and material (Tube 1: CD8-FITC, CD3-PE, CD4-APC, CD45-HorizonV500; Tube 2: CD19-FITC, CD56-PE, CD3-APC-A, CD45-HorizonV500; and Tube 3: CD20-FITC, CD19-APC-A, CD45-HorizonV500). Data was analyzed using FACSDiva software Version 7.0 (BD Biosciences).
(DOCX)

S3 Table Most frequent clonotype from healthy probands. For expansion threshold calculation, we considered the percent values of the most frequent clonotypes in blood of healthy probands for B cell primer sets 1, 2 and 3, and for T cell primer sets 1 and 2. In order to determine representative thresholds, the averages of all healthy probands’ highest clonotypes were calculated for each B and T cell primer set and the resulting value was multiplied by 5. All values above these thresholds were considered expanded in our study. We considered 1% as the lower limit to feel confident about the value, so for B cell primer set 3 we increased the threshold from 0.69% to 1%. The same values were used for blood and kidney (we had no access to healthy renal tissue).
(DOCX)

S4 Table B cell comparison of all primer sets. Comparison of abundance (in %) and rank position of the clonotypes (rank refers to position depending on abundant frequencies) was considered to be expanded in our study for the three B cell primer sets. Collapse of the data for Table 1 was done selecting the highest percentage between primer sets and the same primer set in the other compartment. Expanded clonotypes that could not be detected in the other compartment or primer set were marked as not detected, n.d.
(DOCX)

S5 Table Reads, clonotypes and diversity correction. Individual information is given for all primer sets in blood and kidney on the number of reads, the number of clonotypes from sequencing (CDR3-based), the corrected number of unique clonotypes [17] and the percentage of corrected clonotypes selected from the sequencing output after excluding sequencing errors. ^1^ stands for CD4^+^ sorted cells and ^2^ stands for CD8^+^ sorted cells.
(DOCX)

S6 Table Morisita-Horn similarity index calculation. Individual calculations for all patients (min. = 0; max. = 1) for all versus all CDR3-based clonotype comparisons, high abundant clones (top 20) comparison and low abundant clones (from position 21 to 1000) comparison. Patient 10 displays two values on the TR because the blood sample was sorted in CD4+ and CD8+ subpopulations; IG values for patient 6 were discarded due to low DNA concentration. In blue, average combination of primer sets 1, 2 and 3 for B cells and primer sets 1 and 2 for T cells are given.
(DOCX)

S7 Table Lymphocyte subpopulations counted by FACS (in %). Frequencies of lymphocyte subpopulations (T cells, cytotoxic T cells, helper T cells, NKT cells, NK cells and B cells) are given for every patient in blood and kidney as well as P-values for pairwise comparisons (paired two-sided t-test).
(DOCX)

S8 Table Inverse Simpson’s diversity index. High values represent broader diversity. In order to remove sequencing error derived clonotypes we used the diversity calculation feature of IMEX [17]. Diversity was then calculated for patients’ blood and kidney and for healthy individuals’ blood using the inverse Simpson’s diversity formula described in the material and methods section.
(DOCX)

S9 Table Cell input. (1) divided in T cells (CD4+ and CD8+) and B cells. B cells in patient 6 were discarded due to low input. The cell input was calculated using FACS results percentages (2) Cell input of healthy probands using cell proportions from literature (70% T cells and 30% B cells). (3) P-values for comparisons between cell types and compartments using paired two-sided T-test. The calculation was performed using the following formula: Cell input = [X]μg/ml*FACS*(1000pg/μg)/(6.6pg/cell)*30μl. [X]μg/ml is the O.D. concentration of the extracted DNA from the isolated MNCs. FACS is the percentage of cells from each cell type. 6.6pg/cell is the standard amount of DNA in pg per cell. 30μl is the input volume for 1st PCR amplification.
(DOCX)
