# Supplementary figures and images for: Immune Repertoire Profiling Reveals that Clonally Expanded B and T Cells Infiltrating Diseased Human Kidneys Can Also Be Tracked in Blood
Source: PLoS One. 2015 Nov 23;10(11):e0143125. doi: 10.1371/journal.pone.0143125 (PMC4658119; doi:10.1371/journal.pone.0143125)

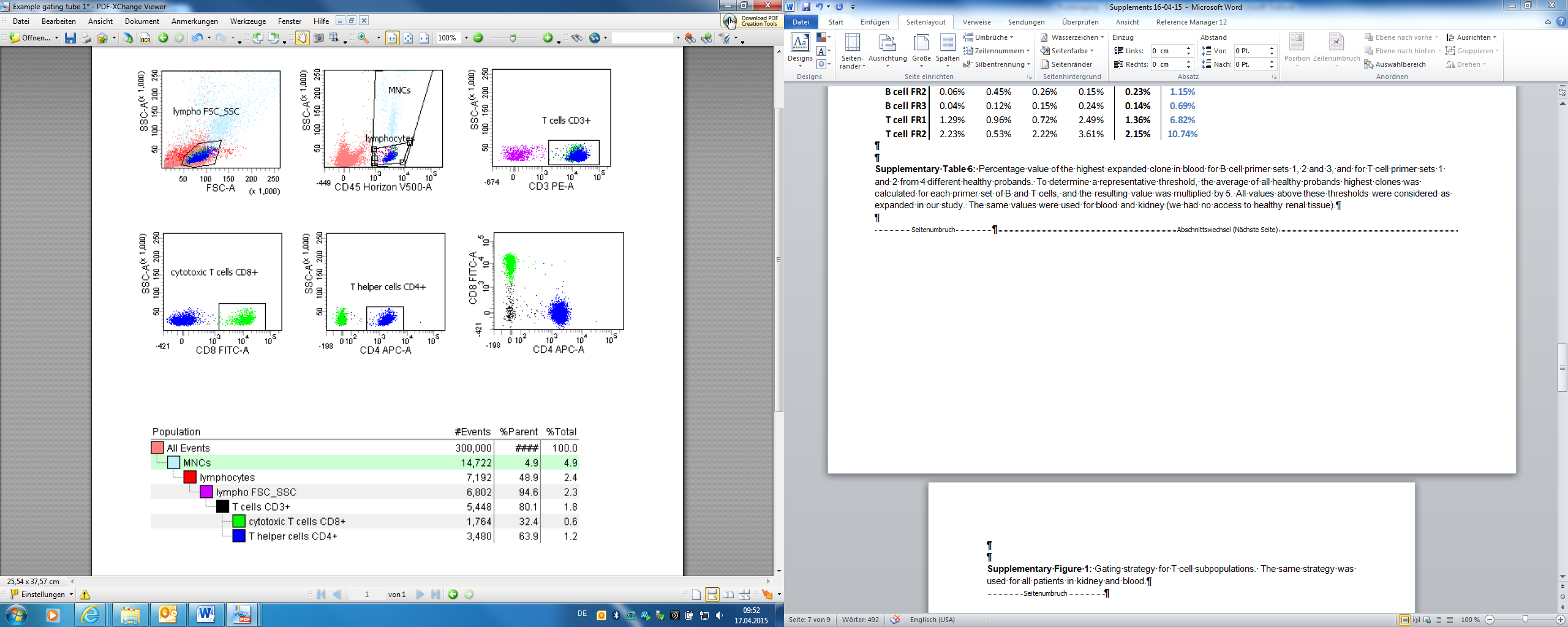
**S1 Fig. Gating strategy for T cell subpopulations.**

Supplement: S1 Fig — CD45+CD3+ T cells were gated using Horizon V500-A and PE-A antibodies after standard lymphocyte FSC vs. SSC (or volume vs. internal complexity) sorting. CD8+ and CD4+ were sorted using FITC-A and APC-A, respectively. This strategy was used for MNCs derived from blood and kidney. (DOCX) [file pone.0143125.s001.docx]

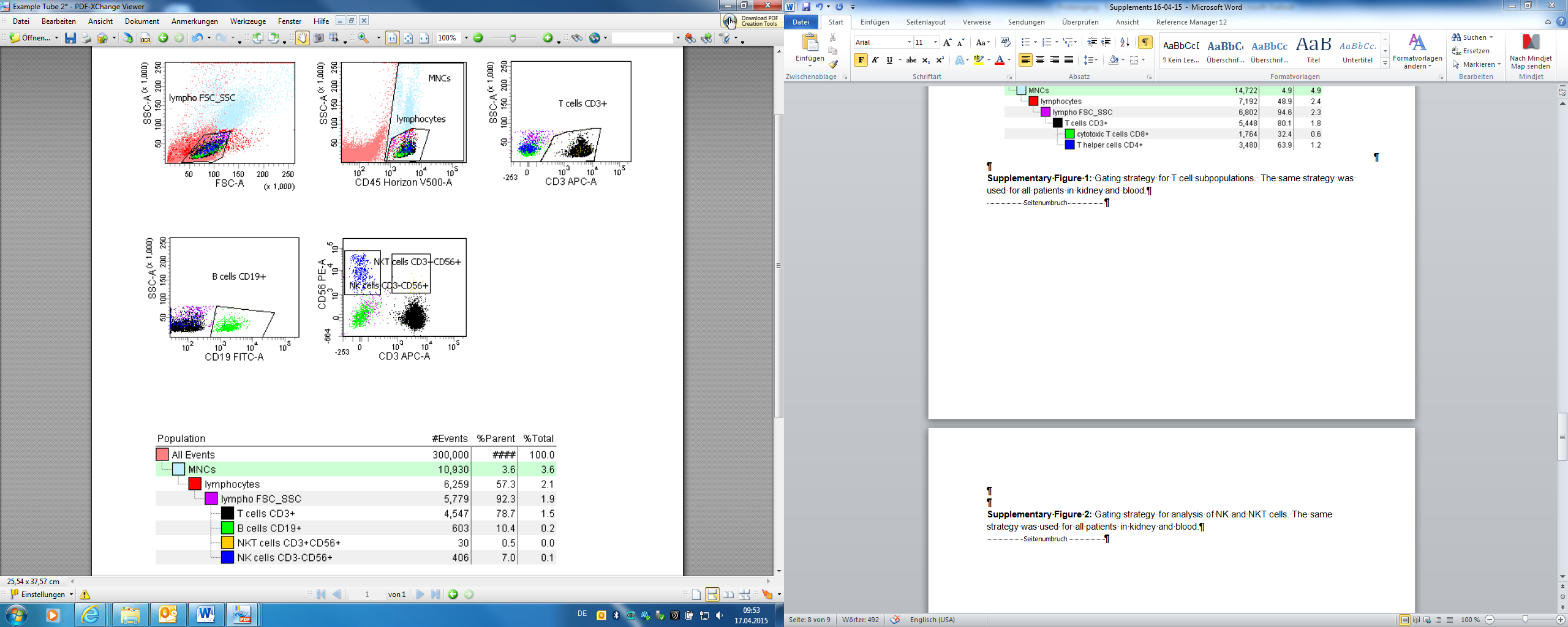
**S2 Fig. Gating strategy for analysis of NK and NKT cells.**

Supplement: S2 Fig — CD45+CD3+ T cells were gated using Horizon V500-A and APC-A antibodies after standard lymphocyte FSC vs. SSC (or volume vs. internal complexity) sorting. CD3-CD56+ (NK) and CD3+CD56+ (NKT) were sorted using PE-A for CD56+ vs. APC-A for CD3+. This strategy was used for MNCs derived from blood and kidney. B cells (CD19+CD3-CD56-) were additionally gated to corroborate the results from the CD3 vs. CD56 comparison. (DOCX) [file pone.0143125.s002.docx]

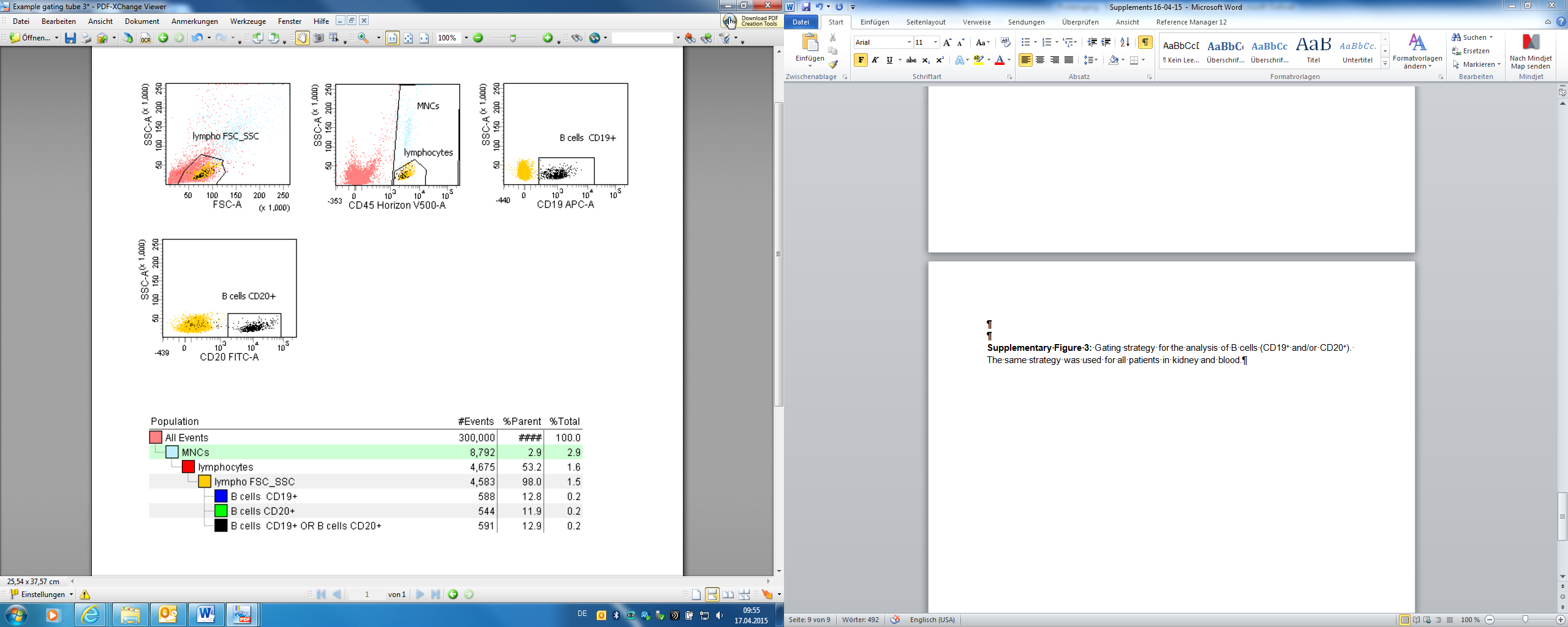
**S3 Fig. Gating strategy for the analysis of B cells (CD19+ and/or CD20+).**

Supplement: S3 Fig — CD45+ lymphocytes were gated using Horizon V500-A after standard lymphocyte FSC vs. SSC (or volume vs. internal complexity) sorting. CD19+ and CD20+ were sorted using APC-A and FITC-A, respectively. We considered the condition CD19+ and/or CD20+ to count all possible B cells subtypes (CD19+/CD20-, CD19-/CD20+ and CD19+/CD20+). This strategy was used for MNCs derived from blood and kidney. (DOCX) [file pone.0143125.s003.docx]

**S5 Fig. Vh, Dh and Jh element distribution (%) in healthy individuals.**


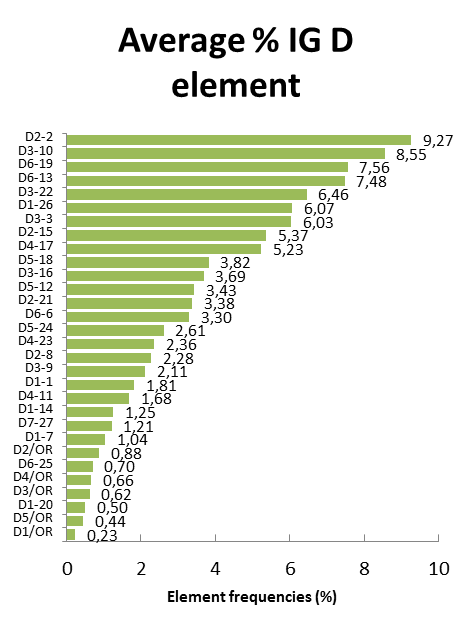

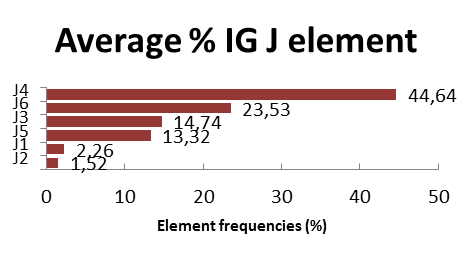

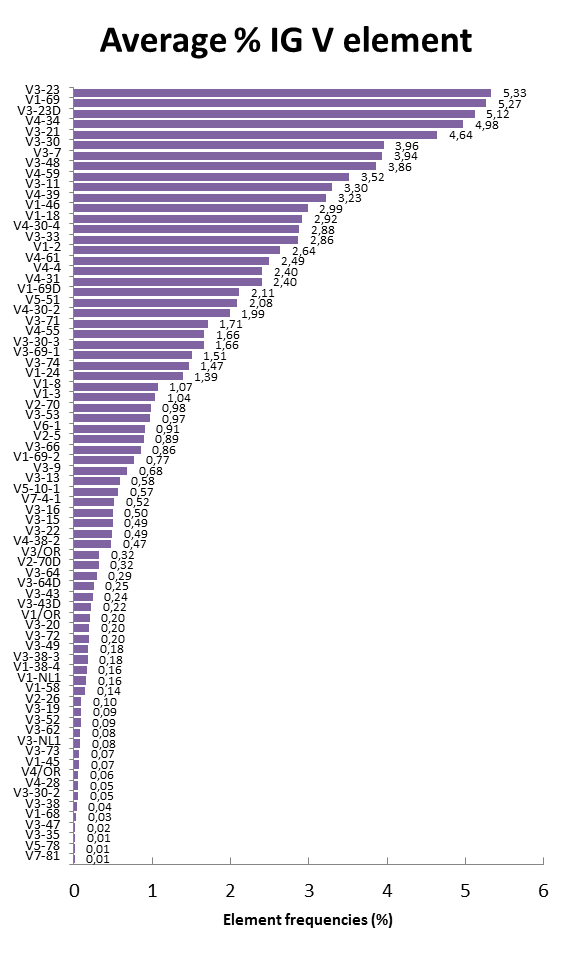

Supplement: S5 Fig — V, D and J gene elements appear at similar frequencies in the four healthy volunteers (see correlation tables). Only Jh element distribution was dissimilar for healthy 1. Vh elements that were not present in any of the healthy volunteers: V7-NL1, V7-77, V7-56, V7-40D, V7-27, V4-80, V4-30-1, V3-79, V3-76, V3-75, V3-65, V3-60, V3-6, V3-57, V3-50, V3-42D, V3-42, V3-41, V3-37, V3-36, V3-30-5, V1-67, V1-17, V1-14 and V1-12. Dh elements not present: D5-5 and D4-4. Correlation between healthy individuals for V, D and J element distribution was calculated using Pearson’s sample correlation coefficient (r). (DOCX) [file pone.0143125.s005.docx]

**S6 Fig. Vβ, Dβ and Jβ element distribution (%) in healthy individuals.**


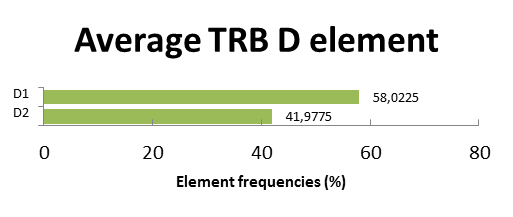

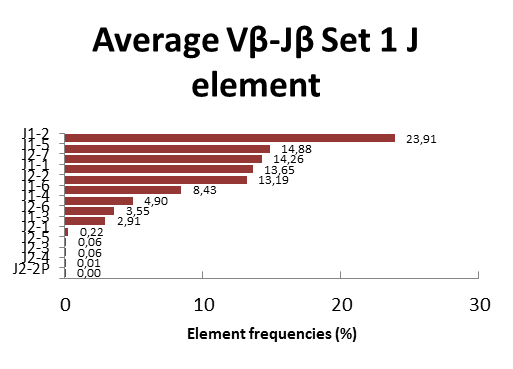

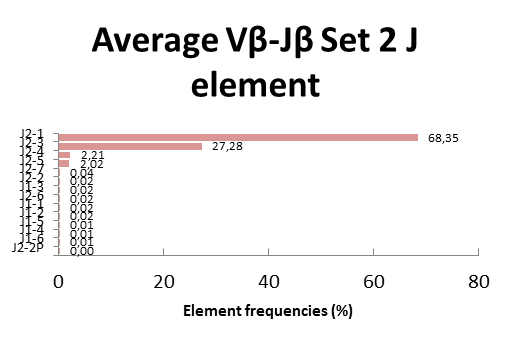

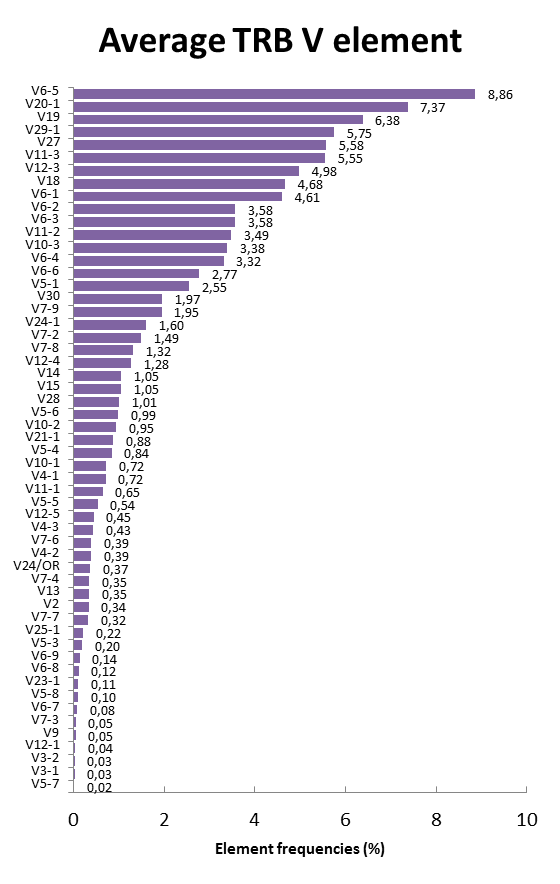

Supplement: S6 Fig — V, D and J segments appear at similar frequencies in the four healthy volunteers (see correlation tables). There are no correlation data from Dβ elements because the number of elements is too low (only two). Jβ elements are separated in Vβ-Jβ Set 1 and Vβ-Jβ Set 2 because the BIOMED-2 reverse primers for T cell receptors are additive and therefore it is not possible to sum up frequencies to a single value. Small percentages that correspond to primers not present in the primer set can occur due to element assignment error or cross-contamination (J2-1, J2-5, J2-3 and J2-4 for Vβ-Jβ Set 1, and J2-7. J2-2, J1-3, J2-6, J1-1, J1-2, J1-5, J1-4 and J1-6 for Vβ-Jβ Set 2). Vβ elements not present in any of the healthy volunteers: V8-2, V8-1, V7-5, V5-2, V22-1, V17, V1, V23, V21, V16 and V12-2. Correlation between healthy individuals for V, D and J element distribution was calculated using Pearson’s sample correlation coefficient (r). (DOCX) [file pone.0143125.s006.docx]
