# Supplementary material for: Immune Repertoire Profiling Reveals that Clonally Expanded B and T Cells Infiltrating Diseased Human Kidneys Can Also Be Tracked in Blood
Source: PLoS One. 2015 Nov 23;10(11):e0143125. doi: 10.1371/journal.pone.0143125 (PMC4658119; doi:10.1371/journal.pone.0143125)
Supplement: S4 Fig — In order to confirm that the number of unique clonotypes (a) of each patient was not biased by the number of reads, we calculated the Pearson’s sample correlation coefficient (r) between reads and unique clonotypes for each independent primer set. The results showed no correlation. Also, a paired two-tailed T test comparison between blood and kidney confirmed that significant differences in unique numbers of clonotypes are independent from read counts. (DOCX) [file pone.0143125.s004.docx]

**S4 Fig. Effects of read counts on the diversity “a” value (number of unique clonotypes).**
