# Supplementary material for: Immune Repertoire Profiling Reveals that Clonally Expanded B and T Cells Infiltrating Diseased Human Kidneys Can Also Be Tracked in Blood
Source: PLoS One. 2015 Nov 23;10(11):e0143125. doi: 10.1371/journal.pone.0143125 (PMC4658119; doi:10.1371/journal.pone.0143125)
Supplement: S7 Fig — The histograms represent the abundance (in %, Y-axis) of each clonotype (CDR3-based, X-axis) and the red line represents the assigned thresholds for IGH primer sets 1, 2 and 3 and TRB primer sets 1 and 2. (DOCX) [file pone.0143125.s007.docx]

**S7 Fig. Top 20 highest expanded clonotypes for blood and kidney in all patients and for blood in healthy individuals.**

Healthy proband Top20 CDR3 clones distribution for IGH primer set 1, 2 and 3, TRB primer set 1 and 2 in blood


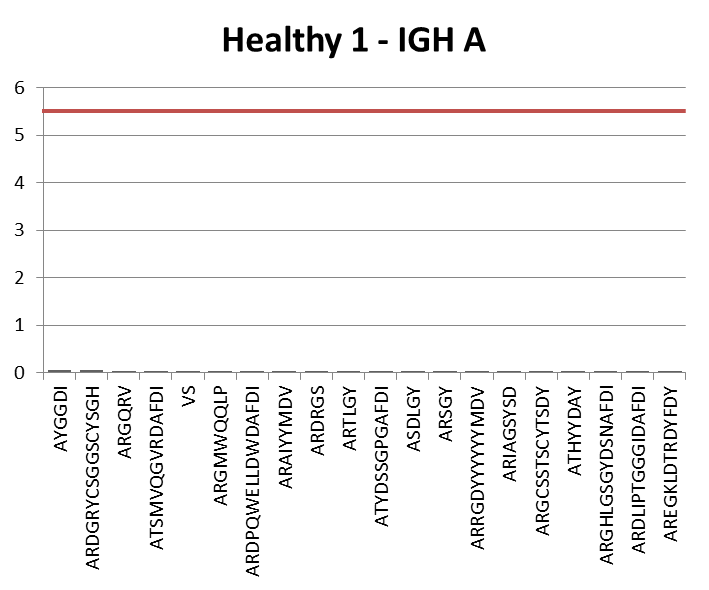

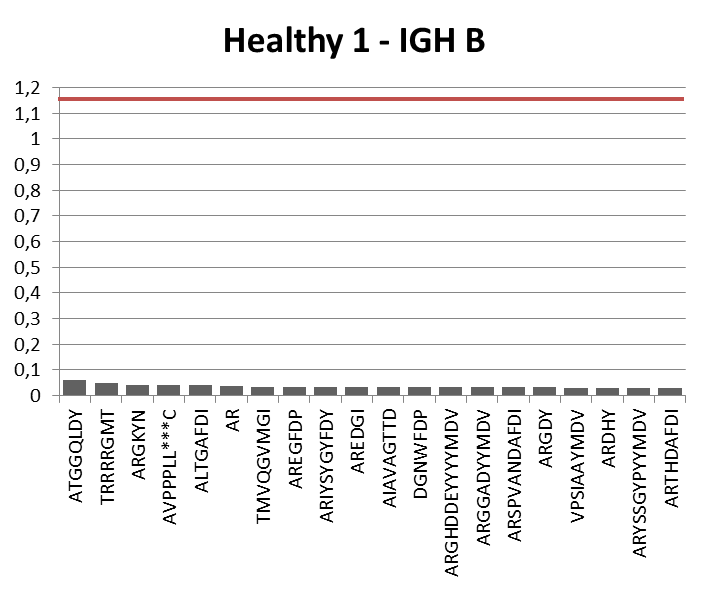


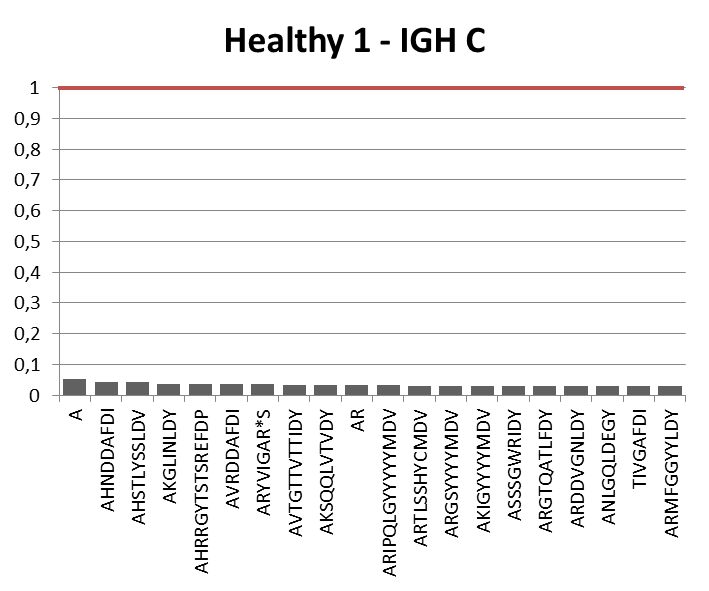


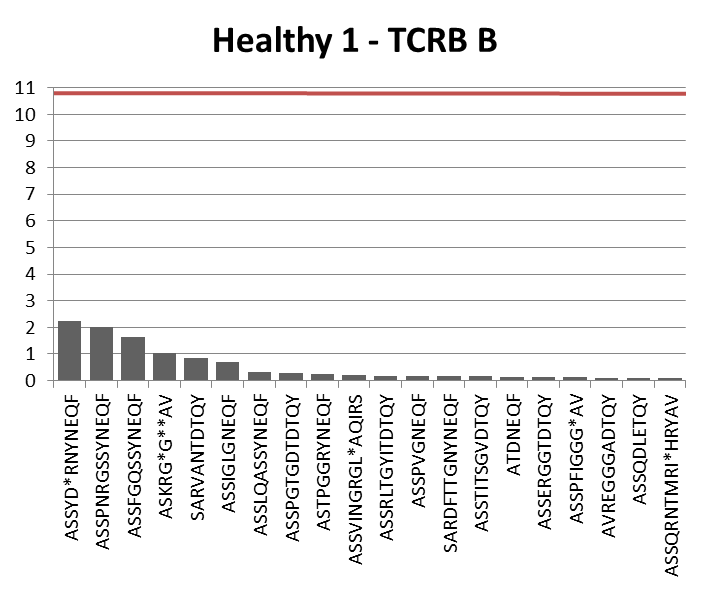

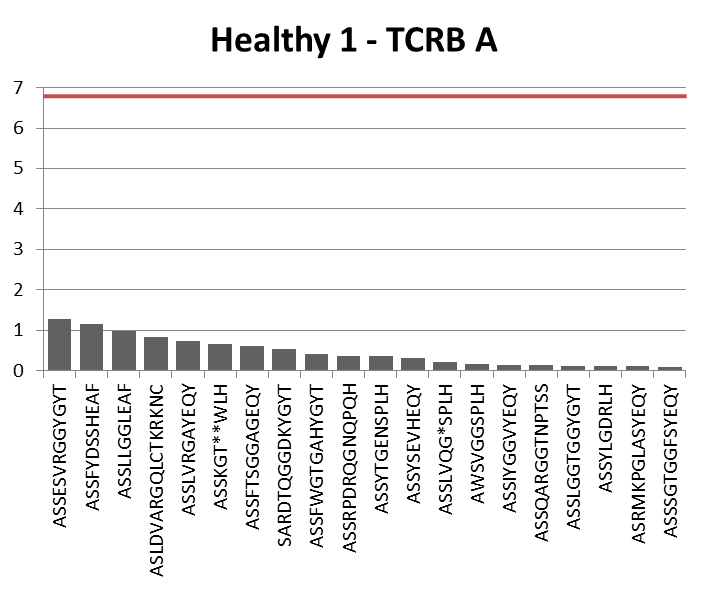


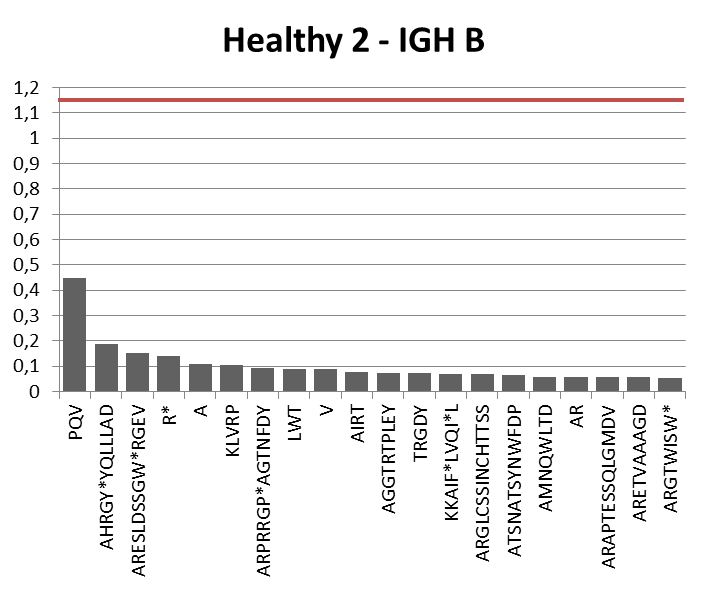

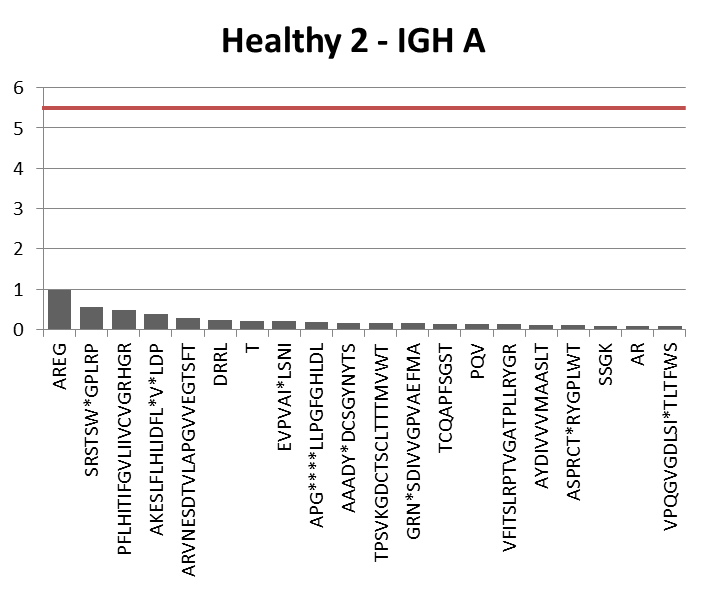


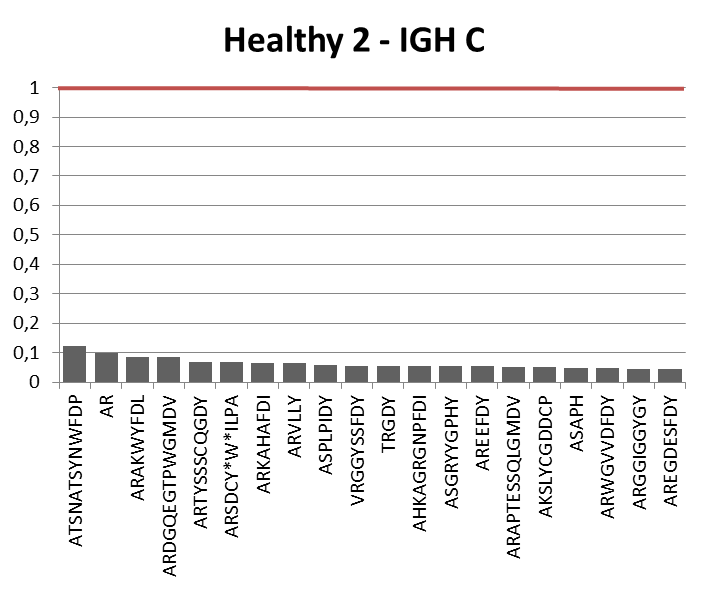


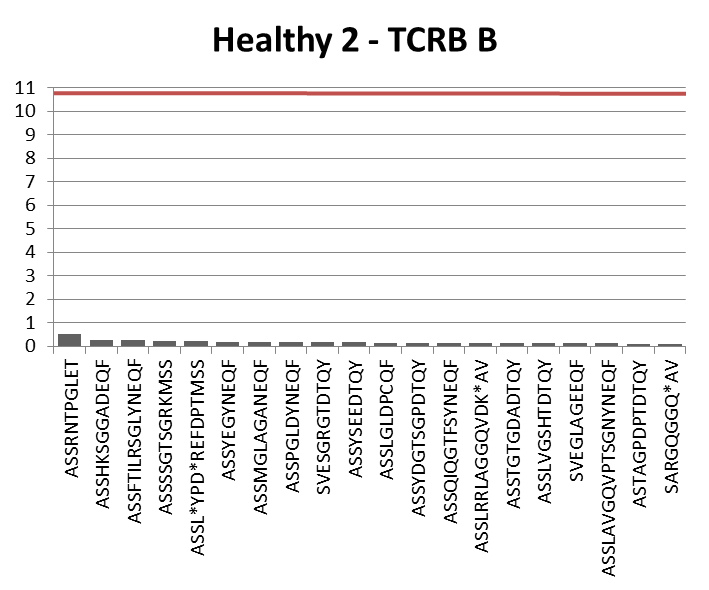

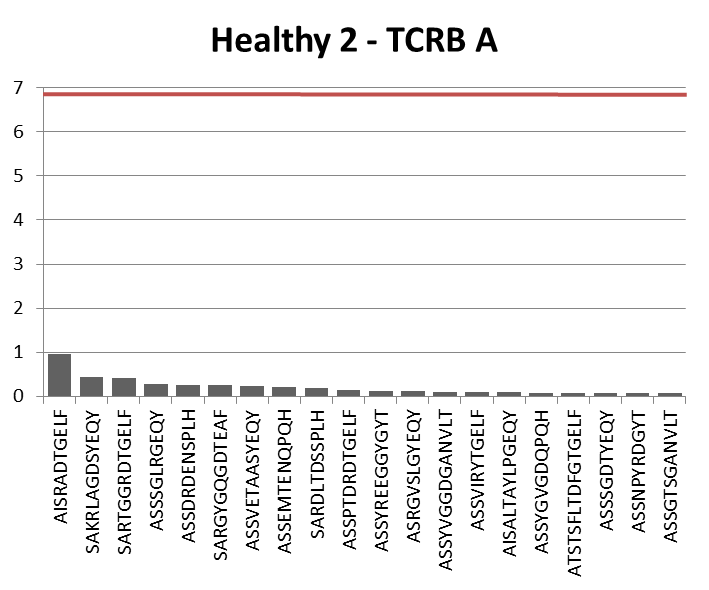


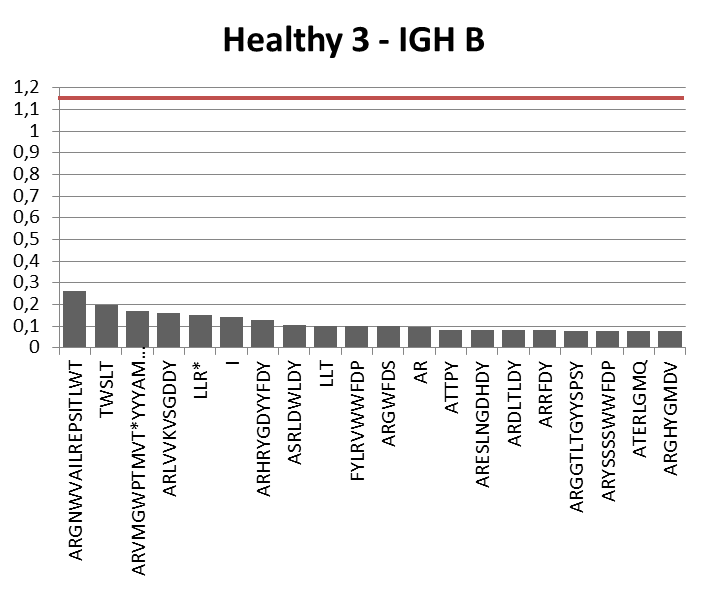

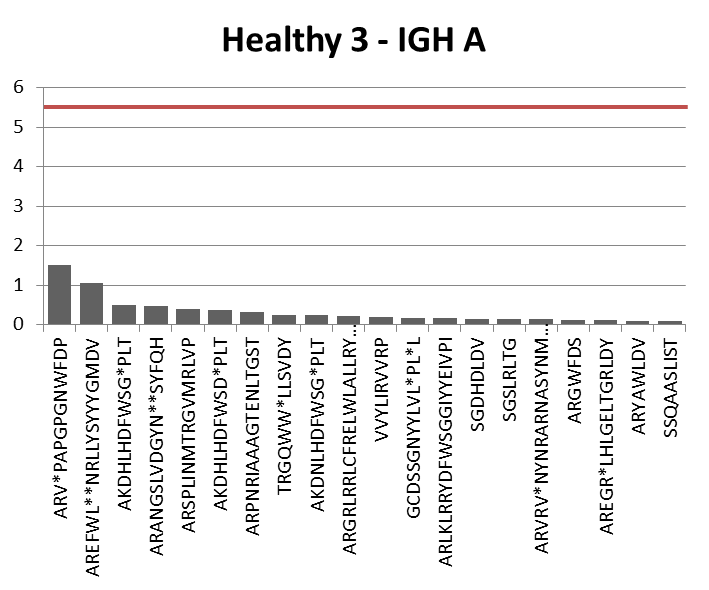


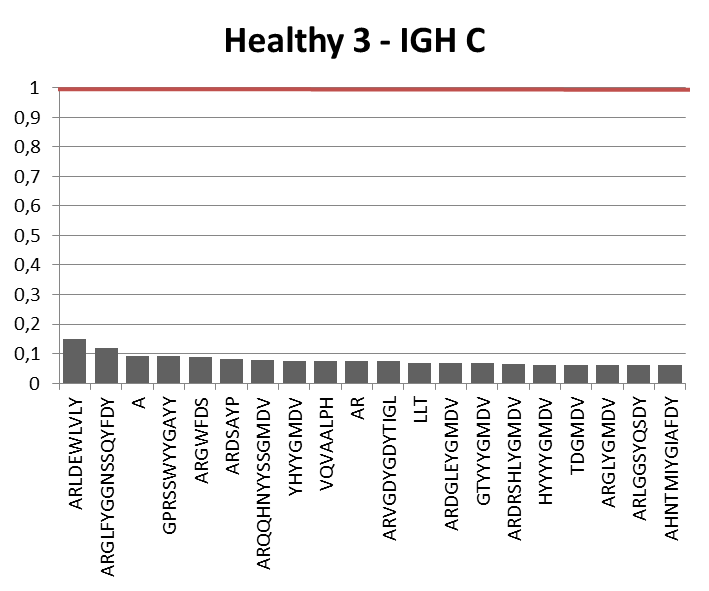


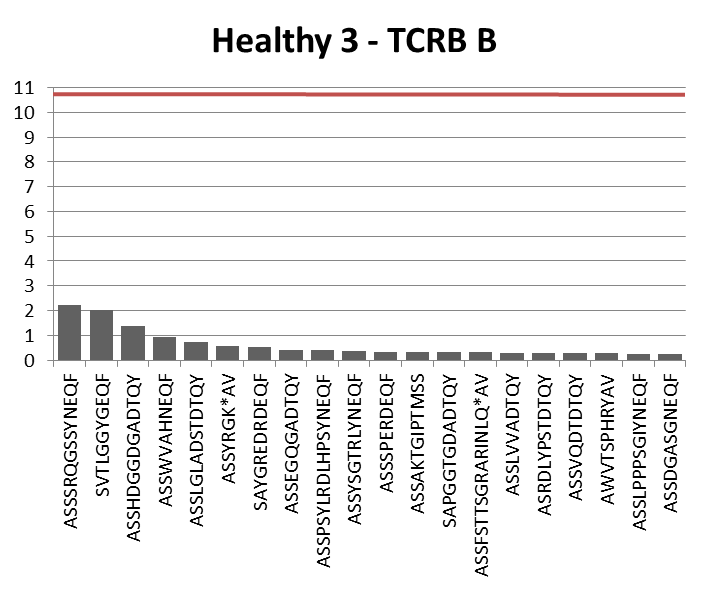

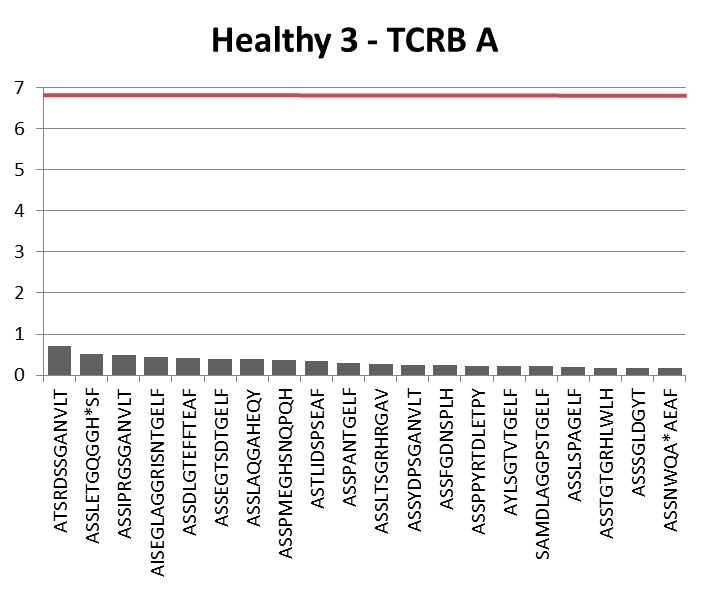


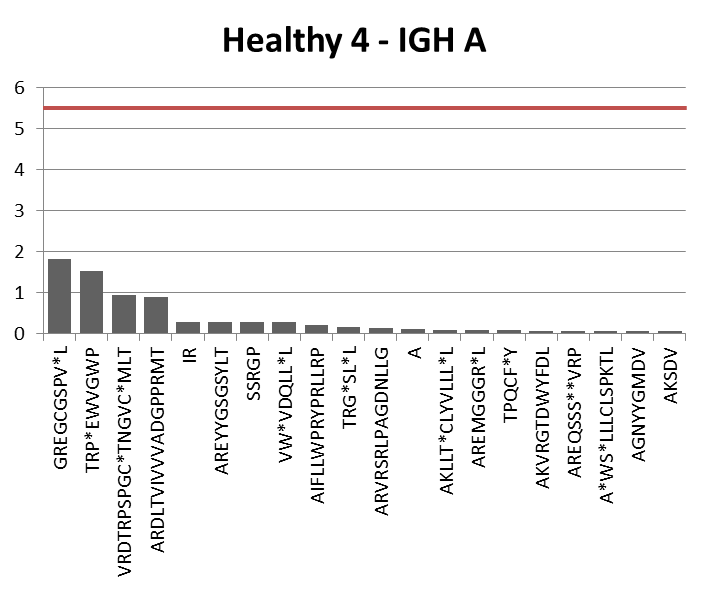

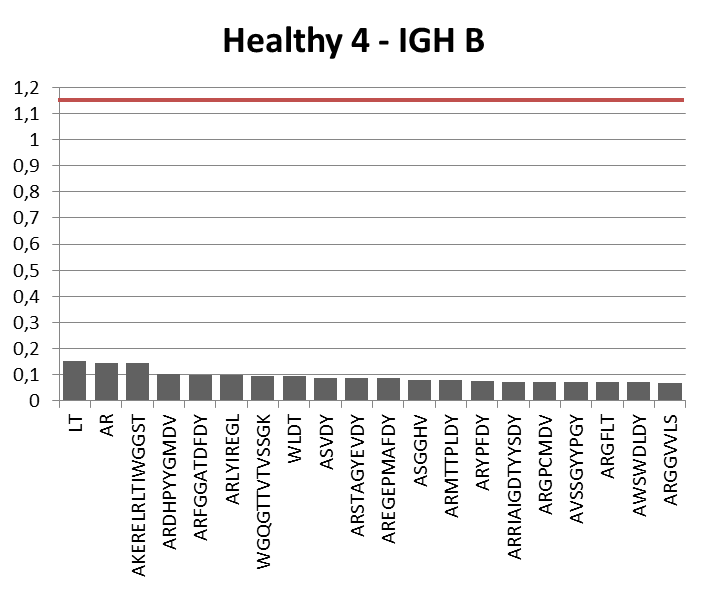


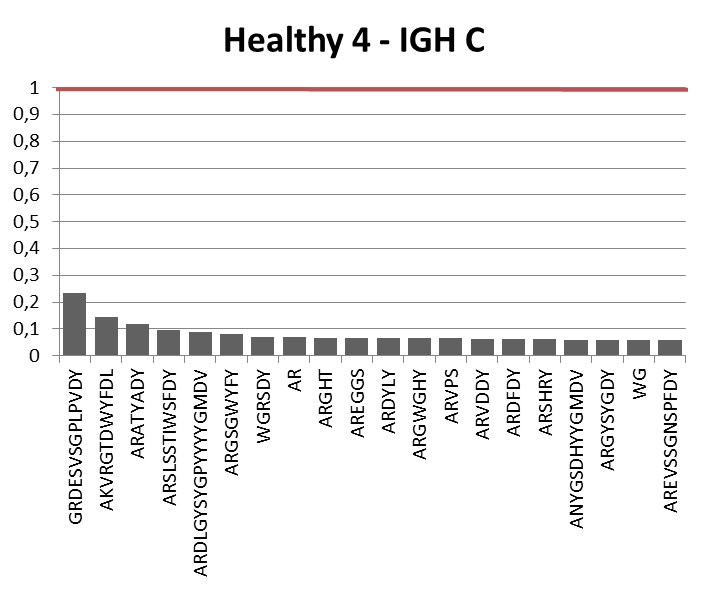


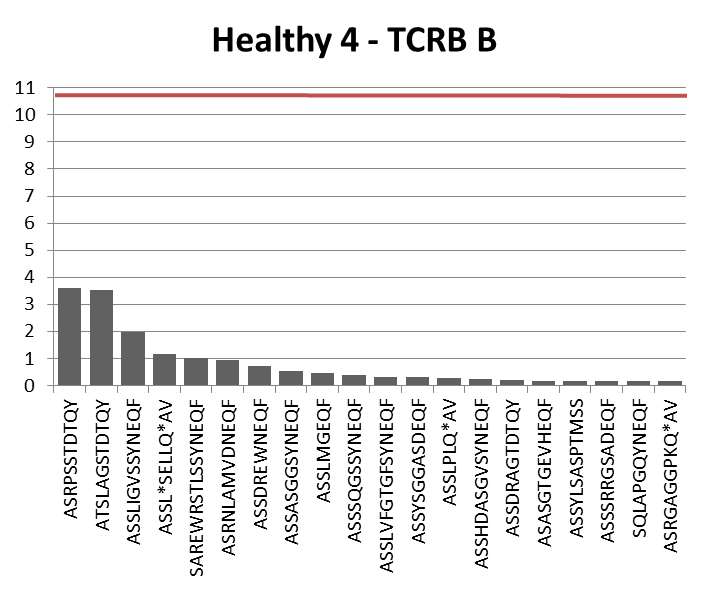

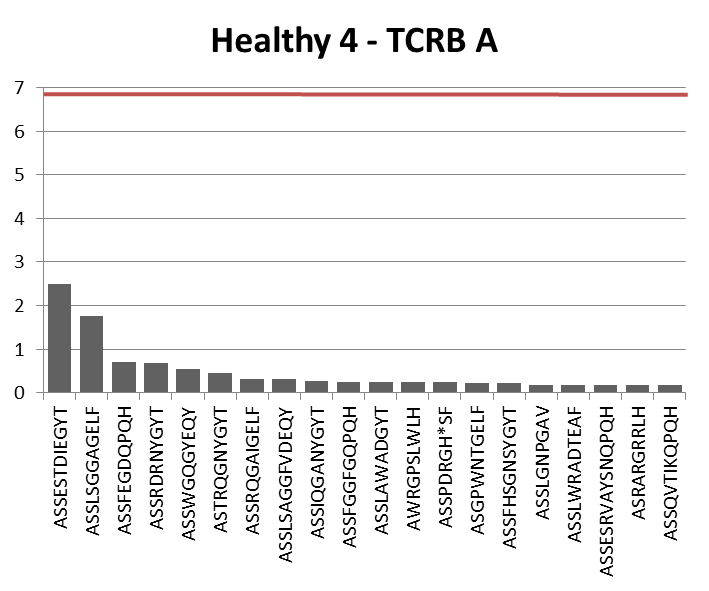


Patients Top20 CDR3 clones distribution for IGH primer set 1, 2 and 3, TRB primer set 1 and 2 in the blood and in the kidney


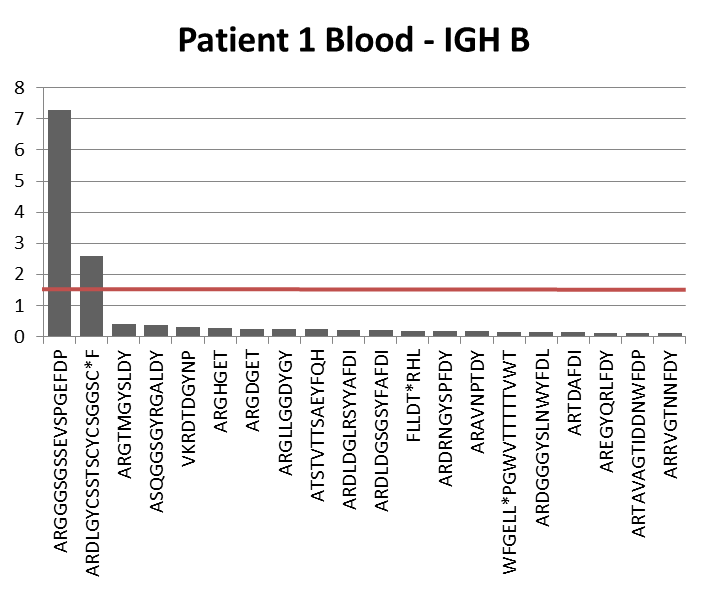

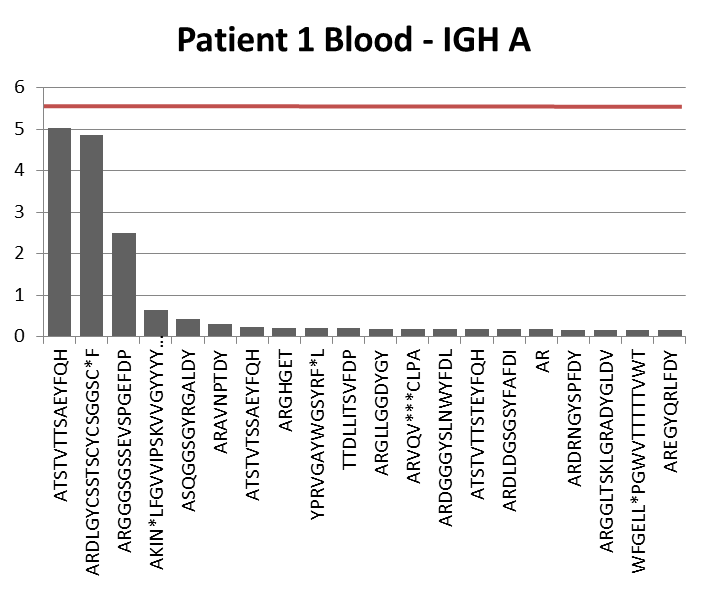


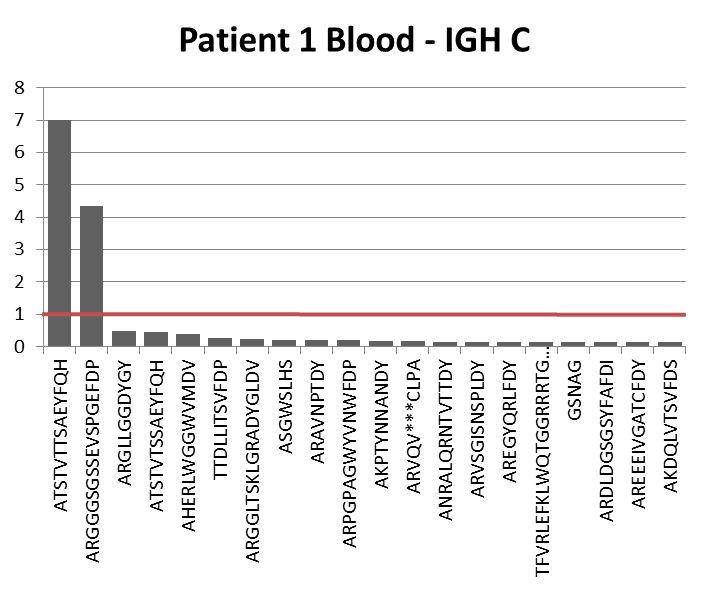


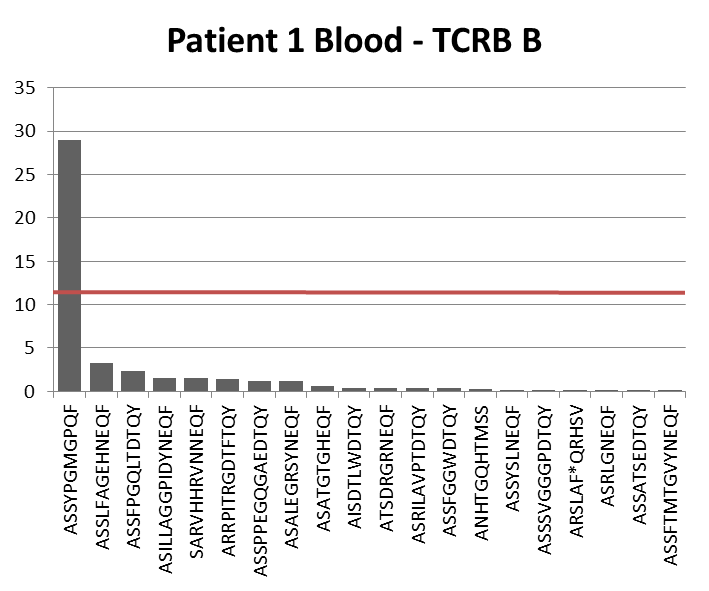

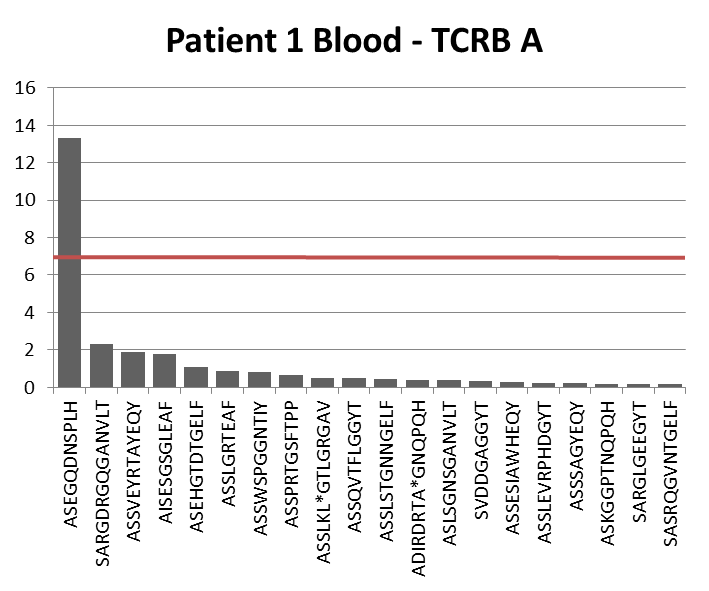


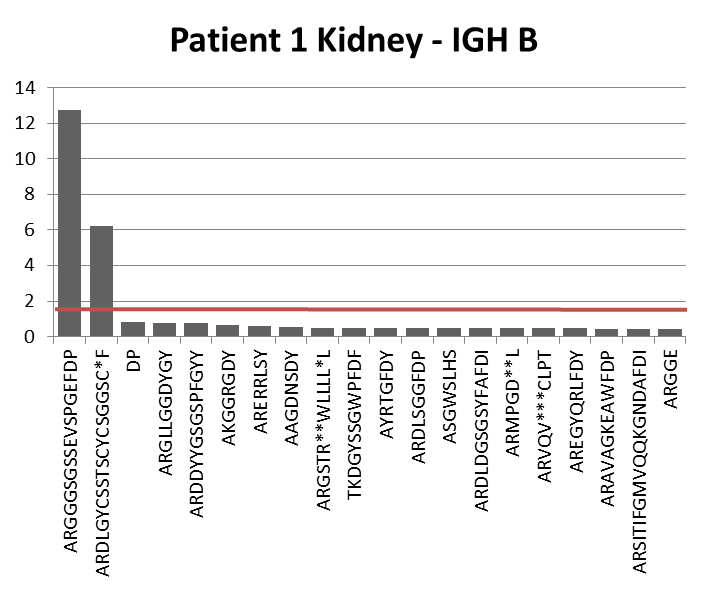

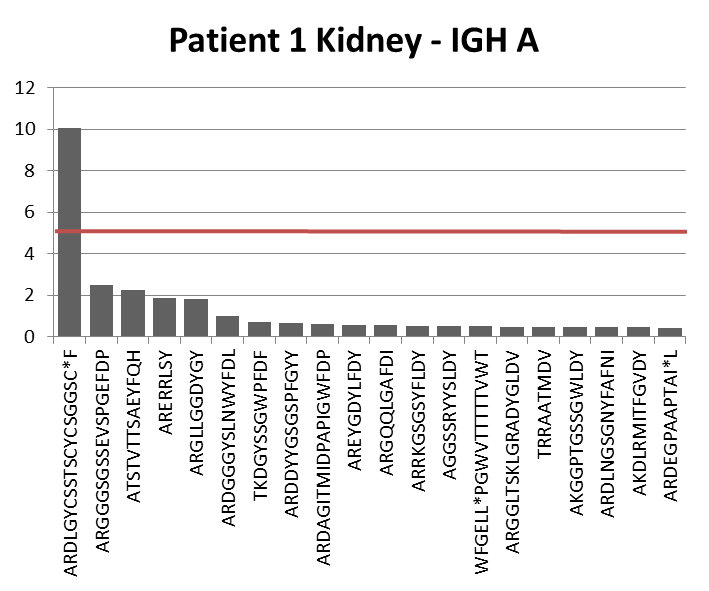


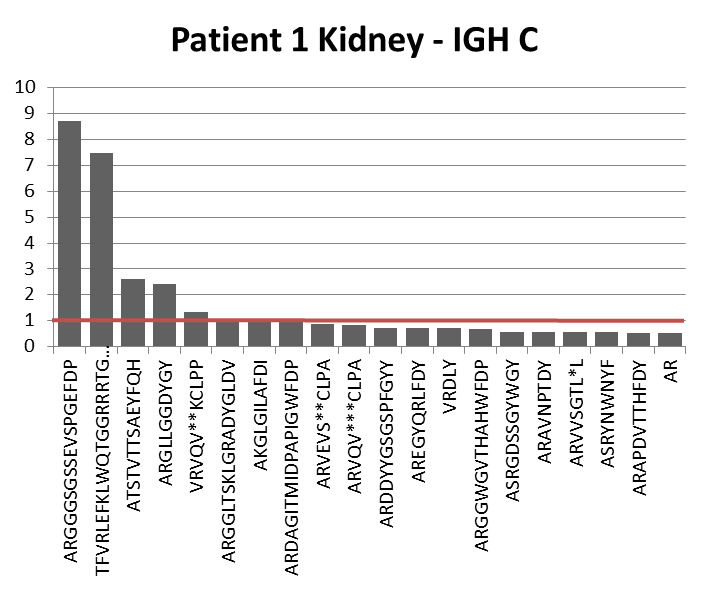


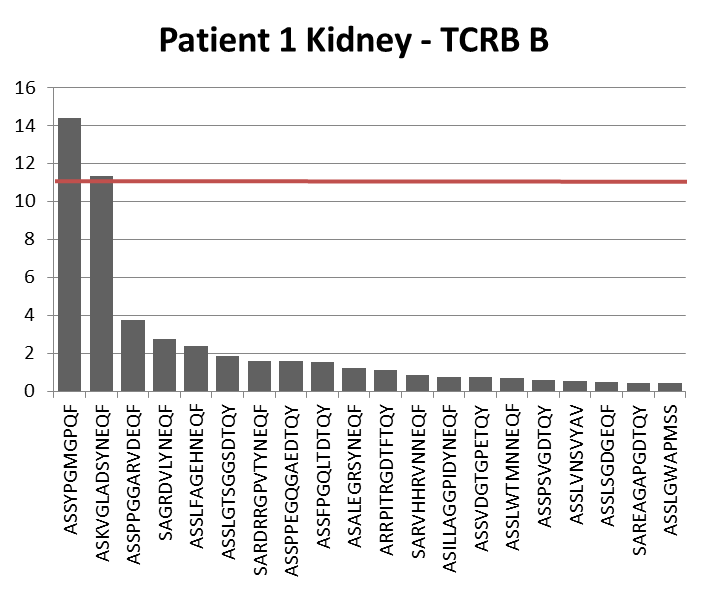

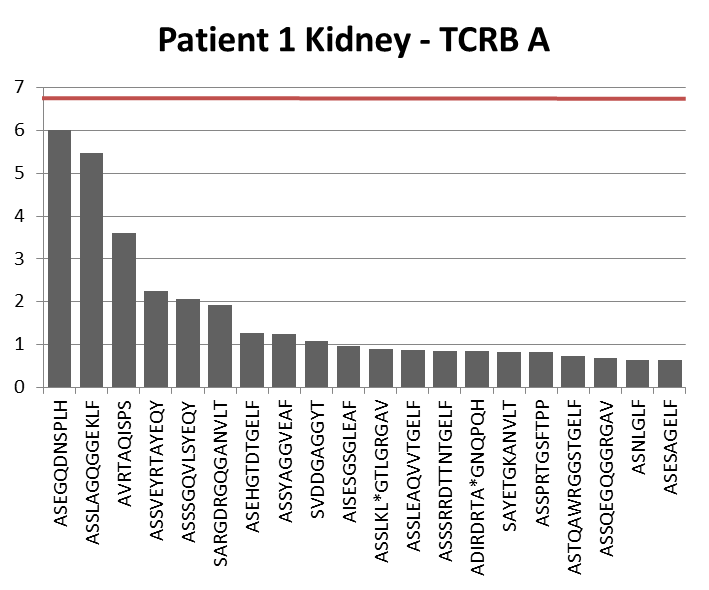


**NO IGH PRIMER SET 1 NOR 2 FOR PATIENT 2 BLOOD/KIDNEY**


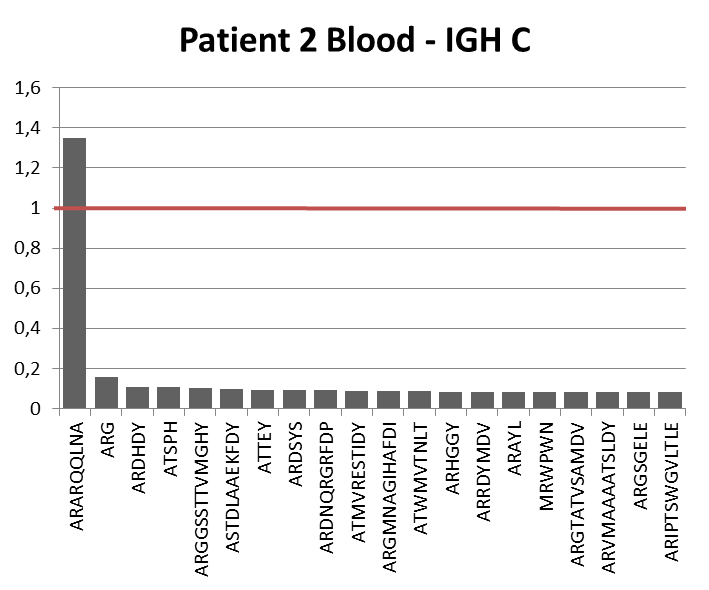


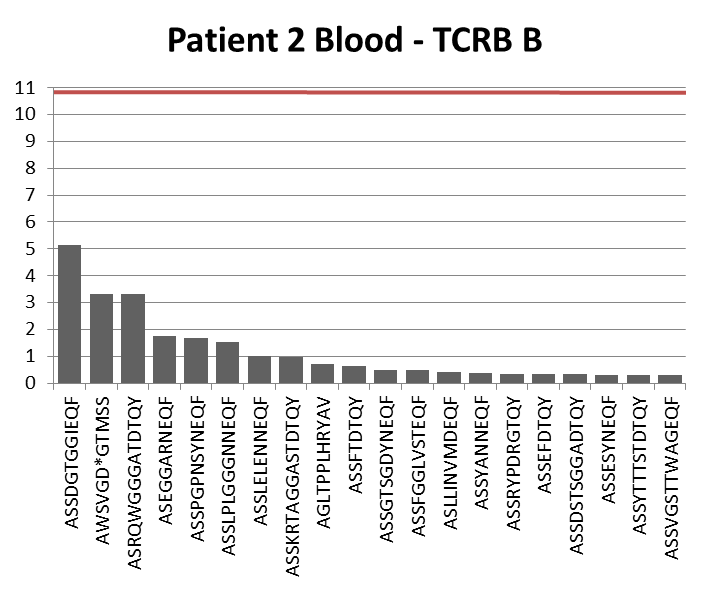

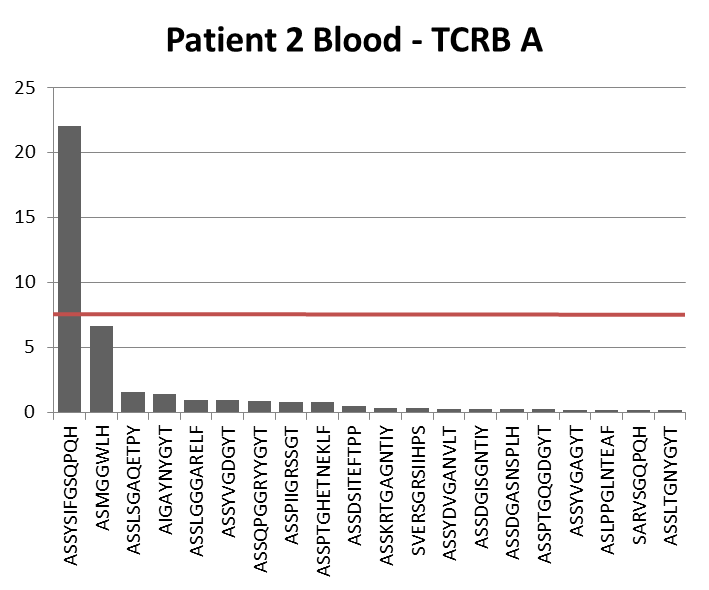


**NO IGH PRIMER SET 1 NOR 2 FOR PATIENT 2 BLOOD/KIDNEY**


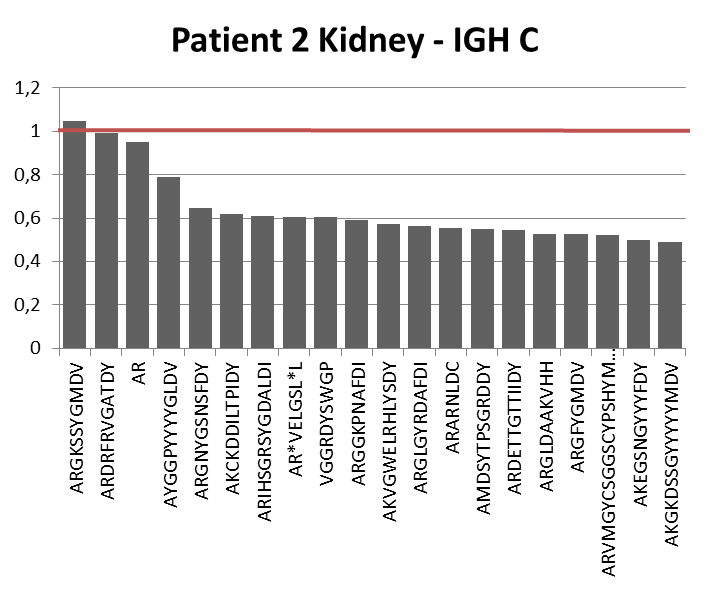


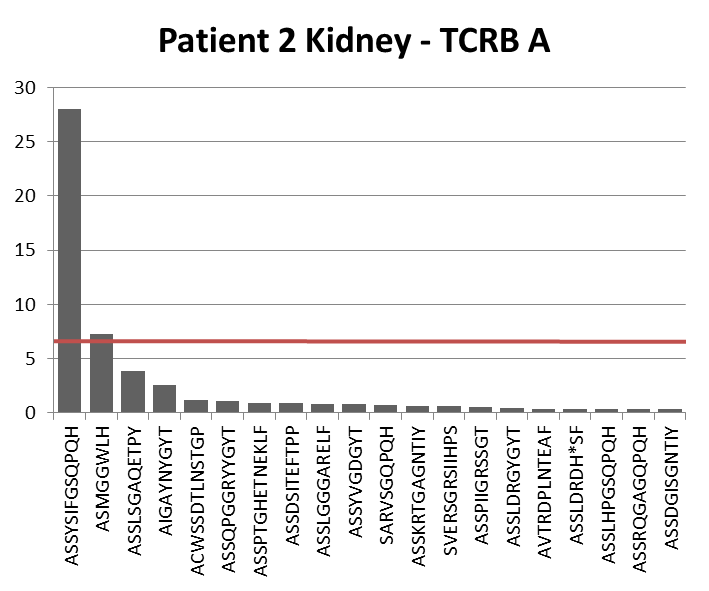


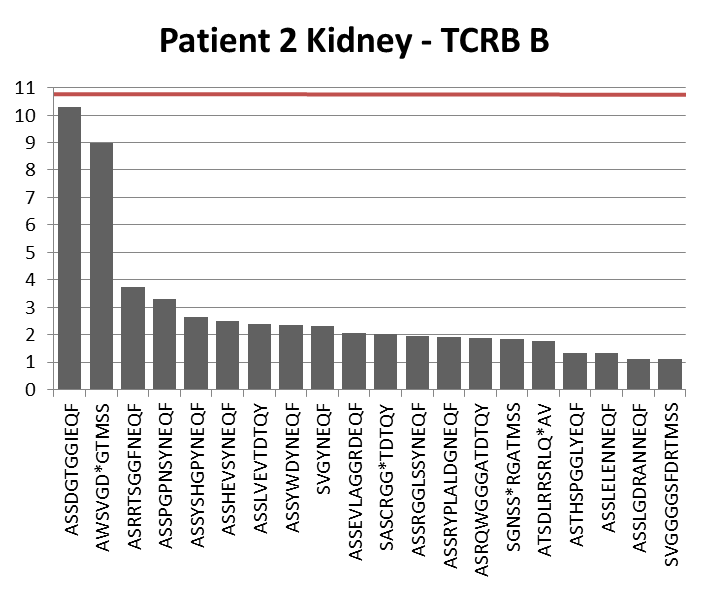


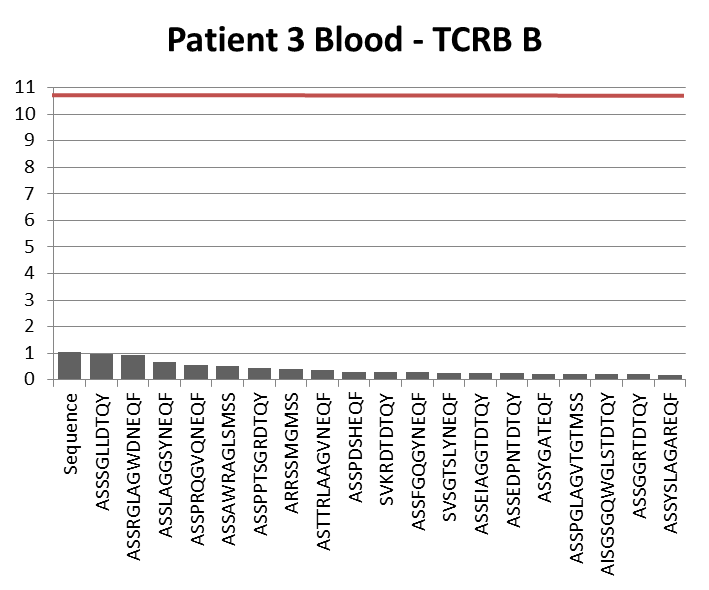

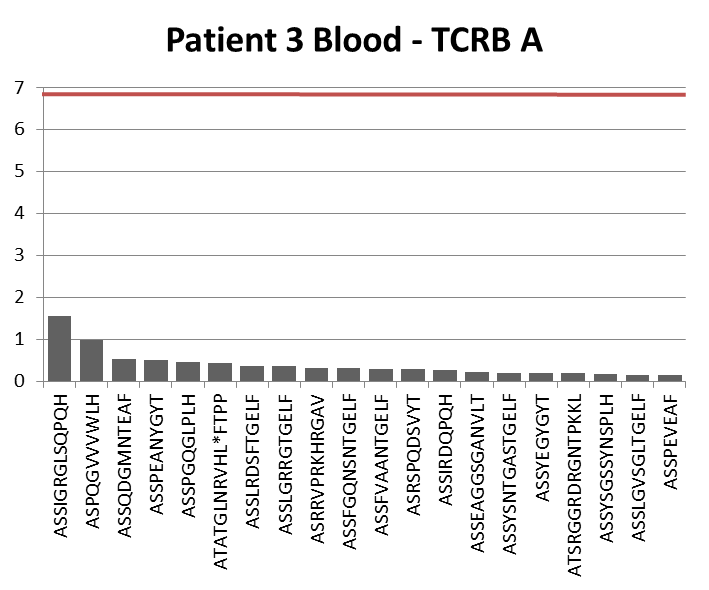

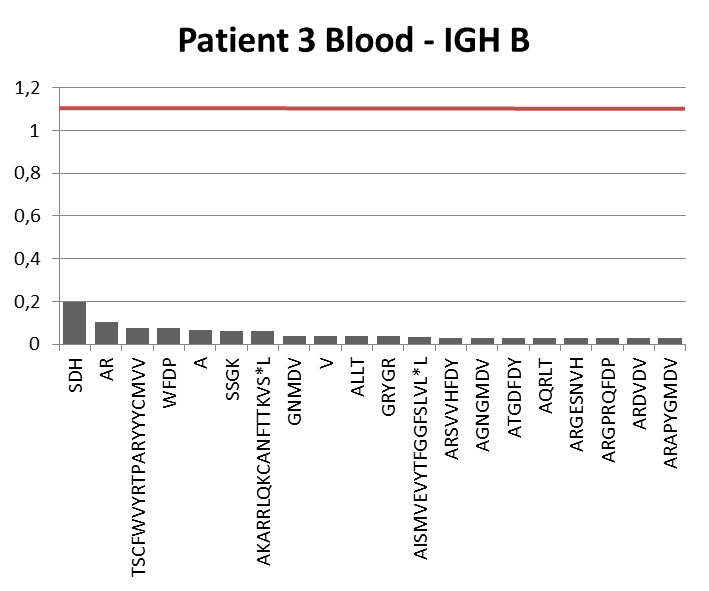

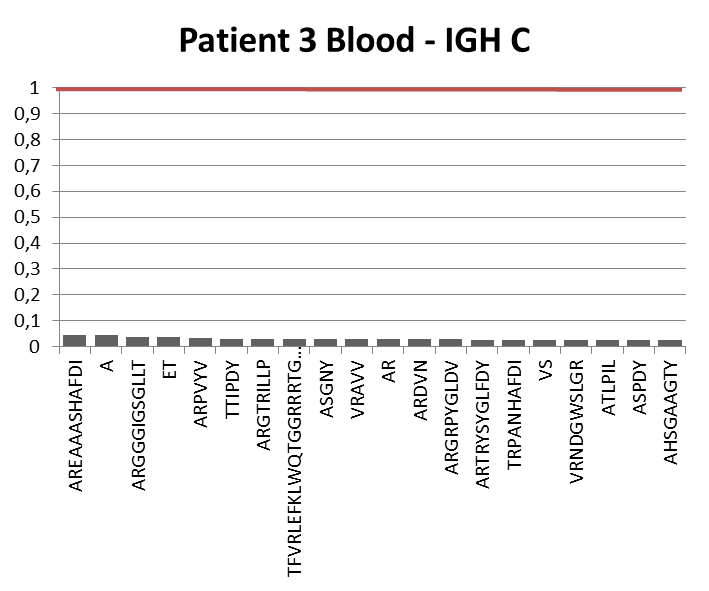

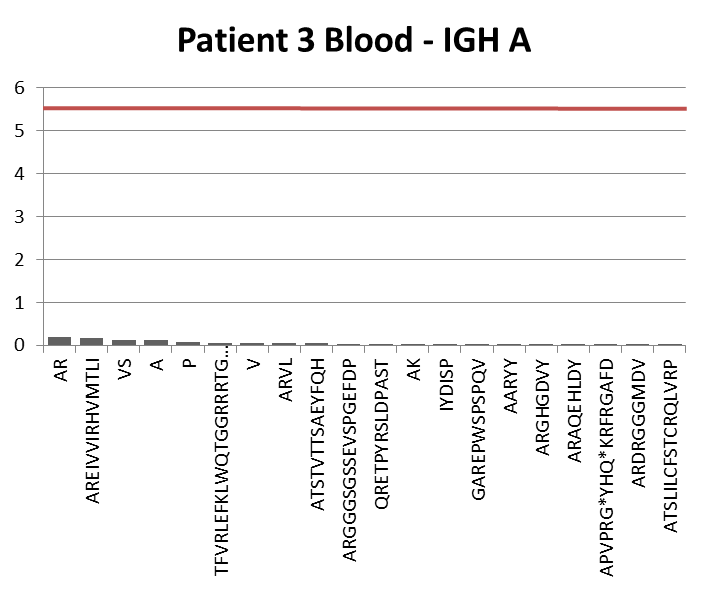


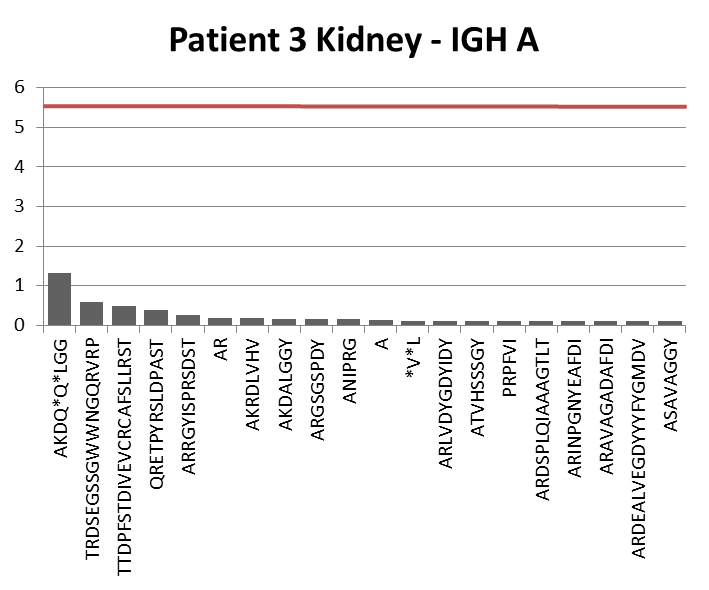

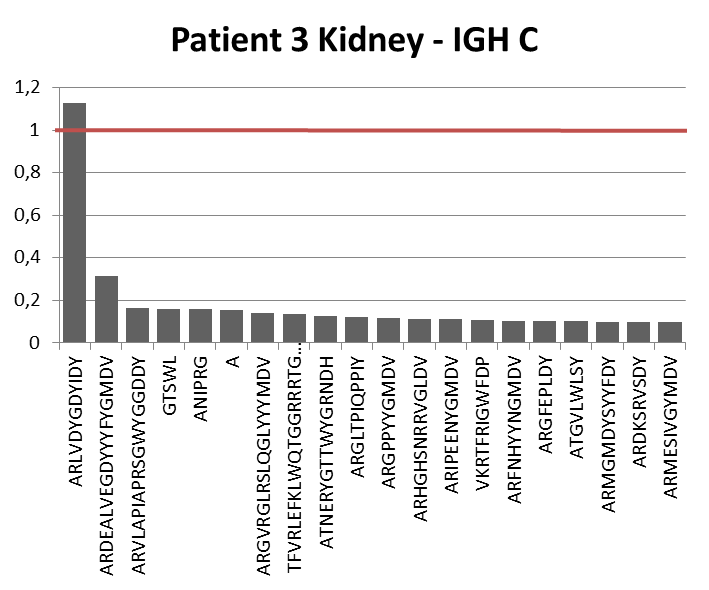

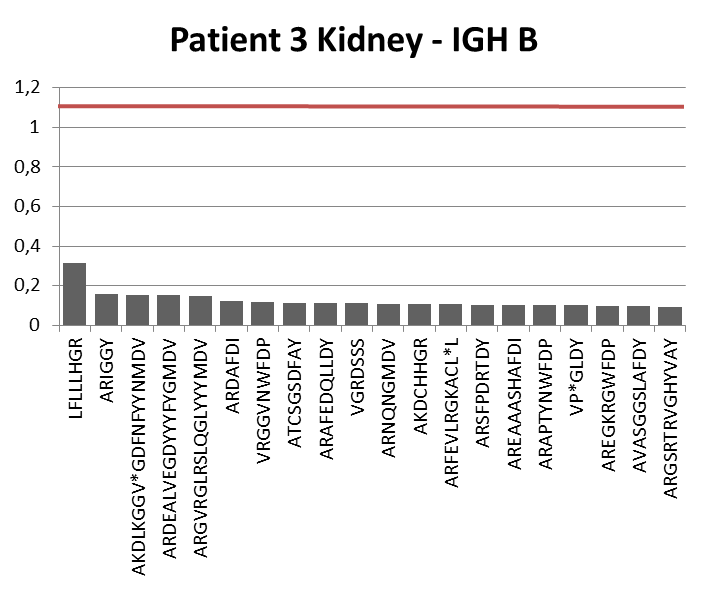

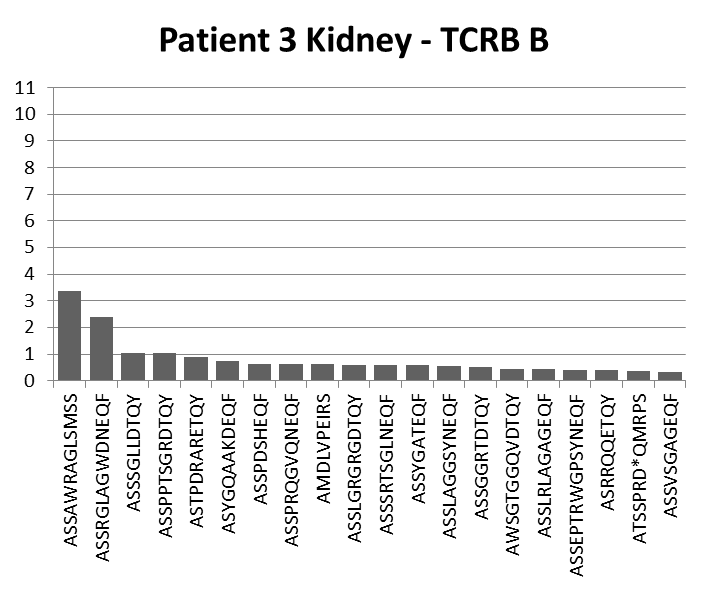

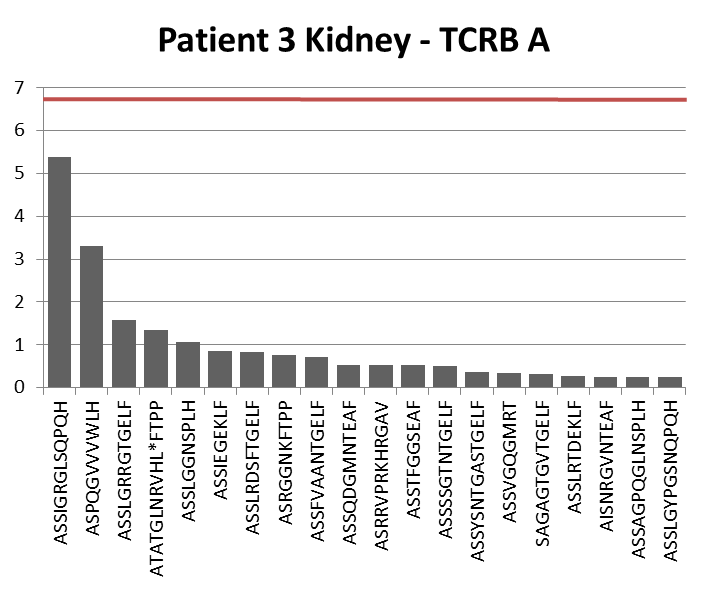


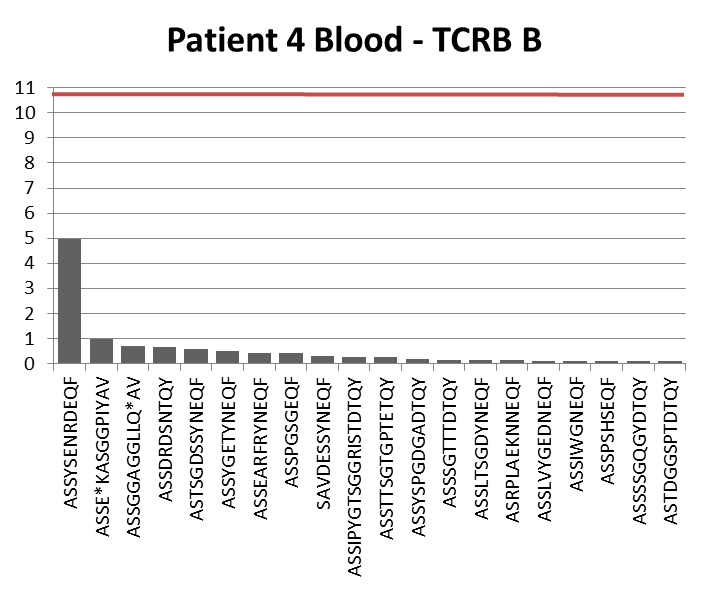

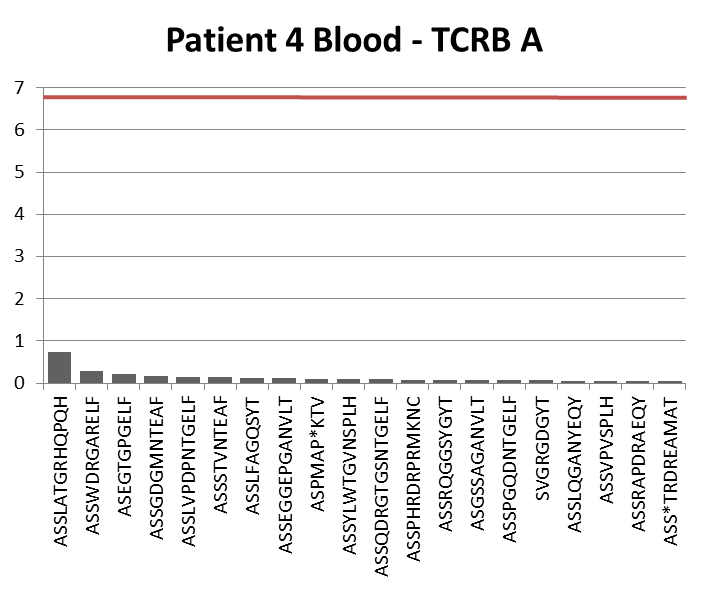

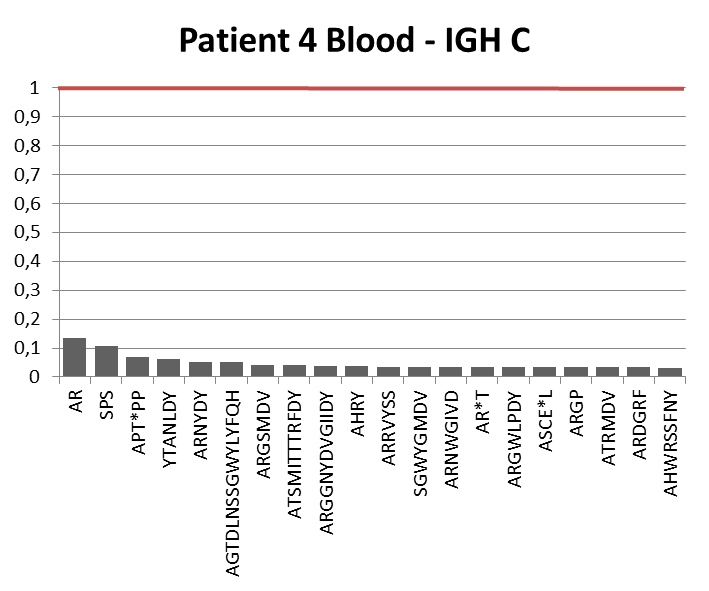

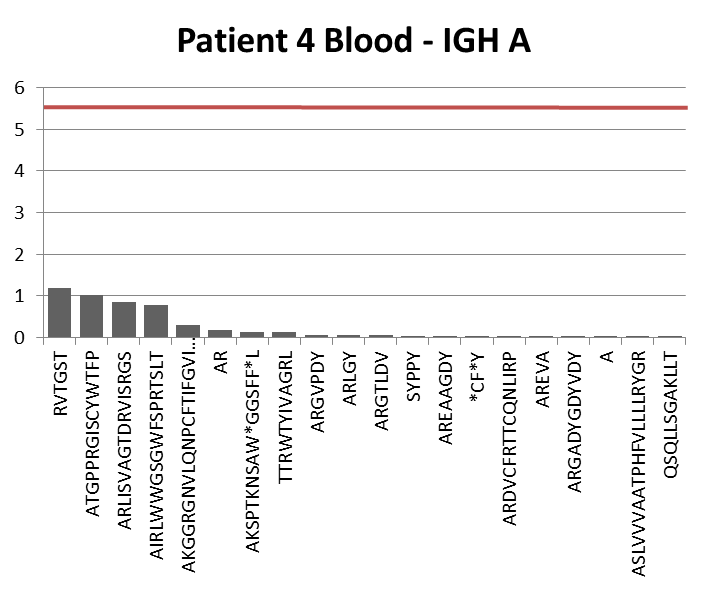

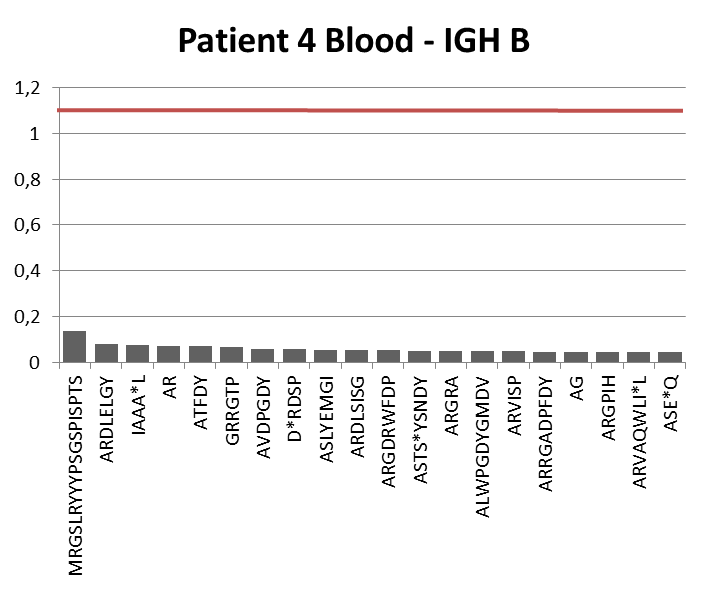


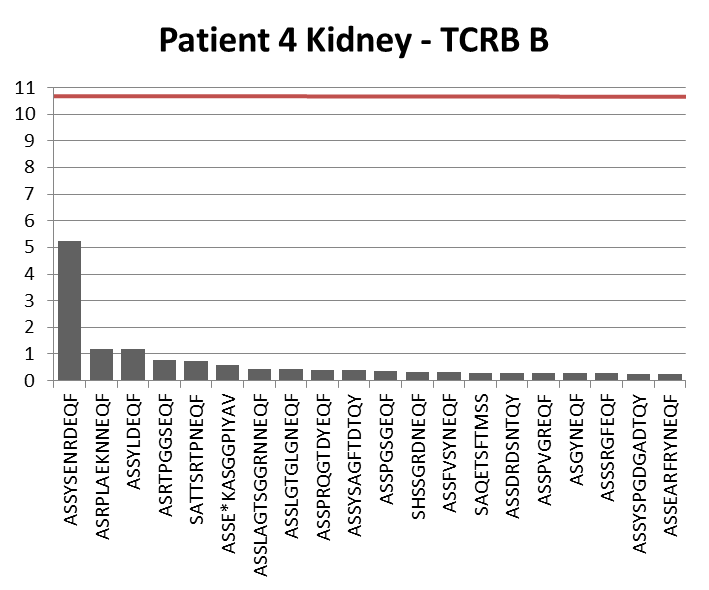

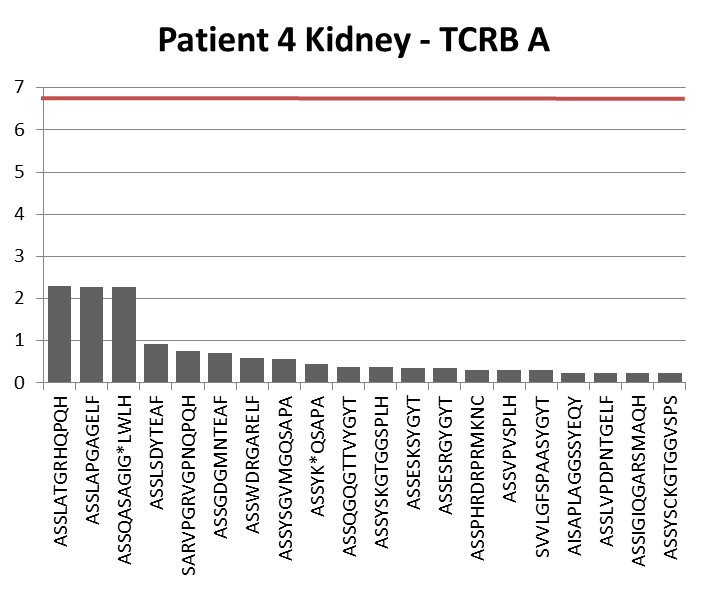

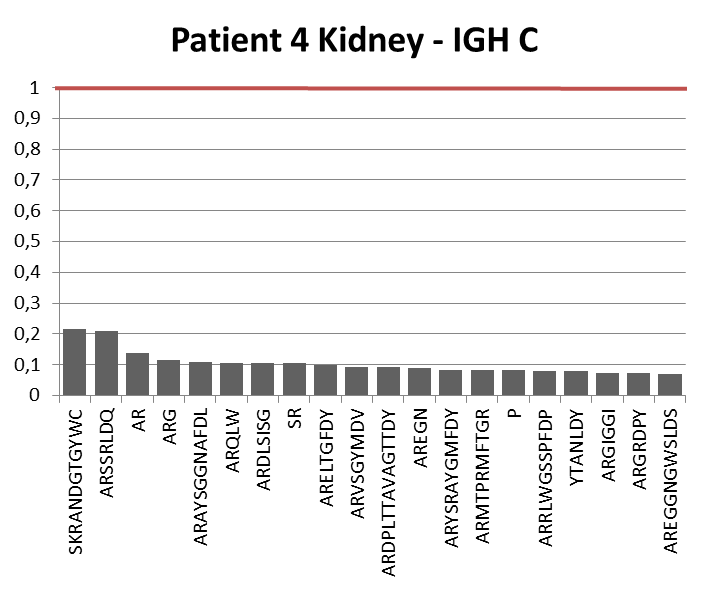

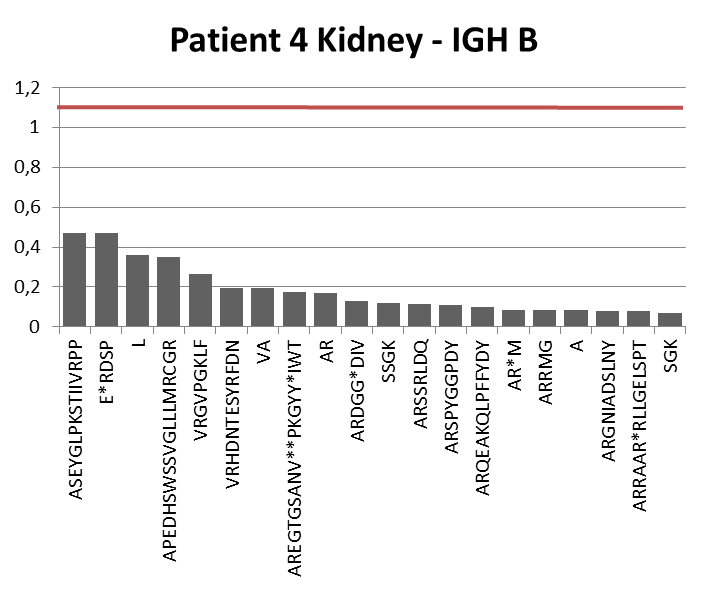

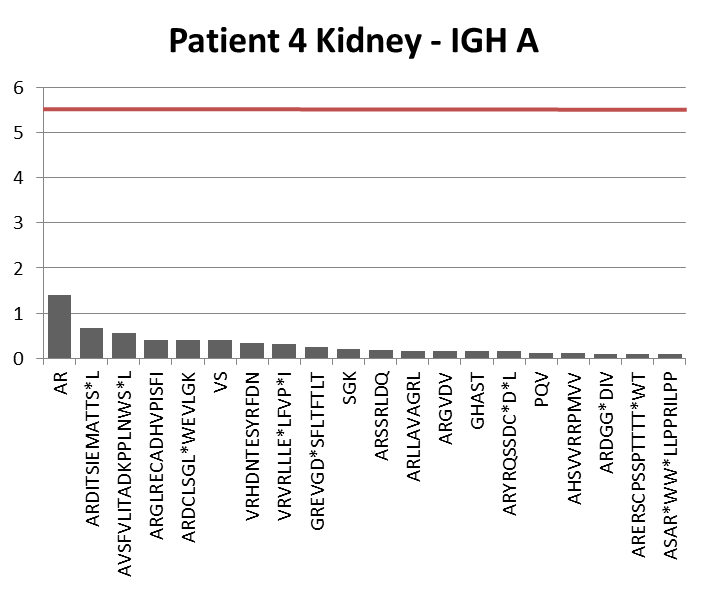


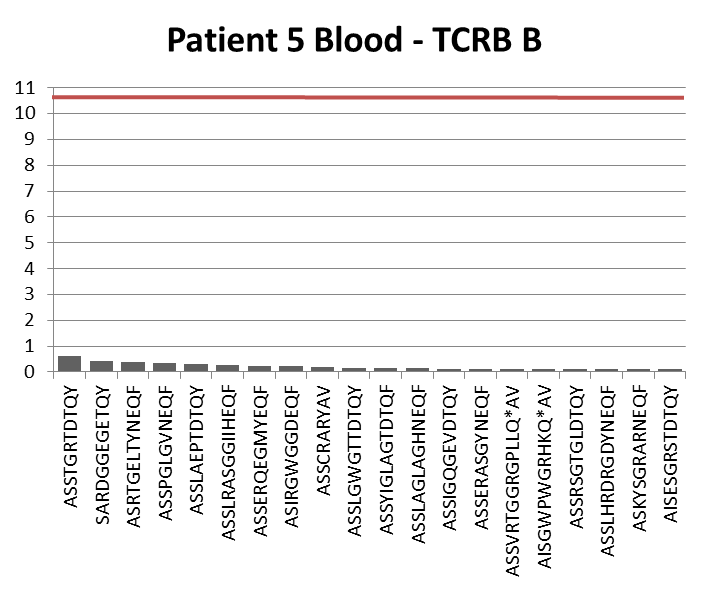

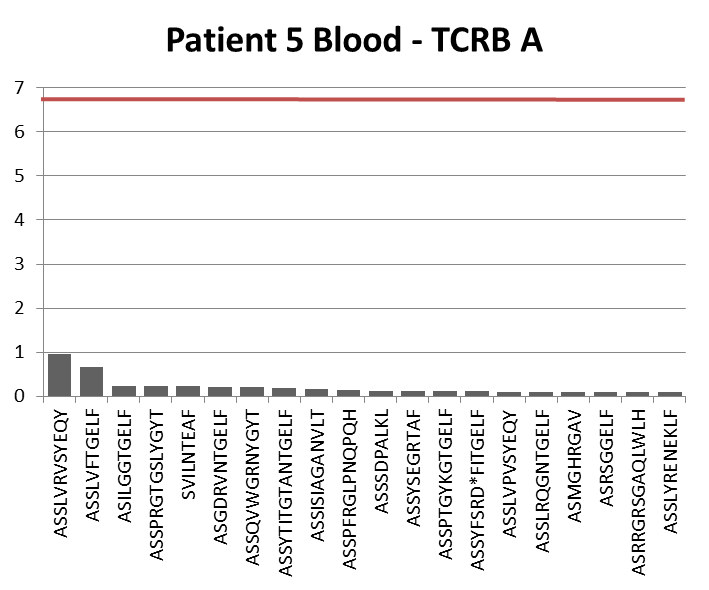

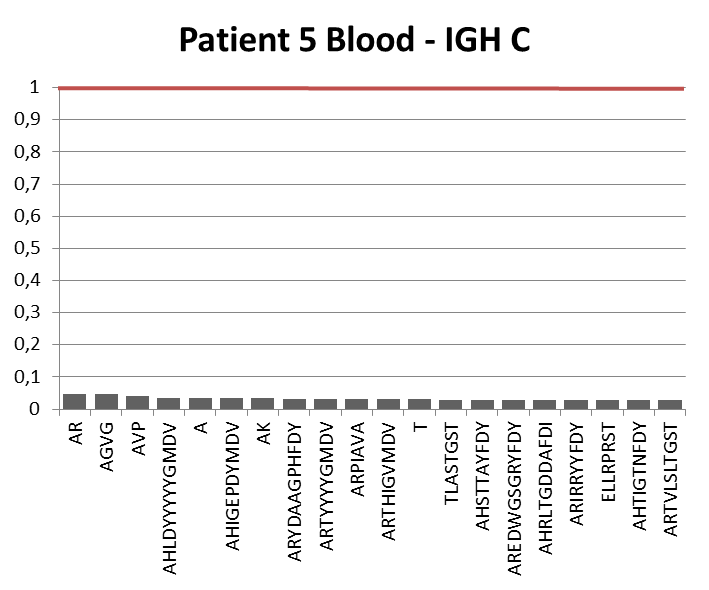

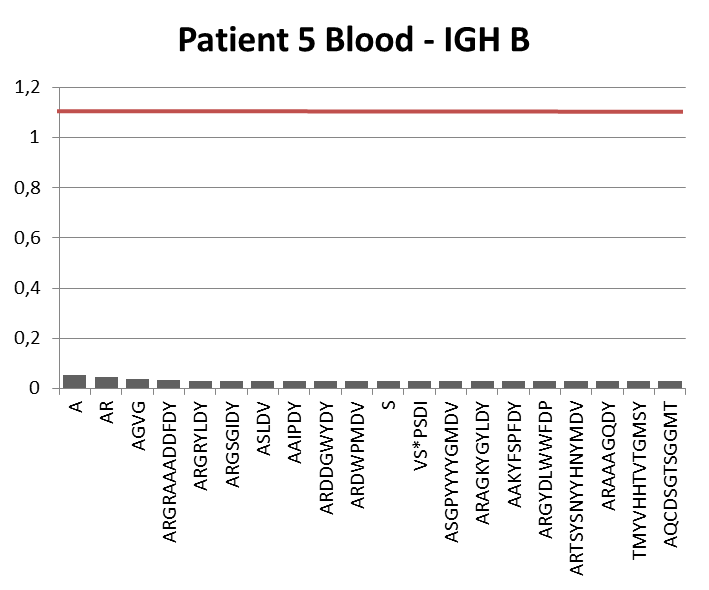

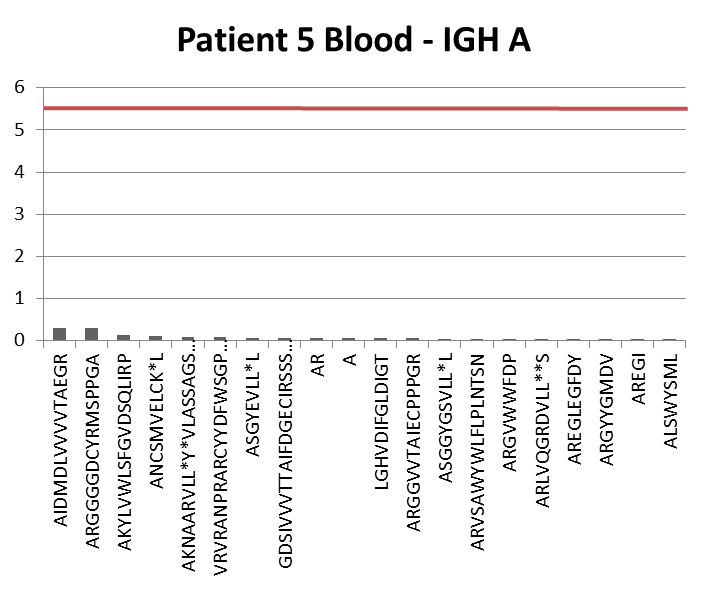


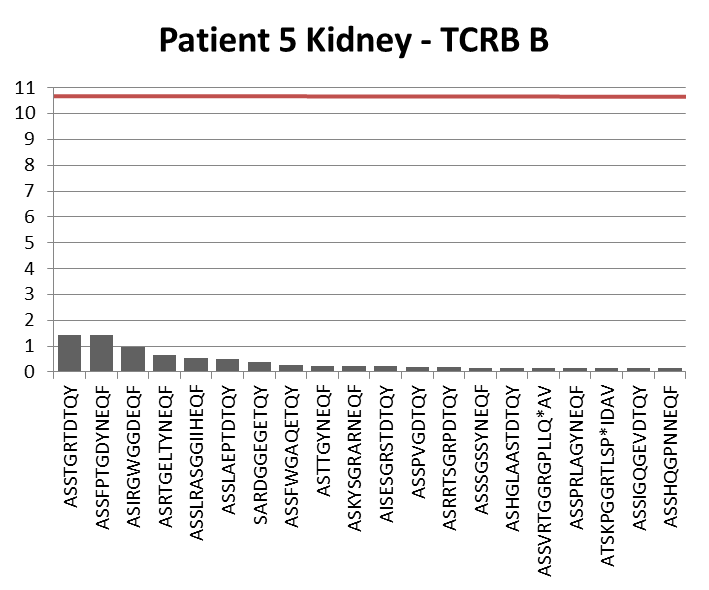

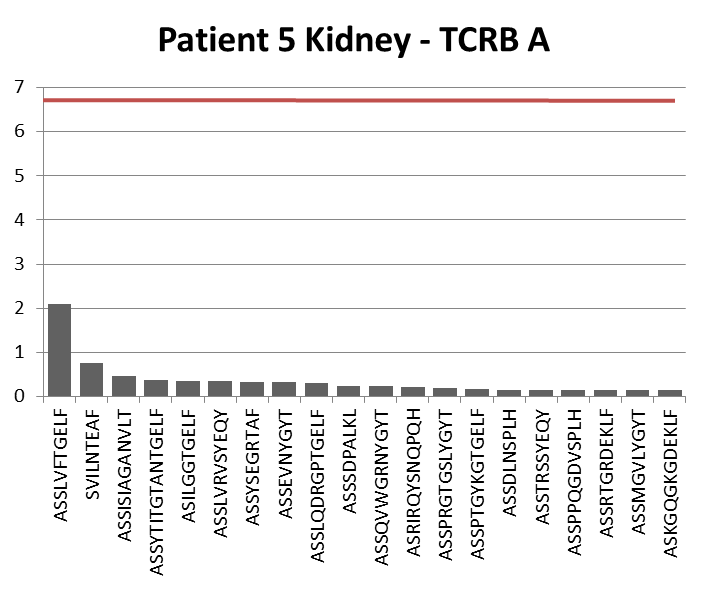

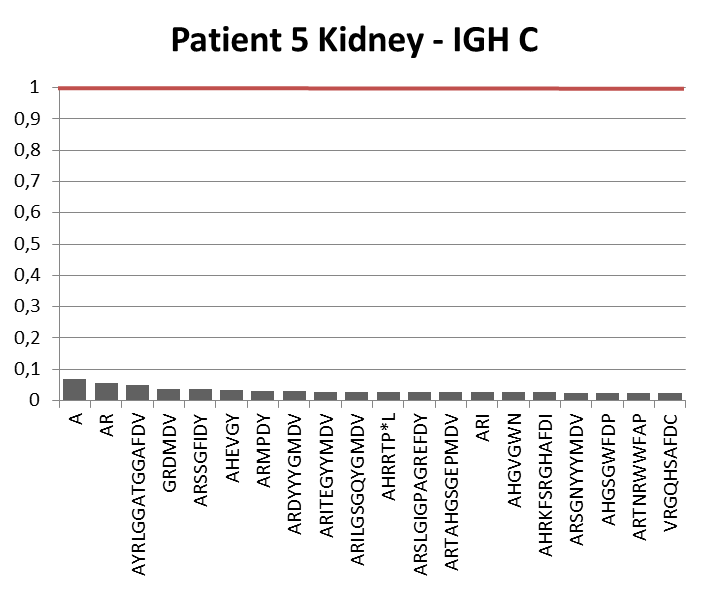

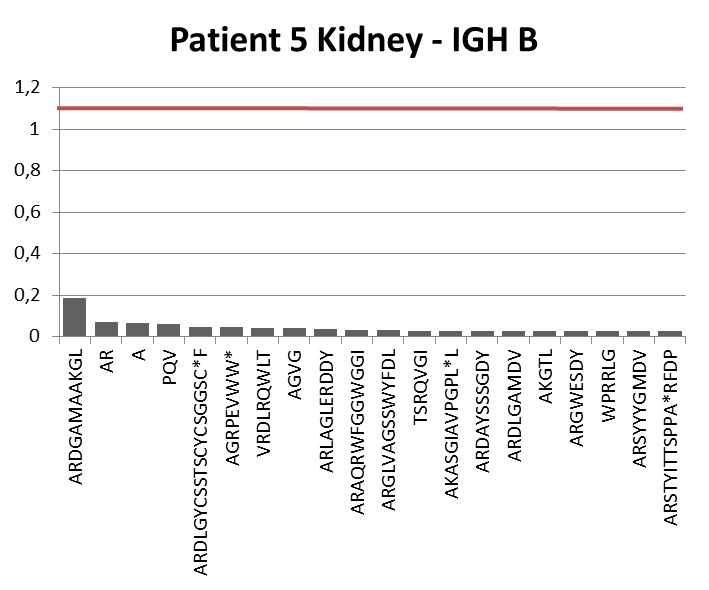

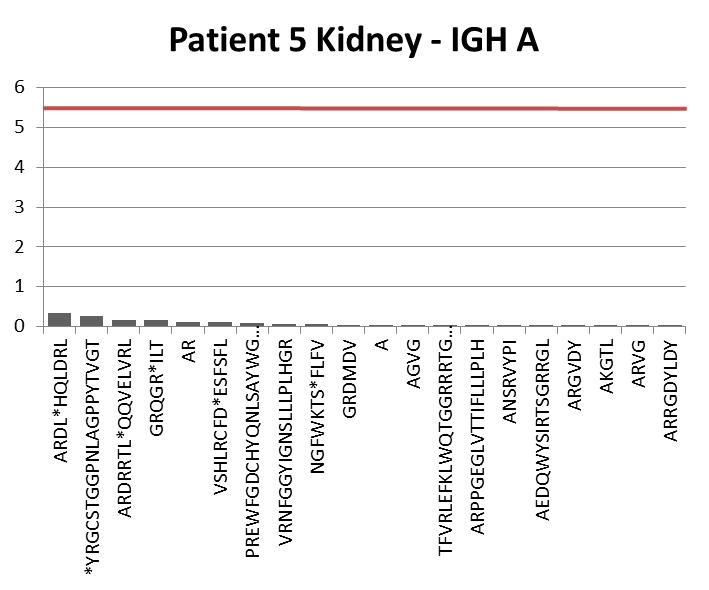


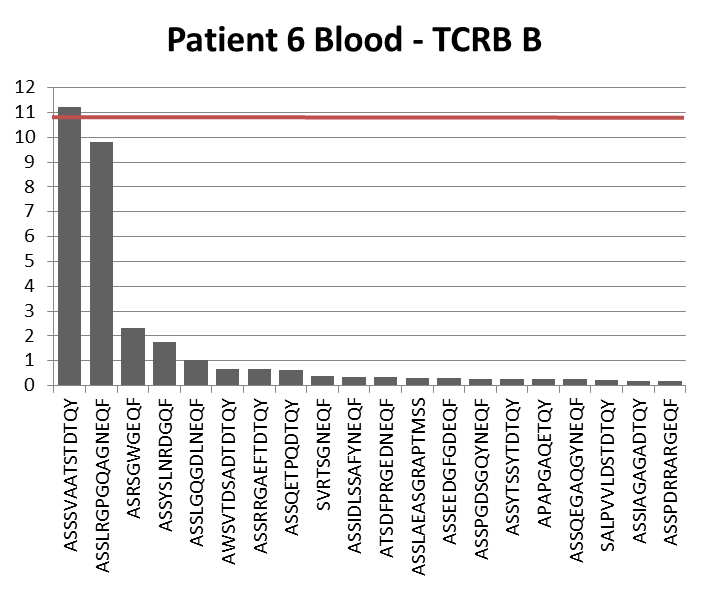

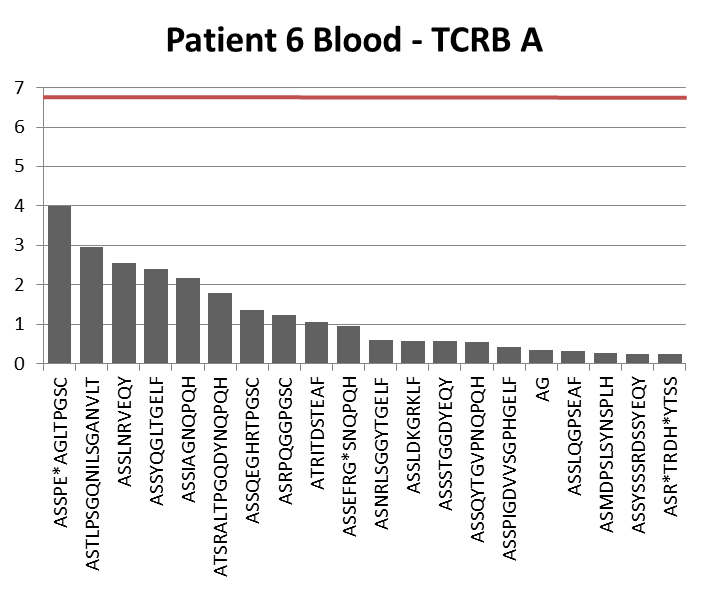

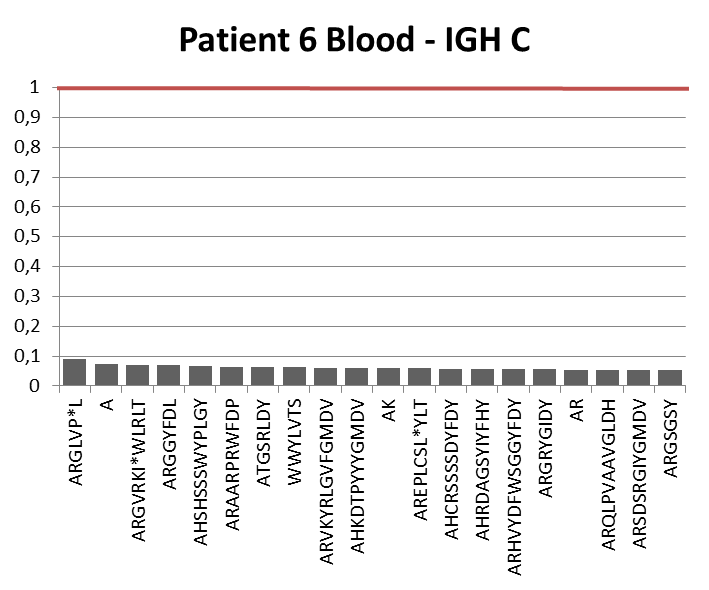

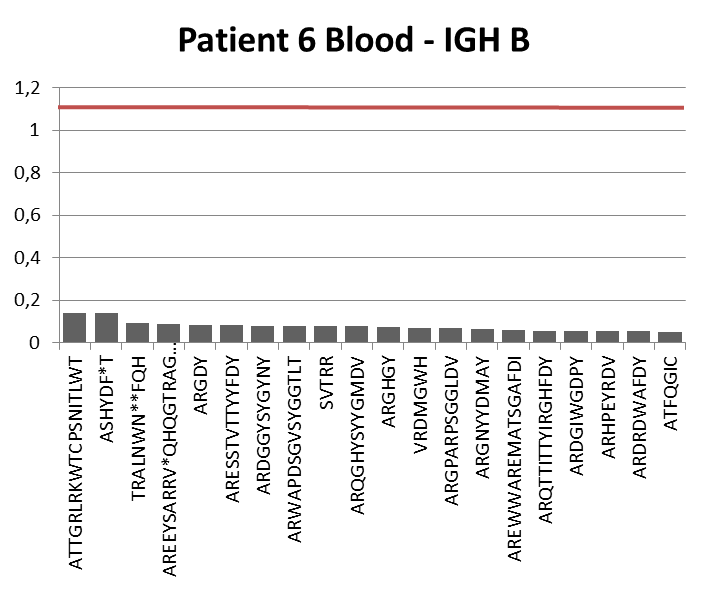

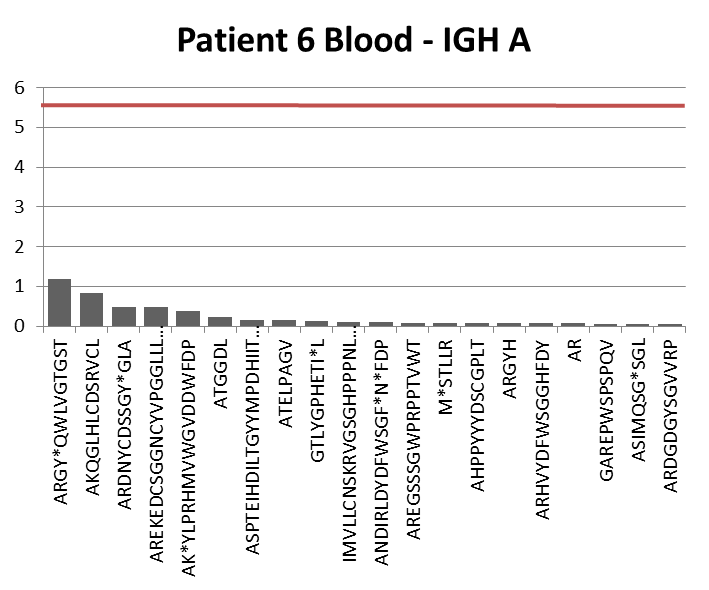


**NO IGH PRIMER SET 1, 2 AND 3 FOR PATIENT 6 KIDNEY**


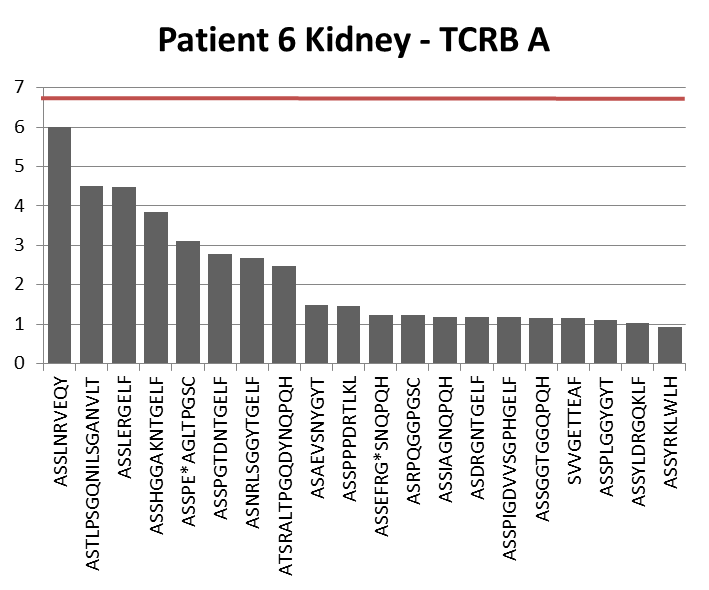


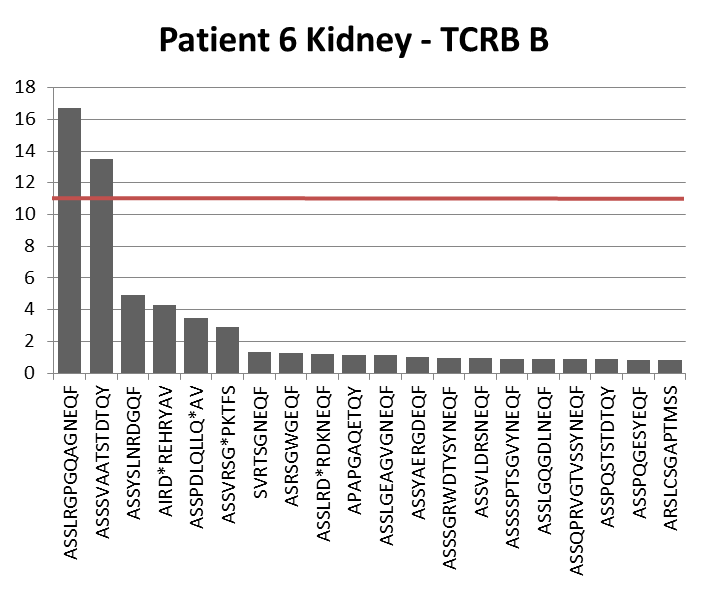


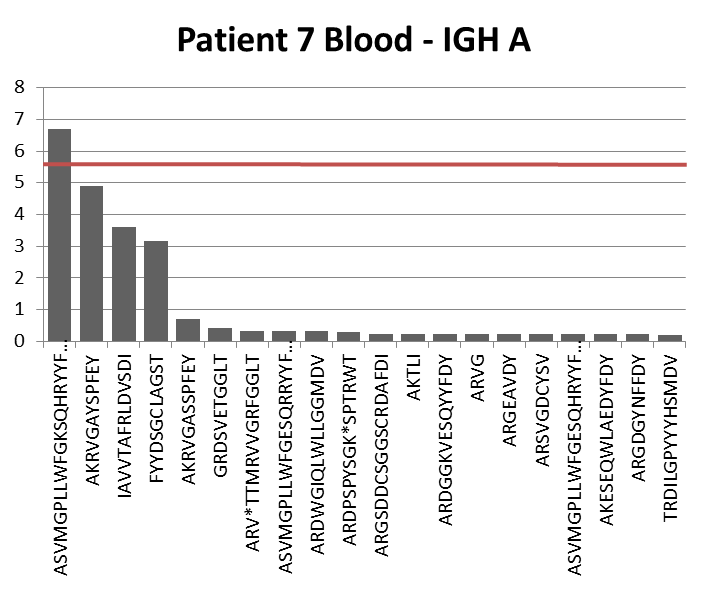


**NO IGH PRIMER SET 2 AND 3 AND TRB PRIMER SET 2 FOR PATIENT 7 BLOOD**


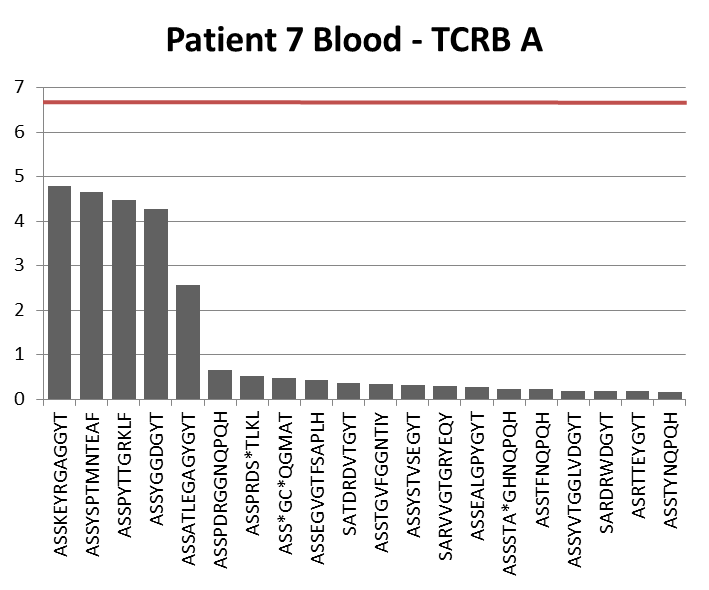


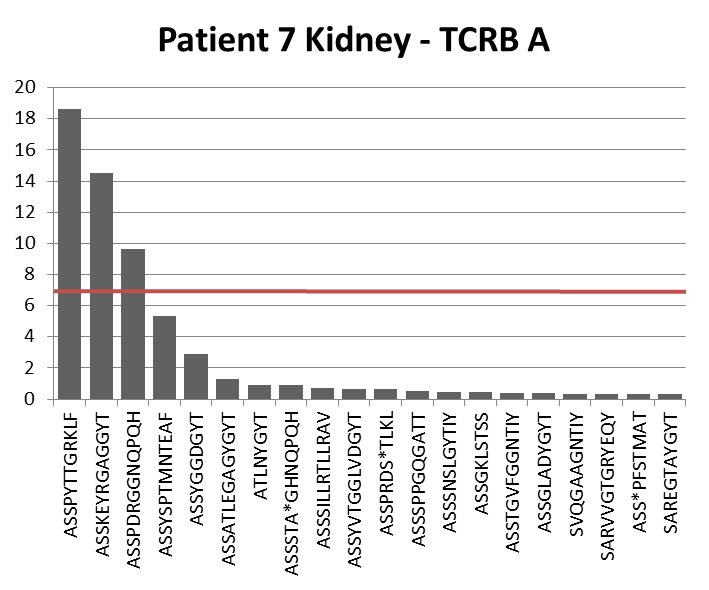

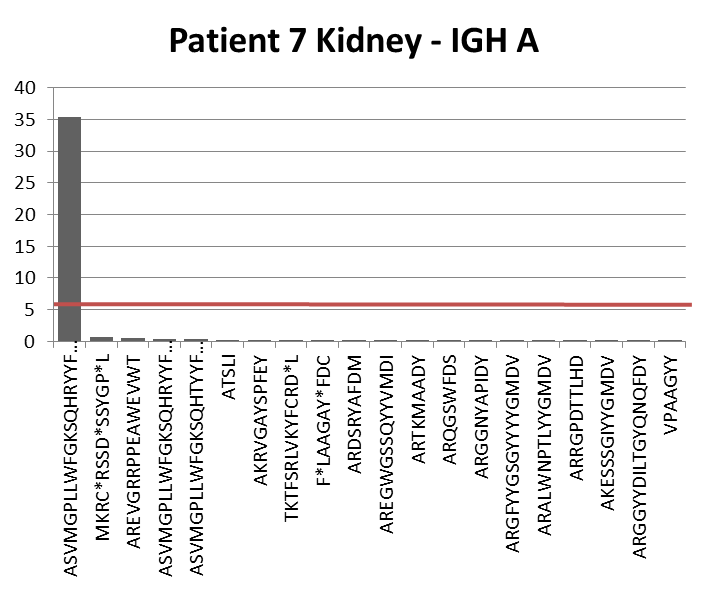
 **NO IGH PRIMER SET 2 AND 3 AND TRB PRIMER SET 2 FOR PATIENT 7 KIDNEY**


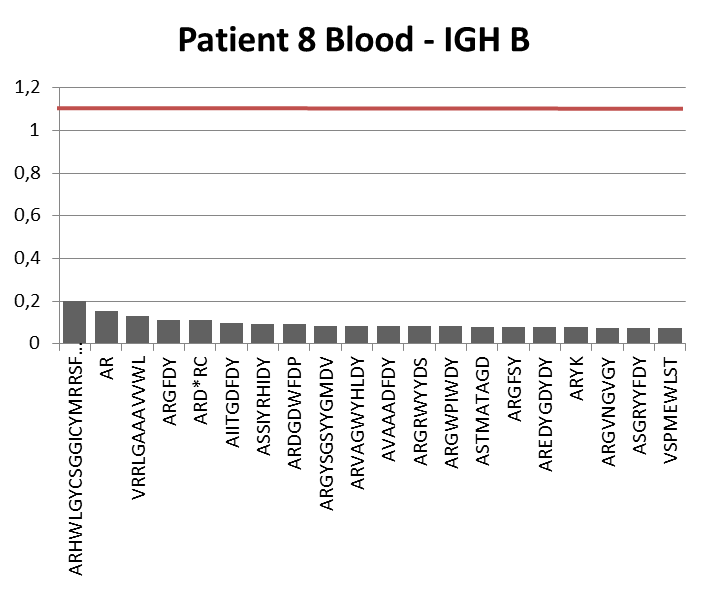

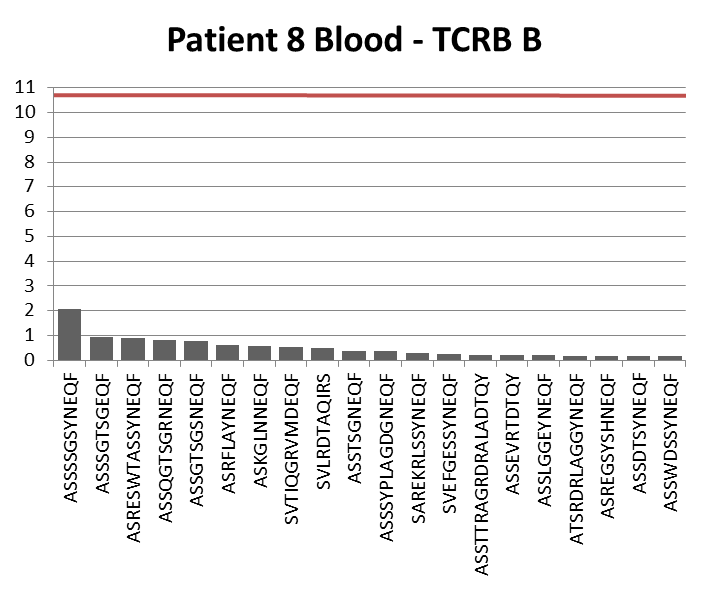

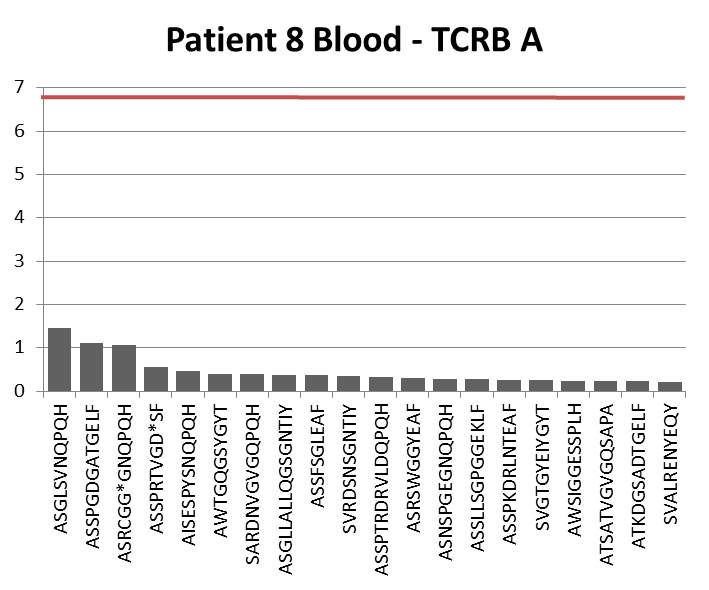

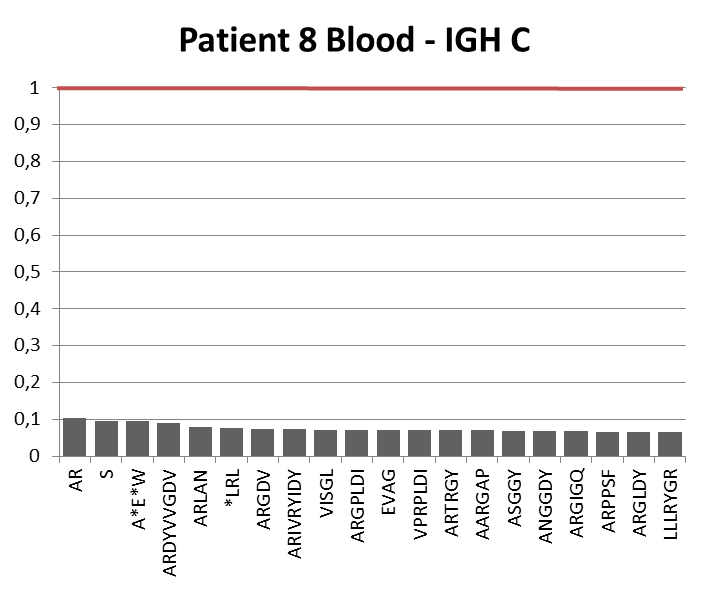

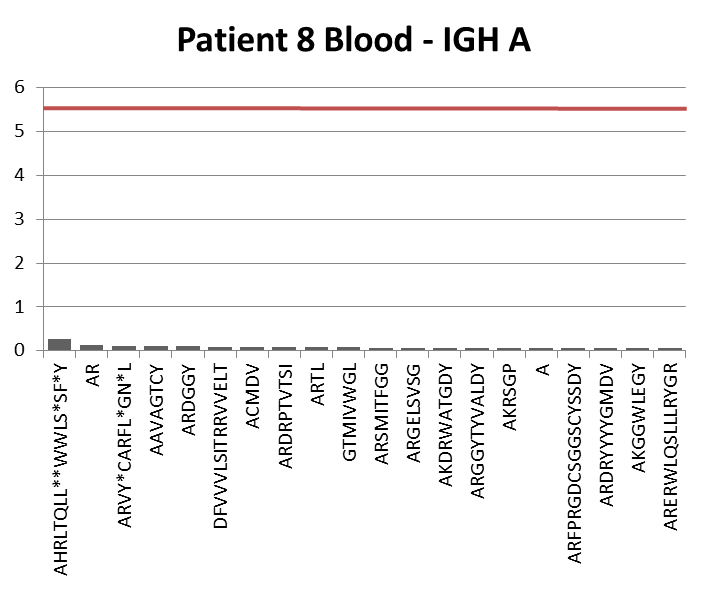


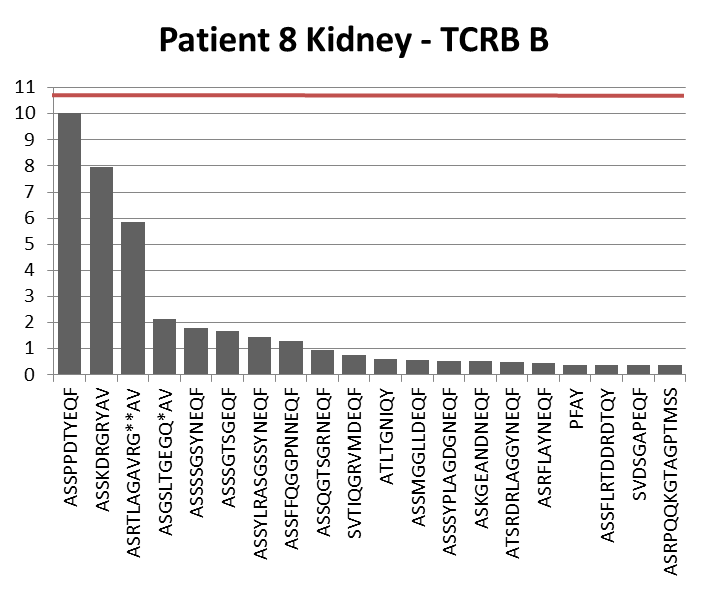

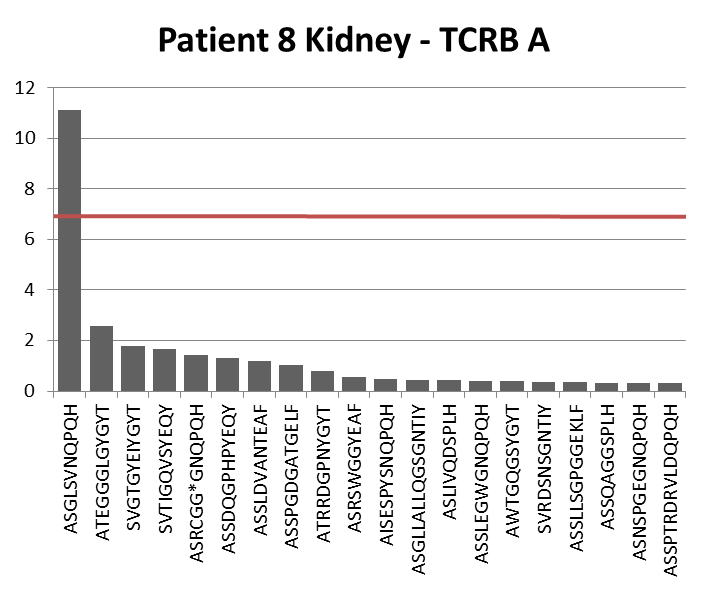

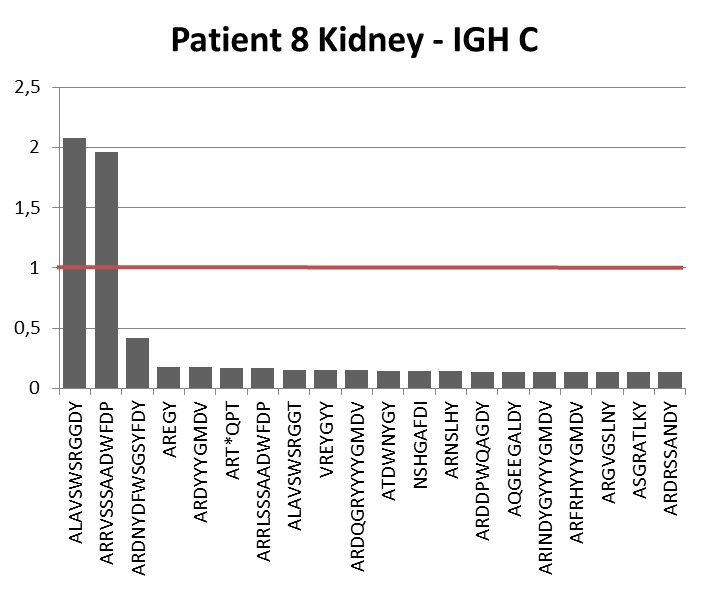

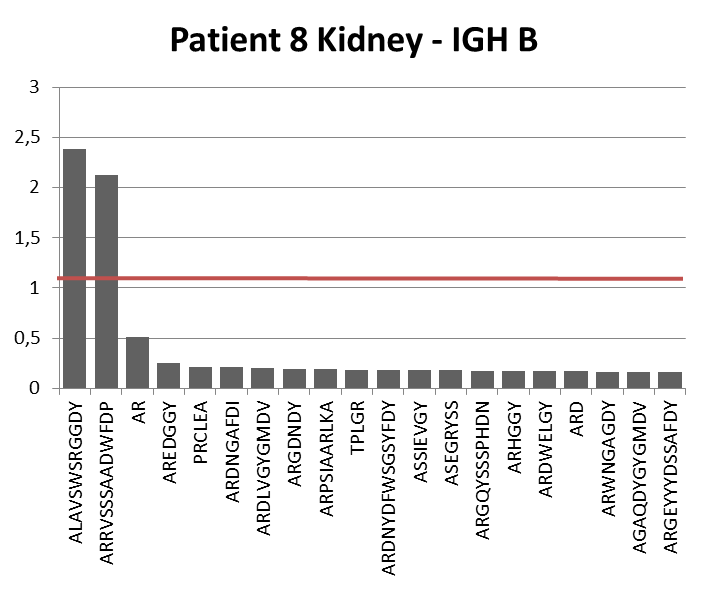

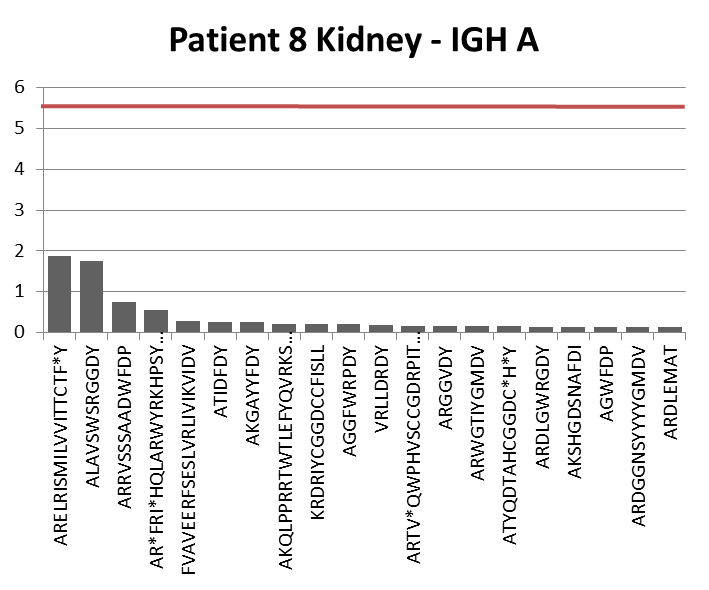


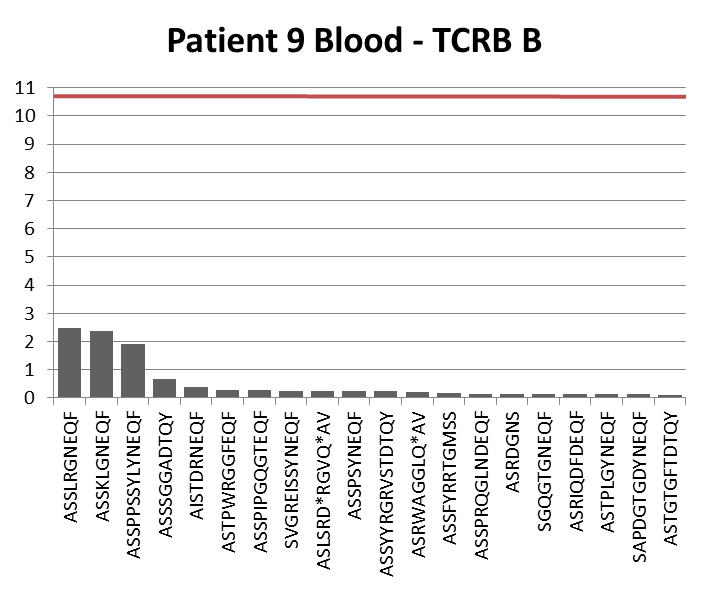

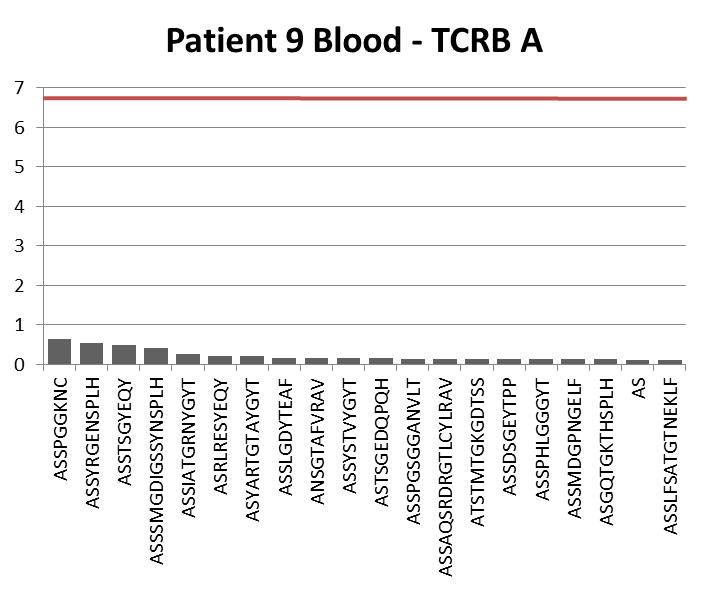

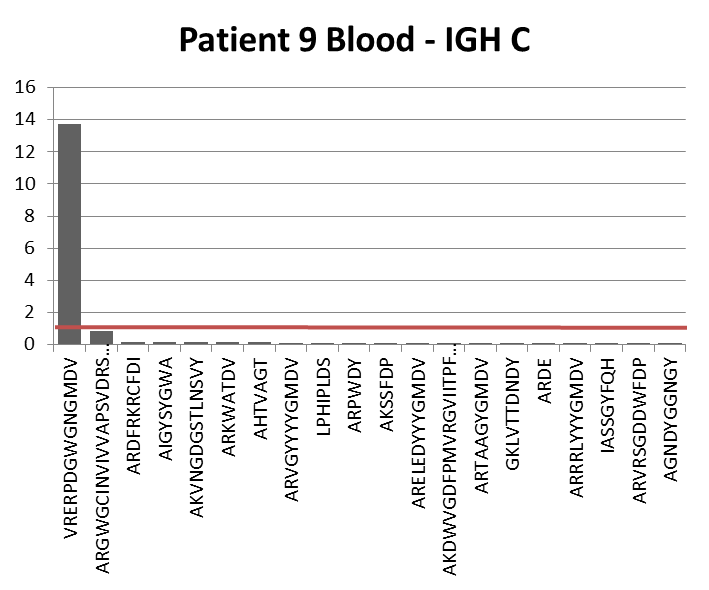

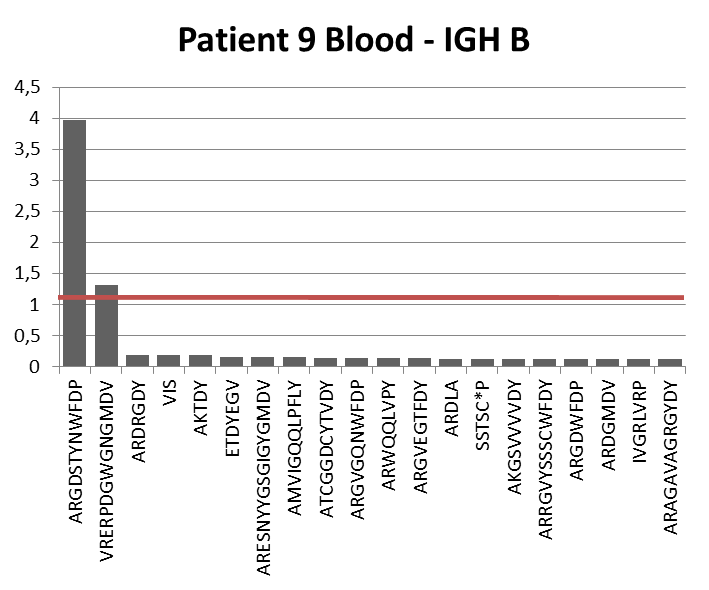

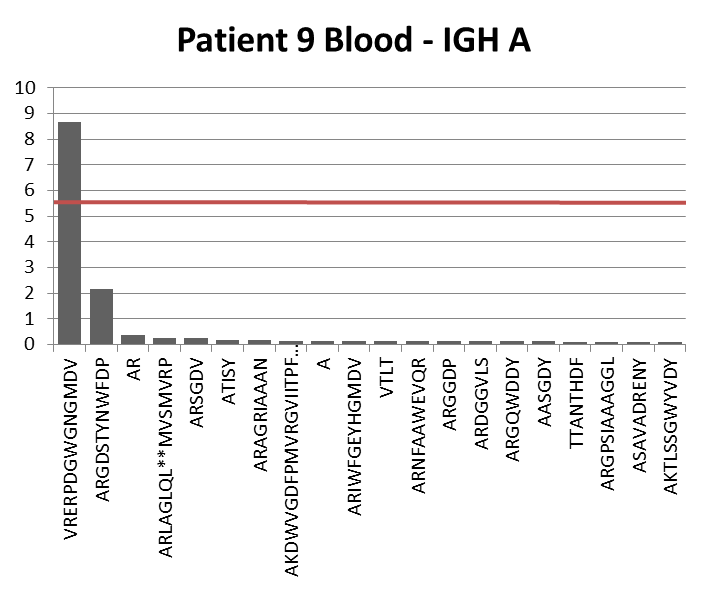


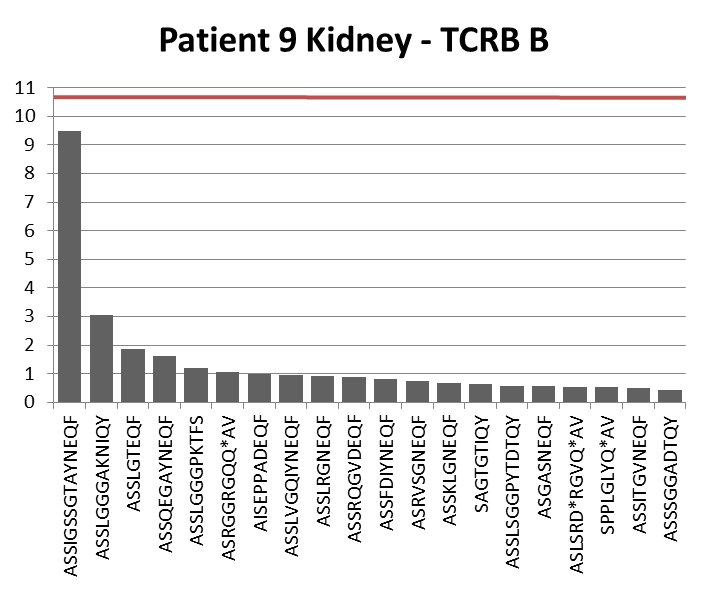

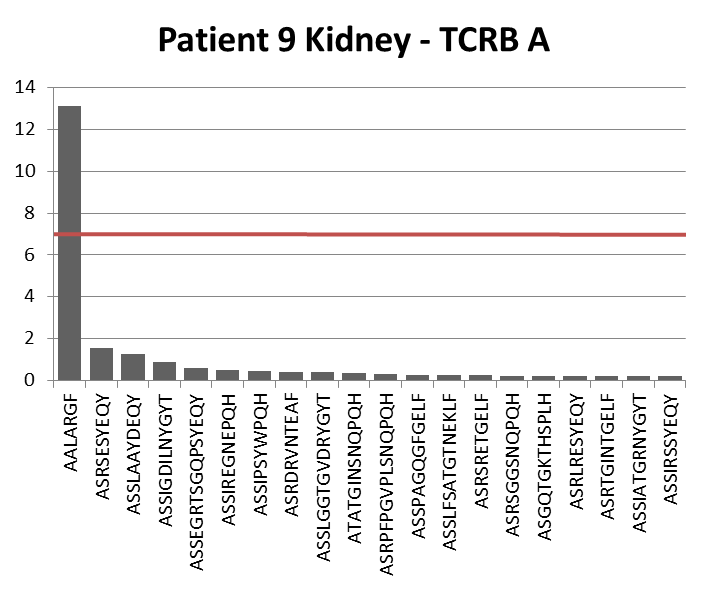

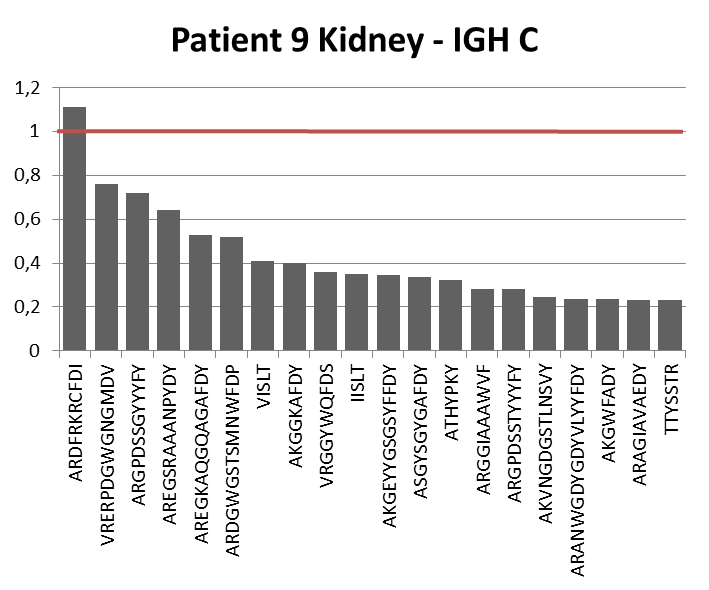

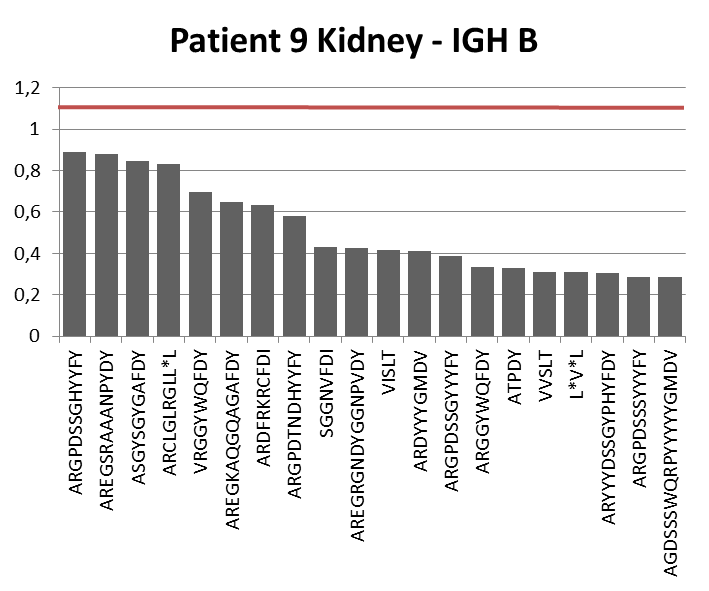

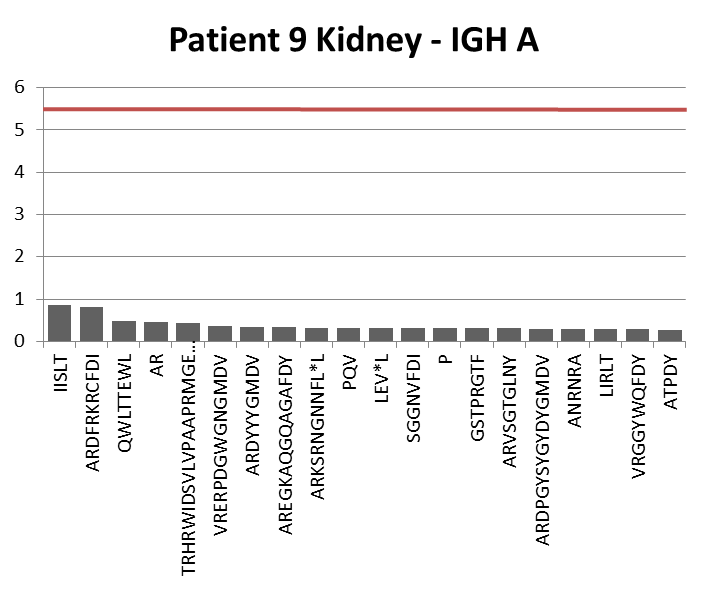


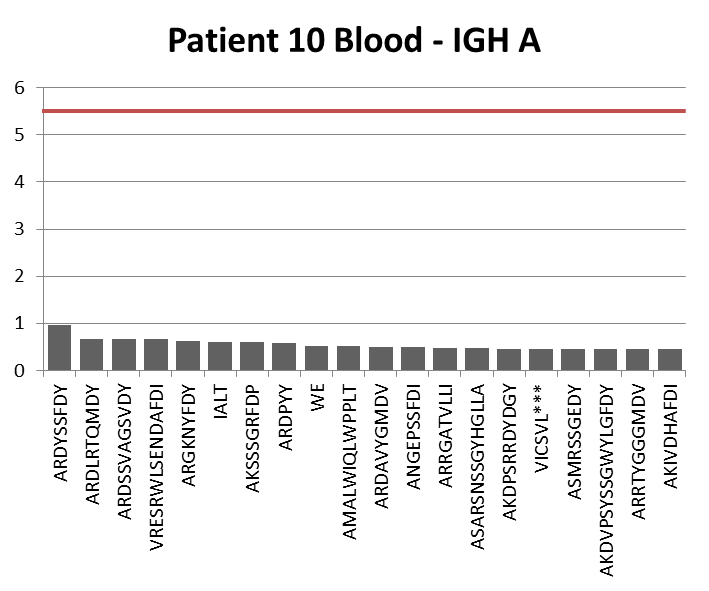

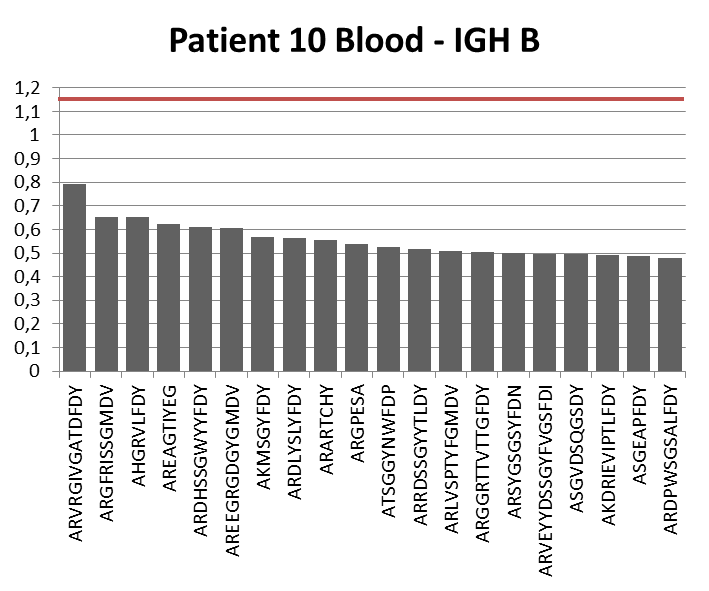

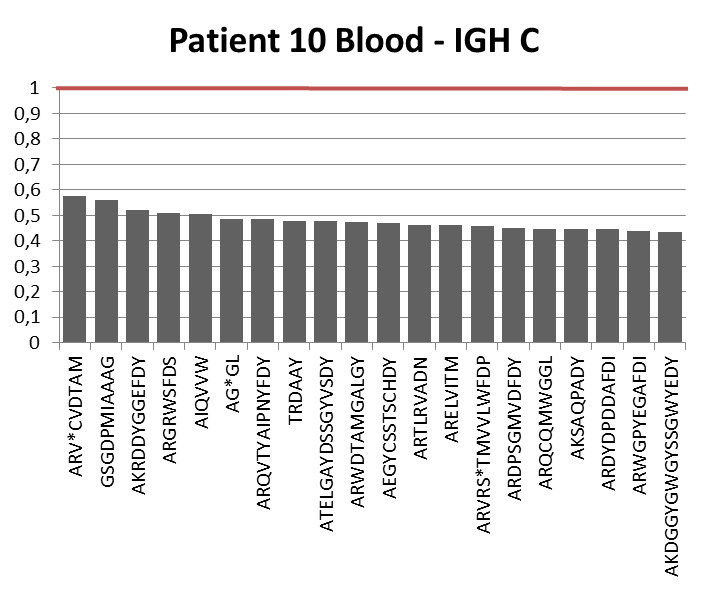


**TRB PATIENT 10 BLOOD ARE ONLY FOR CD8+**
