# Supplementary material for: Immune Repertoire Profiling Reveals that Clonally Expanded B and T Cells Infiltrating Diseased Human Kidneys Can Also Be Tracked in Blood
Source: PLoS One. 2015 Nov 23;10(11):e0143125. doi: 10.1371/journal.pone.0143125 (PMC4658119; doi:10.1371/journal.pone.0143125)

**S8 Fig. Technical replicates.**

**
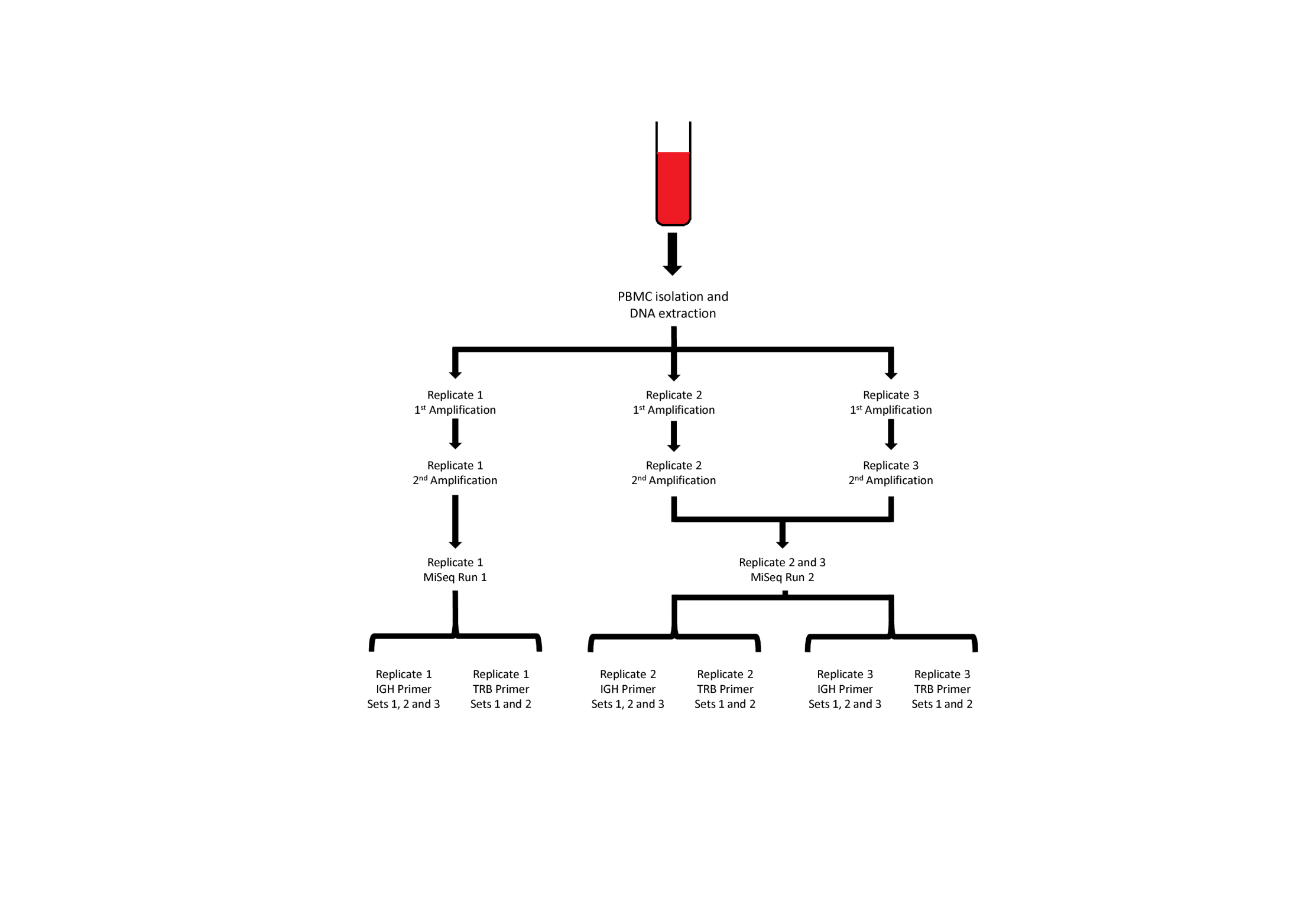
Experiment design.** We prepared three technical replicates of a clinical sample (blood of patient 1) starting from the same DNA extraction. One replicate was independently sequenced and the other two were sequenced in the same MiSeq Run. From each replicate, 5 different tubes (3 B cell primer sets and 2 T cell primer sets) were sequenced.

**Replicates general information.** Number of reads, clonotypes given after sequencing, clonotypes corrected after diversity calculation (Schaller et al. BMC Bioinformatics 2015) and percentage of corrected clonotypes selected from the sequencing output after excluding sequencing errors.

**Pearson’s sample correlation coefficient (r)** for each primer set.

**Top 10 highest abundant clones (top 10) comparison for each primer set.** Each color represents the same CDR3 clonotype in the different replicates within the top 10. White means that the clone is not found within the top 10 of the other replicates.


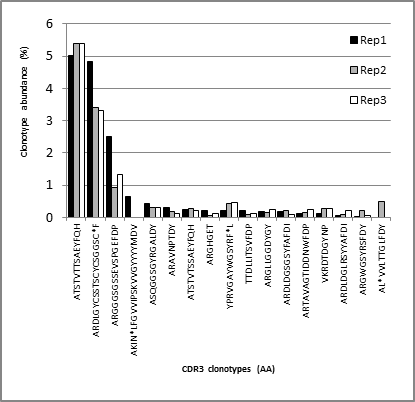


**IGH Primer Set 1**


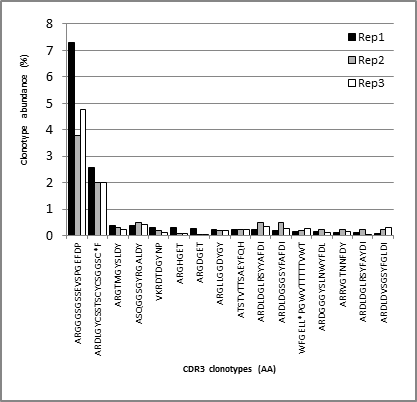


**IGH Primer Set 2**


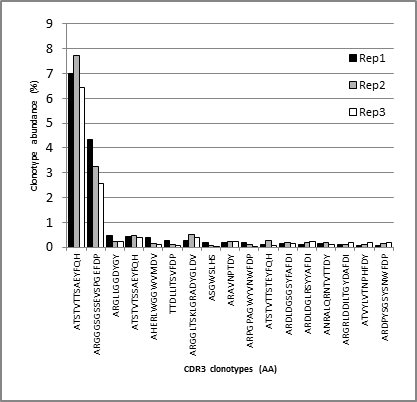


**IGH Primer Set 3**

**TRB Primer Set 1**


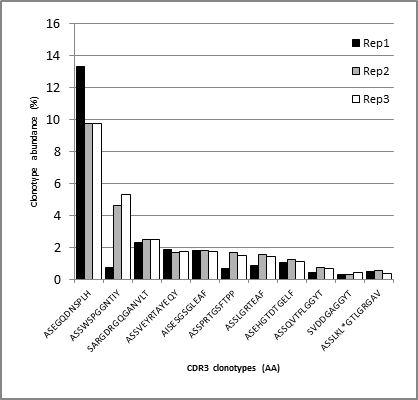


**TRB Primer Set 2**


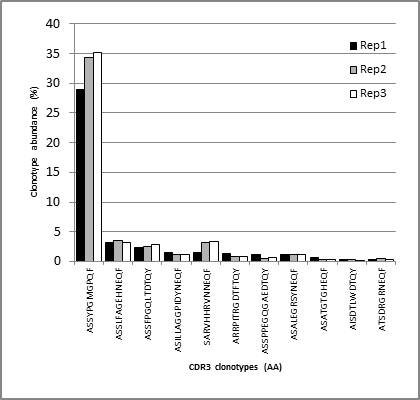

Supplement: S8 Fig — In the experiment design for three replicates with B and T cell expansions, we compared not only if there is a difference between replicates due to PCR or hands-on errors, but also if the sequencing run can bias the reproducibility of the protocol. Pearson’s sample correlation coefficient for each primer set displays high correlation values between replicates in both, B and T cells, with no remarkable differences between sequencing runs. Only T cell primer set 1 correlation shows lower values for replicate 1 compared to replicates 2 or 3 due to a specific clone dropout in replicate 1 (ASSWSPGGNTIY). The top 10 highest abundant clonotype comparison for each primer set shows the variability of the same CDR3-based clonotype percentage among replicates. Each clonotype is highlighted in one different color for each primer set to compare the concordance in rank position between replicates. (DOCX) [file pone.0143125.s008.docx]
