# Supplementary material for: Immune Repertoire Profiling Reveals that Clonally Expanded B and T Cells Infiltrating Diseased Human Kidneys Can Also Be Tracked in Blood
Source: PLoS One. 2015 Nov 23;10(11):e0143125. doi: 10.1371/journal.pone.0143125 (PMC4658119; doi:10.1371/journal.pone.0143125)
Supplement: S1 Table — The 10 patients included in the study are grouped by age range (using 10-year block groups) and by cause of nephrectomy. The sampled material used for each individual patient is listed for the peripheral blood (ml), kidney tissue (cm x cm) and urine (ml). A histological description of each piece of tissue is also provided. (DOCX) [file pone.0143125.s010.docx]

**S1 Table. Clinical data of patients**
