# Supplementary material for: Immune Repertoire Profiling Reveals that Clonally Expanded B and T Cells Infiltrating Diseased Human Kidneys Can Also Be Tracked in Blood
Source: PLoS One. 2015 Nov 23;10(11):e0143125. doi: 10.1371/journal.pone.0143125 (PMC4658119; doi:10.1371/journal.pone.0143125)
Supplement: S2 Table — The reaction of lymphocyte subpopulations was evaluated with monoclonal antibodies (mAb) by 4 to 5 color FCM analysis on a FACSAria III (Becton Dickinson). Three FCM tubes were used for each patient and material (Tube 1: CD8-FITC, CD3-PE, CD4-APC, CD45-HorizonV500; Tube 2: CD19-FITC, CD56-PE, CD3-APC-A, CD45-HorizonV500; and Tube 3: CD20-FITC, CD19-APC-A, CD45-HorizonV500). Data was analyzed using FACSDiva software Version 7.0 (BD Biosciences). (DOCX) [file pone.0143125.s011.docx]

| **Marker** | **Clone** | **Fluorochrome** | **Manufacturer** |
| --- | --- | --- | --- |
| CD3 | SK7 | PE | Becton Dickinson |
| CD3 | SK7 | APC-A | eBioscience |
| CD4 | SK3 | APC | Becton Dickinson |
| CD8 | SK1 | FITC | Becton Dickinson |
| CD19 | HIB18 | FITC | BD Pharmingen |
| CD19 | SJ25C1 | APC-A | BD Pharmingen |
| CD20 | 2H7 | FITC | BD Pharmingen |
| CD45 | HI30 | Horizon V500 | BD Biosciences |
| CD56 | B159 | PE | BD Pharmingen |

**S2 Table. Antibodies used for FACS**
