# Supplementary material for: Immune Repertoire Profiling Reveals that Clonally Expanded B and T Cells Infiltrating Diseased Human Kidneys Can Also Be Tracked in Blood
Source: PLoS One. 2015 Nov 23;10(11):e0143125. doi: 10.1371/journal.pone.0143125 (PMC4658119; doi:10.1371/journal.pone.0143125)
Supplement: S3 Table — For expansion threshold calculation, we considered the percent values of the most frequent clonotypes in blood of healthy probands for B cell primer sets 1, 2 and 3, and for T cell primer sets 1 and 2. In order to determine representative thresholds, the averages of all healthy probands’ highest clonotypes were calculated for each B and T cell primer set and the resulting value was multiplied by 5. All values above these thresholds were considered expanded in our study. We considered 1% as the lower limit to feel confident about the value, so for B cell primer set 3 we increased the threshold from 0.69% to 1%. The same values were used for blood and kidney (we had no access to healthy renal tissue). (DOCX) [file pone.0143125.s012.docx]

**S3 Table. Most frequent clonotype from healthy probands.**
