# Supplementary material for: Immune Repertoire Profiling Reveals that Clonally Expanded B and T Cells Infiltrating Diseased Human Kidneys Can Also Be Tracked in Blood
Source: PLoS One. 2015 Nov 23;10(11):e0143125. doi: 10.1371/journal.pone.0143125 (PMC4658119; doi:10.1371/journal.pone.0143125)
Supplement: S4 Table — Comparison of abundance (in %) and rank position of the clonotypes (rank refers to position depending on abundant frequencies) was considered to be expanded in our study for the three B cell primer sets. Collapse of the data for Table 1 was done selecting the highest percentage between primer sets and the same primer set in the other compartment. Expanded clonotypes that could not be detected in the other compartment or primer set were marked as not detected, n.d. (DOCX) [file pone.0143125.s013.docx]

**S4 Table. B cell comparison of all primer sets.**
