# Supplementary material for: Immune Repertoire Profiling Reveals that Clonally Expanded B and T Cells Infiltrating Diseased Human Kidneys Can Also Be Tracked in Blood
Source: PLoS One. 2015 Nov 23;10(11):e0143125. doi: 10.1371/journal.pone.0143125 (PMC4658119; doi:10.1371/journal.pone.0143125)
Supplement: S5 Table — Individual information is given for all primer sets in blood and kidney on the number of reads, the number of clonotypes from sequencing (CDR3-based), the corrected number of unique clonotypes [17] and the percentage of corrected clonotypes selected from the sequencing output after excluding sequencing errors. 1 stands for CD4+ sorted cells and 2 stands for CD8+ sorted cells. (DOCX) [file pone.0143125.s014.docx]

**S5 Table. Reads, clonotypes and diversity correction.**
