# Supplementary material for: Immune Repertoire Profiling Reveals that Clonally Expanded B and T Cells Infiltrating Diseased Human Kidneys Can Also Be Tracked in Blood
Source: PLoS One. 2015 Nov 23;10(11):e0143125. doi: 10.1371/journal.pone.0143125 (PMC4658119; doi:10.1371/journal.pone.0143125)
Supplement: S6 Table — Individual calculations for all patients (min. = 0; max. = 1) for all versus all CDR3-based clonotype comparisons, high abundant clones (top 20) comparison and low abundant clones (from position 21 to 1000) comparison. Patient 10 displays two values on the TR because the blood sample was sorted in CD4+ and CD8+ subpopulations; IG values for patient 6 were discarded due to low DNA concentration. In blue, average combination of primer sets 1, 2 and 3 for B cells and primer sets 1 and 2 for T cells are given. (DOCX) [file pone.0143125.s015.docx]

**S6 Table. Morisita-Horn similarity index calculation.**
