# Supplementary material for: Immune Repertoire Profiling Reveals that Clonally Expanded B and T Cells Infiltrating Diseased Human Kidneys Can Also Be Tracked in Blood
Source: PLoS One. 2015 Nov 23;10(11):e0143125. doi: 10.1371/journal.pone.0143125 (PMC4658119; doi:10.1371/journal.pone.0143125)
Supplement: S7 Table — Frequencies of lymphocyte subpopulations (T cells, cytotoxic T cells, helper T cells, NKT cells, NK cells and B cells) are given for every patient in blood and kidney as well as P-values for pairwise comparisons (paired two-sided t-test). (DOCX) [file pone.0143125.s016.docx]

**S7 Table. Lymphocyte subpopulations counted by FACS (in %).**
