# Supplementary material for: Immune Repertoire Profiling Reveals that Clonally Expanded B and T Cells Infiltrating Diseased Human Kidneys Can Also Be Tracked in Blood
Source: PLoS One. 2015 Nov 23;10(11):e0143125. doi: 10.1371/journal.pone.0143125 (PMC4658119; doi:10.1371/journal.pone.0143125)
Supplement: S8 Table — High values represent broader diversity. In order to remove sequencing error derived clonotypes we used the diversity calculation feature of IMEX [17]. Diversity was then calculated for patients’ blood and kidney and for healthy individuals’ blood using the inverse Simpson’s diversity formula described in the material and methods section. (DOCX) [file pone.0143125.s017.docx]

**S8 Table. Inverse Simpson’s diversity index.**
