# Supplementary material for: Immune Repertoire Profiling Reveals that Clonally Expanded B and T Cells Infiltrating Diseased Human Kidneys Can Also Be Tracked in Blood
Source: PLoS One. 2015 Nov 23;10(11):e0143125. doi: 10.1371/journal.pone.0143125 (PMC4658119; doi:10.1371/journal.pone.0143125)
Supplement: S9 Table — (1) divided in T cells (CD4+ and CD8+) and B cells. B cells in patient 6 were discarded due to low input. The cell input was calculated using FACS results percentages (2) Cell input of healthy probands using cell proportions from literature (70% T cells and 30% B cells). (3) P-values for comparisons between cell types and compartments using paired two-sided T-test. The calculation was performed using the following formula: Cell input = [X]μg/ml*FACS*(1000pg/μg)/(6.6pg/cell)*30μl. [X]μg/ml is the O.D. concentration of the extracted DNA from the isolated MNCs. FACS is the percentage of cells from each cell type. 6.6pg/cell is the standard amount of DNA in pg per cell. 30μl is the input volume for 1st PCR amplification. (DOCX) [file pone.0143125.s018.docx]

**S9 Table. Cell input**

(1)

(2)

(3)
